# Understanding the development of bipolar disorder and borderline personality disorder in young people: a meta-review of systematic reviews

**DOI:** 10.1017/S0033291722003002

**Published:** 2022-12

**Authors:** Buse Beril Durdurak, Nada Altaweel, Rachel Upthegrove, Steven Marwaha

**Affiliations:** 1 Institute for Mental Health, School of Psychology, University of Birmingham, Birmingham, UK; 2 Early Intervention Service, Birmingham Women's and Children's NHS Foundation Trust, Birmingham, UK; 3 Specialist Mood Disorders Clinic, Birmingham and Solihull Mental Health NHS Foundation Trust, Birmingham, UK

**Keywords:** Bipolar disorder, borderline personality disorder, development, factor, meta-review

## Abstract

**Background:**

There is ongoing debate on the nosological position of bipolar disorder (BD) and borderline personality disorder (BPD). Identifying the unique and shared risks, developmental pathways, and symptoms in emerging BD and BPD could help the field refine aetiological hypotheses and improve the prediction of the onset of these disorders. This study aimed to: (a) systematically synthesise the available evidence from systematic reviews (SRs) and meta-analyses (MAs) concerning environmental, psychosocial, biological, and clinical factors leading to the emergence of BD and BPD; (b) identify the main differences and common features between the two disorders to characterise their complex interplay and, (c) highlight remaining evidence gaps.

**Methods:**

Data sources were; PubMed, PsychINFO, Embase, Cochrane, CINAHL, Medline, ISI Web of Science. Overlap of included SRs/MAs was assessed using the corrected covered area process. The methodological quality of each included SR and MA was assessed using the AMSTAR.

**Results:**

22 SRs and MAs involving 249 prospective studies met eligibility criteria. Results demonstrated that family history of psychopathology, affective instability, attention deficit hyperactivity disorder, anxiety disorders, depression, sleep disturbances, substance abuse, psychotic symptoms, suicidality, childhood adversity and temperament were common predisposing factors across both disorders. There are also distinct factors specific to emerging BD or BPD.

**Conclusions:**

Prospective studies are required to increase our understanding of the development of BD and BPD onset and their complex interplay by concurrently examining multiple measures in BD and BPD at-risk populations.

## Introduction

Differential diagnosis between bipolar disorder (BD) and borderline personality disorder (BPD) is often difficult due to the high frequency of comorbidity and overlap of symptoms between the two disorders (Baryshnikov et al., 2015). The prevalence of BD and BPD was 21.6% and 18.5% respectively (Fornaro et al. 2016). There is an ongoing debate over whether BPD should be considered as part of the spectrum of BD disorders (Akiskal, 2004; Benazzi, 2006; Deltito et al., 2001; McGlashan, 1983; Zimmerman, Ruggero, Chelminski, & Young, 2009) or not (Bassett et al., 2017; Paris & Black, 2015).

Although BD and BPD are defined as distinct psychopathologies in Diagnostic and Statistical Manual of Mental Disorders (DSM-5; American Psychiatric Association, 2013) and International Classification of Diseases (ICD; World Health Organisation, 2015), there are many common features between these disorders that contribute to diagnostic confusion. The common features that are frequently stated in the literature are affective instability (AI), impulsivity, troubled relationships, distractibility, irritability, suicidality, flight of thoughts and childhood adversity (John & Sharma, 2009). The clinical evidence against for the existence of a ‘bipolar-borderline continuum’ argue that although there are common symptoms between the two, they present these traits differently (e.g. Henry et al., 2001; Renaud, Corbalan, and Beaulieu, 2012). However, in practical terms these distinctions are far from clear, particularly when there is no history of manic episodes (Sanches, 2019). For instance, differences in intensity or frequency might exist when comparing the two conditions for AI, but it is also unclear whether the anger and anxiety that BPD patients experience are distinct in nature than the mood experienced in a dysphoric or irritable manic state (MacKinnon & Pies, 2006). A concern that has compounded these issues is the increasing recognition that mood can be highly variable in people with BD outside of frank manic or depressive episodes, a move away from the traditional view of euthymia in BD (Bonsall, Wallace-Hadrill, Geddes, Goodwin, & Holmes, 2012).

There is also ambiguity of the relationship between impulsiveness and the diagnostic syndromes between BD and BPD. Impulsivity is considered to be a stable symptom of BPD diagnosis like AI (Wilson & Stanley, 2007). However, Zanarini, Frankenburg, Hennen, Reich, and Silk (2005) in their longitudinal study found that impulsive traits were likely to remit in the future in BPD patients. On the contrary, although impulsivity is considered to be episodic in nature in BD, Swann, Pazzaglia, Nicholls, Dougherty, and Moeller (2003) found that impulsivity had both state and trait related aspects in BD patients.

Practitioners are facing challenges when they attempt to classify the symptoms of these disorders based on the DSM's and ICD's classifications because these disorders sometimes do not fall clearly into state- and trait- like categories resulting in under, over or misdiagnoses (Ruggero, Zimmerman, Chelminski, & Young, 2010; Wilson & Stanley, 2007; Zimmerman, Ruggero, Chelminski, & Young, 2008). Investigating the early signs and symptoms in leading to the development of BD and BPD could be beneficial to clarify which symptoms are the most sensitive and specific markers of these disorders in young people. Whilst these studies will help determine their pathogenesis, they could also let us understand whether they are distinct clinical entities. Additionally, young people's affinity to impulsive and self-harming behaviour places them at-risk for adverse health outcomes (Kaess, Brunner, & Chanen, 2014). Both BD and BPD are associated with severe impairment in psychosocial functioning and a high suicide rate (Zimmerman et al., 2014). The risk for suicide among individuals diagnosed with BD are up to 20–30 times greater than that for the general population (Pompili et al., 2013) while the lifetime suicide rate for BPD is estimated to be 8% (Pompili, Girardi, Ruberto, & Tatarelli, 2005). Thus, it is critically important to synthesise and evaluate the current evidence which examine the interaction between environmental, biological, sociocultural, and clinical precursor signs and symptoms and their relationship to onset of BPD and BD diagnosis.

Many studies, including systematic reviews (SRs) and meta-analyses (MAs), have examined factors related to emerging BD and BPD (e.g. Ratheesh et al., 2017; Stepp, Lazarus, and Byrd, 2016). However, none of them compared BD and BPD at-risk populations concurrently, probably because there is still no consensus around BD prodrome and emerging BPD traits (Berk et al., 2007; Chanen & Kaess, 2011; Skjelstad, Malt, & Holte, 2010). Thus, a meta review of reviews approach to synthesising this evidence was adopted to be able to make this comparison. By synthesising the evidence now, future studies can investigate these common and distinct features cross-diagnostically in at-risk BD and BPD populations to provide better clinical diagnosis and treatment.

The aim of this review is to systematically assess SRs and MAs from prospective studies on factors that are associated with the early course of BD and BPD symptoms, features, or onset to be able to understand the differences and similarities in developmental pathways to these disorders and to determine whether they are two distinct clinical entities or belong on a continuum within the affective spectrum.

## Methods

### Protocol and registration

The protocol was registered with PROSPERO in January 2021 (registration no. CRD42021235193).

### Search strategy

The most current version of the Preferred Reporting Items for Systematic Reviews and Meta-Analyses (PRISMA) guidelines for conducting SRs Moher et al.'s (2015) guidelines were used as a framework (Page et al., 2021). An extensive search of papers catalogued in Embase, PsychINFO, PubMed, CINAHL, COCHRANE, ISI Web of Science, Medline databases was conducted in January 2021. Search terms were agreed by the authors following a scoping search. The terms were then modified following advice from a librarian and field experts. The search was conducted by combining six groups of terms using medical subject headings (MeSH) and text words (see online Supplementary) relating to; borderline personality disorder (e.g. ‘borderline personality’), bipolar disorder (e.g. ‘bipolar disorder’), risk factors/onset (e.g. ‘develop*’ OR risk*), longitudinal studies (e.g. ‘prospective study’), youth (e.g. ‘young adult’) and systematic reviews/meta-analyses (e.g. ‘systematic review’). In addition, we hand searched 10 specialty journals and reference lists. We also examined the first 30 pages in Google Scholar using the terms ‘bipolar AND borderline personality AND systematic review’. The search was updated in February 2022.

### Eligibility criteria

Inclusion criteria were:

1. SRs or MAs containing at least one relevant prospective study with at least 2 structured clinical assessments and diagnostic outcome at follow-up of BD or BPD onset, prodrome, features or symptoms
2. Measure the precursor and/or vulnerability factor related to the BD/BPD outcomes prior to the outcome assessment of BD or BPD
3. Include a clinical, high-risk or community population
4. Include studies that assess BD or BPD through fully, semi-, or unstructured interviews administered by mental health professionals, symptom checklists, self-reports, interviews, or self-reported questionnaires that are based on standard classification systems such as, the International Classification of Diseases (ICD; World Health Organisation, 2015) or the Diagnostic and Statistical Manual of Mental Disorders (DSM; American Psychiatric Association, 2013)
5. Include studies reporting group comparisons between participants with BD or BPD and healthy or clinical control on any factor related to the BD or BPD outcome

Exclusion criteria were:

1. Reviews including only intervention, cross-sectional, or other studies where the exposure was collected retrospectively and no relevant prospective study
2. Dissertation papers, books, book chapters, editorials, letters, or conference proceedings
3. Reviews including studies that were not reporting precursors of transitions or symptoms/features
4. Genetic studies
5. Full text of the manuscript is not available

### Study selection and data management

A bespoke data extraction form was developed in Microsoft Excel prior manuscript review. Two independent authors (BD, NA) performed all the initial screening steps on the pre-defined eligibility criteria, and disagreements were solved through discussion with a third reviewer (SM). No publication or language restrictions were applied. Title and abstract screening were conducted using the Endnote X9 reference management tool for full text retrieval. Authors independently searched the full-text articles for inclusion in the review. BD and NA managed and extracted relevant data in duplicate from each eligible study on the extraction form relating to relevant study information (e.g. sample characteristics, aims, number of databases sourced and searched, type of factor studied and outcomes) and risk of bias quality assessment. The results of the extracted data were then cross-checked.

### Risk of bias assessment

Two authors (BD, NA) independently assessed the methodological quality of each included SR and MA using the Assessment of Multiple Systematic Reviews (AMSTAR; Shea et al., 2007).

### Data synthesis and analysis

Data were qualitatively synthesised within the review as the data were not suitable for quantitative synthesis due to the high heterogeneity among reviews.

### Overlapping data

Overlap of included SRs/MAs was assessed using the corrected covered area [CCA; Pieper, Antoine, Mathes, Neugebauer, and Eikermann (2014)]. Pieper et al.'s (2014) protocol according to 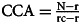 was followed, where N is the total number of included studies in SRs/MAs (including double counting), r is the number of primary studies, and c is the number of SRs/MAs. Overlap thresholds were used for interpretations of overlapping data; 0–5% – slight, 6–10% – moderate, 11–15% – high, >15% – very high (Pieper et al., 2014). For each disorder, a citation matrix and pairwise CCA tables were provided to address the overlap.

## Results

### Description of studies

**Fig. 1.**
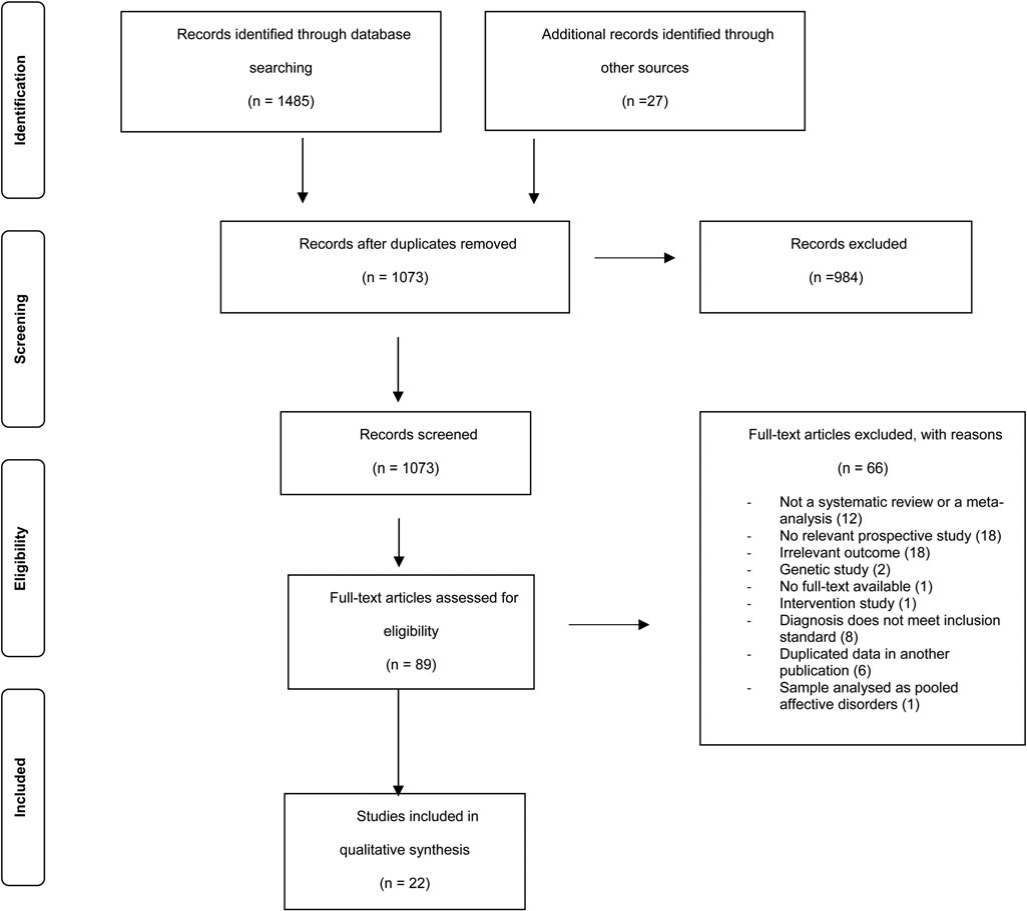
Flowchart of main search strategy and article selection for systematic review of review.

As shown in the PRISMA flow chart (Fig. 1), the literature search yielded 1485 records, 1073 were screened after duplication and 89 retrieved in full text. 66 articles were subsequently excluded with reasons (see online Supplementary Table S5) leaving 22 SRs and MAs to be synthesised in this SR of reviews.

Online Supplementary Tables S1 and S2 summarise the main characteristics of the 22 eligible SRs/MAs. The studies were published between 2011 and 2022. Nine studies are SRs and MAs, ten studies conducted SR of the literature with narrative syntheses of the results, and three performed MAs. The 22 reviews varied by population and outcomes.

In BD at-risk studies, three reviews studied individuals with depression who later showed (hypo)manic symptoms, six reviews studied individuals who developed BD at follow-up or at-risk for BD, six reviews studied high-risk offspring of BD and two reviews studied BD cohort with a recent first episode of mania. Three studies examined multiple factors, one examined suicidality, one examined cannabis use, one examined aberrancy in white matter, one examined grey matter changes, one examined childhood adversity, four examined sleep alterations, one examined development of BD in patients with ADHD and three examined family history of BD.

In BPD at-risk studies, two studied individuals who showed BPD symptoms or features or diagnosed with BPD at follow-up and three studied individuals diagnosed with BPD at baseline or showed BPD features, symptoms or diagnosed with BPD at follow-up. Three studies examined several factors related to BPD outcomes, one examined neurobiological correlates and one examined sleep profile.

### Primary studies

Within the 22 SRs, there were 678 primary studies of which 249 met the eligibility criteria for this SR of reviews (see online Supplementary Tables S1 and S2 for the number of relevant prospective studies synthesised for each study). The other 428 primary studies were excluded mostly because they were not prospective studies, or the participants had a full-syndromal diagnosis at the first intake. In BD studies, there were 2 418 329 participants across all included primary studies, of whom 127 706 were included in this review. BPD studies included 125 406 participants in total, of whom 82 015 were eligible to include in this review. The methodology applied here is in line with Prousali et al.'s (2019) overview of reviews.

### Overlapping Data for BD and BPD Studies

The 17 included SRs and MAs for BD comprised 250 overlapping individual studies, of which 145 were unique. Five included SRs and MAs for BPD comprised 74 overlapping individual studies, of which 64 were unique. A citation matrix presenting all the included SRs and MAs on BD and BPD in columns and index publications in rows and pairwise CCA tables are provided in online Supplementary Tables S8, S9, Figs S1 and S2.
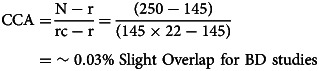

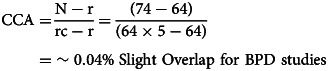


As CCA is estimated at %0.03 for BD studies and 0.04 for BPD studies, the overlap is in the low range reflecting a low risk of skewed reporting (Pieper et al., 2014).

### Assessment of methodological quality

Based on the findings from AMSTAR ratings that were performed to evaluate the methodological quality of the included SRs/MAs, the majority of the reviews were deemed to be high quality (see online Supplementary Tables S1 and S2, full assessments provided in online Supplementary Tables S6 and S7).

### Synthesis of results

Online Supplementary Tables S3 and S4 summarises the findings of SRs/MAs, respectively. A summary of the shared factors in emerging BD and BPD can be seen in Table 1. The evidence for developmental precursors that are prospectively related to BD and BPD outcomes are presented below except for the vulnerability factors which can be found in the online Supplementary.

**Table 1.** Similarities and differences in shared factors in emerging bipolar disorder and borderline personality disorder

Factor	Bipolar disorder onset	Borderline personality disorder onset	Bipolar disorder and borderline personality disorder onset
Childhood adversity	Higher rates of childhood adversity than participants who do not develop BD	Type of trauma is not limited to family caused trauma but also peer and romantic relationship related trauma in BPD onset	A shared factor, more marked in BPD onset
Sleep disturbances	Decreased need for sleep, middle insomnia, frequent night time awakenings, high energy in offspring of BD or as part of hypomanic symptoms Low social rhythm regularity, daytime dysfunction, hypersomnia and anergia, decreased REM sleep in cohorts reporting first onset of BD Time to fall asleep	Chronic nightmares (mediated by emotional and behavioural problems)	Both show difficulty falling asleep and waking earlier than desired
Depression	Bipolar depression rather than unipolar depression; presence of psychotic symptoms, feelings of guilt, hypomanic symptoms ‘Hypersomnic retarded depression’ Psychomotor retardation Chronic and frequent Higher recurrence rates Major depressive episodes	The course of depression is unipolar	Early onset of depression predicted both disorders but differentiating features are evident
Suicidality	Specific plan for committing a suicide Recurrent thoughts of death	Risk of self-harm	A shared factor but suicidal ideation was not stable after post-hospitalisation in BPD
Family psychopathology	A loaded family pedigree or family history of affective disorders spanning three generations	Paternal substance use disorder Maternal psychopathology (e.g. internalising and externalising disorders, BPD symptoms) Family history of psychiatric hospitalisation	Family history of mental illnesses is associated with later symptomatology in both disorders
Substance use disorders	Baseline cannabis use significantly predicted hypo/sub-threshold mania	BPD traits and SUD are correlates rather than causal antecedents of each other The role of behavioural disinhibition	SUDs are common to both disorders
Affective instability	Coexistent with baseline MDD Having ‘ups and downs’	Negative affectivity as affective instability, emotionality, aggressiveness/tantrums	AI is common to both disorders
Anxiety	Comorbid GAD Comorbid SP	Comorbid OCD	Comorbid anxiety disorders are common to both disorders
Temperament	Temperamental instability during MDD episodes Daydreaming Cyclothymic disorder Hyperthymic temperament Sensitivity, hyper alertness, excessive talk, talking too loudly, easily excited, poor attention, impaired role in school, somatic complaints	Higher levels of emotionality and low levels of sociability and shyness in middle childhood	High activity levels and poor psychosocial functioning are evident in both disorders
Psychotic symptoms	Psychosis NOS, schizotypal features and schizophrenia nuclear symptoms predicted conversion Comorbidity with MDD	Children in the extreme borderline group exhibited more psychotic symptoms	Symptoms common to both disorders but marked and detailed more in BD
ADHD	Switches were predicted by presence of parental mood disorder, school behaviour problems, and baseline comorbid conduct disorder	ADHD was predictive of both changes in BPD symptoms and onset	Shared feature; greater risk of BD and BPD occurrence in ADHD patients

ADHD, attention deficit hyperactivity disorder; BD, bipolar disorder; BPD, borderline personality disorder; GAD, generalised anxiety disorder; MDD, major depressive disorder; NOS, not otherwise specified; OCD, obsessive compulsive disorder; SP, social phobia; SUD, substance use sisorder.

Here we define the precursors (e.g. clinical symptoms, signs or syndromes, prodromes, biomarkers) and vulnerability risk factors (e.g. gender, family history of psychopathology, childhood adversity) as prospectively identified variables that increase the odds of later BD onset, BPD onset or features.

At-risk BD reviews differed in how they defined at-risk BD; the participants were either at familial (e.g. offspring of BD) and/or clinical risk (e.g. youth with subthreshold mania) for BD stages either at 0, 1a, 1b, or 2 (see online Supplementary Table S1). At-risk BPD reviews did not define an at-risk state for BPD but examined papers which included community or clinical samples who had BPD symptoms, features, or diagnosis at follow-up assessment (see online Supplementary Table S2).

### Biological factors

Three reviews reported data regarding differences in the white matter in a high-risk population (Hu, Stavish, Leibenluft, & Linke, 2020), neural reward circuit dysfunction (Bart, Titone, Ng, Nusslock, & Alloy, 2021) and longitudinal grey matter changes following first episode mania (Cahn, Keramatian, Frysch, Yatham, & Chakrabarty, 2021) compared to healthy controls. Hu et al. (2020) indicated that the trajectory of fractional anisotropy reduction did not differ significantly between high-risk young adults and controls. Cahn et al. (2021) found that adolescents with mania fail to exhibit normal increases in amygdala volume. No comparable studies were available for BPD. According to Bart et al.'s (2021) findings, lower right ventral striatum–left caudal anterior cingulate functional connectivity to loss and greater right pars orbitalis–orbitofrontal cortex functional connectivity to reward may be trait-level neural markers that may reflect risk for BD in at-risk youth. Additionally, lower parietal cortical thickness may lead to lower executive functioning and emotional regulation capacity and predispose to higher future mixed/mania and irritability.

### Clinical factors

#### Suicidality

Two reviews provided evidence for the association between suicidality and transition to BD (de Cardoso, Mondin, Azevedo, Toralles, & de Mattos Souza, 2018; Ratheesh et al., 2017). Both reviews reported inconsistent results for the association between suicidality and later BD onset; out of seven individual studies they included, only four found a significant association between suicidality and later BD.

For BPD, two reviews assessed this association. Stepp et al. (2016) found consistent prospective associations between suicidality and later BPD symptoms, whereas Winsper et al. (2016a) found suicidal ideation in adolescence was not stable after post-hospitalisation.

#### Affective instability

Data regarding the effect of AI on risk for BD was provided by three reviews (Faedda et al., 2015; Keramatian, Chakrabarty, Saraf, & Yatham, 2021; Ratheesh et al., 2017). All three studies found that AI predicted BD onset.

For BPD, both Stepp et al. (2016) and Skabeikyte and Barkauskiene (2021) found that AI and other negative affectivity symptoms such as emotionality and aggressiveness/tantrums predicted increases in mean levels of BPD features through adolescence.

#### Depression

Three reviews studied the relationship between depression and BD (Faedda et al., 2015; Keramatian et al., 2021; Ratheesh et al., 2017). They found that major depressive episodes, unipolar depression, depressive disorders NOS, mild depressive episodes, early onset of depression, longer and higher number of depressive episodes, greater loading of depressive symptoms, and higher recurrence rates, severity of depression, guilt, psychomotor retardation and AI coexistent with major depressive disorder (MDD) predicted transition to BD. There was also a significant association between age of onset of depression and later BD. The associations for recurrent MDD, chronicity of depression, atypical feature, hypersomnic-retarded depression, and conversion to BD was inconsistent.

For BPD, two reviews found significant association between depression and later BPD (Skabeikyte & Barkauskiene, 2021; Stepp et al., 2016). Additionally, decreases in depression severity predicted faster declines in average levels of BPD symptoms.

#### Subsyndromal hypomania

Evidence regarding hypomanic symptoms was available from three reviews for BD (Faedda et al., 2015; Keramatian et al., 2021; Ratheesh et al., 2017). They found that higher scores on Hypomanic Personality Scale (HPS), lifetime subsyndromal hypomanic symptoms and the combination of subclinical mania with subclinical psychosis at baseline significantly predicted transition to BD. Keramatian et al. (2021) also reported association between antidepressant associated subthreshold hypomanic episodes and transition to BD. No comparable studies were available for BPD.

#### Cyclothymia and bipolar NOS

Faedda et al. (2015) and Keramatian et al. (2021) indicated earlier onset Bipolar NOS predicted conversion to BD. Similarly, cyclothymic disorder and hyperthymic temperaments significantly predicted diagnoses of BD. No comparable studies were available for BPD.

#### Psychosis and psychotic symptoms

Two reviews assessed the associations between psychotic symptoms and later BD (Faedda et al., 2015; Ratheesh et al., 2017). They demonstrated that psychotic features significantly predicted conversion to BD. Higher conversion rates to BD were also found in people with psychosis NOS, schizotypal features, and schizophrenia nuclear symptoms but the results were inconsistent.

Only one review indicated significant associations between psychotic symptoms and later BPD (Stepp et al., 2016).

#### Substance use

Three reviews investigated the association between SUD and conversion to BD (Gibbs et al., 2015; Keramatian et al., 2021; Ratheesh et al., 2017). Gibbs et al. (2015) and Keramatian et al. (2021) reported consistent significant associations between cannabis use and hypo/sub-threshold mania symptoms. The magnitude of this relationship was small to medium. Ratheesh et al. (2017), on the other hand, reported inconsistent results among studies examining the association between SUD and later BD.

Three reviews assessed the associations between SUD and later BPD symptoms (Skabeikyte & Barkauskiene, 2021; Stepp et al., 2016; Winsper et al., 2016a). Skabeikyte and Barkauskiene (2021) indicated that SUD was predictive of changes in BPD features during adolescence whereas, Stepp et al. (2016) and Winsper et al. (2016a) found significant associations.

#### Antidepressant use

The association between antidepressant use and later BD was examined in two reviews; while Ratheesh et al. (2017) reported a non-significant relationship, Keramatian et al. (2021) found that exposure to antidepressants during follow-up was associated with increased risk of conversion. However, the evidence was available from only one primary study. No comparable studies were available for BPD.

#### Comorbidity with internalising and externalising disorders

Data regarding the association between comorbid disorders and later BD was available from three reviews (Brancati, Perugi, Milone, Masi, & Sesso, 2021; Keramatian et al., 2021; Ratheesh et al., 2017). Ratheesh et al. (2017) found comorbid social phobia and comorbid attention deficit hyperactivity disorder (ADHD) significantly predicted later BD onset. Results for comorbid generalised anxiety disorder and comorbid anxiety disorders as a group were inconsistent. Brancati et al. (2021) indicated a significantly greater risk of BD occurrence in ADHD patients *v.* healthy controls. Keramatian et al. (2021) found that anxiety disorders predicted conversion to BD in youth.

Evidence concerning the comorbidity with other mental health illnesses and later BPD symptoms were examined in three reviews (Skabeikyte & Barkauskiene, 2021; Stepp et al., 2016; Winsper et al., 2016a). They indicated childhood inattention, oppositional behaviour, anxiety symptoms, ADHD, somatisation significantly predicted the new onset of BPD and BPD symptom changes. They also reported significant associations between dissociation, conduct disorder, oppositional defiant disorder, depression, and later BPD symptoms. Individual social and physical aggression in childhood and comorbid obsessive compulsive disorder, on the contrary, did not predict BPD symptom changes.

#### Temperament/personality traits

Evidence regarding temperament in BD at-risk populations was available from one review (Keramatian et al., 2021). They found key symptoms to identify children with BD from well children in cohort samples; sensitivity, hyper alertness, anxiety/worry, somatic complaints, bold/intrusive, excessive talking, talking too loudly, decreased sleep, and impaired role in school.

Two reviews assessed the association between temperament/personality and later BPD (Skabeikyte & Barkauskiene, 2021; Stepp et al., 2016). Low levels of sociability, high levels of emotionality, activity and shyness in childhood, poor self-control, experiential avoidance, and disturbances in self-representation predicted later BPD symptoms.

#### Attachment

One review reported associations between attachment style and later BPD symptoms (Stepp et al., 2016). They indicated that disorganised/controlling behaviour in childhood and insecure attachment in peer relationships predicted BPD symptoms in adolescence. The results for attachment disorganisation and security in infancy and toddlerhood and later BPD symptoms were inconsistent. No comparable data were available for BD.

#### Impulsivity

Two reviews reported associations between impulsivity and later BPD symptoms (Skabeikyte & Barkauskiene, 2021; Stepp et al., 2016). They demonstrated that impulsivity (e.g. effortful control, low self- control, and low constraint) was predictive of BPD symptoms and new onset of BPD in adolescence. No comparable evidence was available for BD.

#### Sleep disturbances

Evidence regarding the association between sleep disorders and the risk of developing BD were available from five reviews (Keramatian et al., 2021; Pancheri et al., 2019; Ritter, Marx, Bauer, Lepold, & Pfennig, 2011; Scott et al., 2022; Scott, Kallestad, Vedaa, Sivertsen, & Etain, 2021). Pancheri et al. (2019) and Ritter et al. (2011) indicated that the offspring of patients with BD had sleep problems more frequently compared to not-at-risk offspring with a 30-fold increased risk to develop. The high-risk offspring with poor sleep were also more likely to develop BD. Keramatian et al. (2021), Scott et al. (2021), and Scott et al. (2022) found that individuals with a history any type of sleep disturbance had an increased odds of developing BD.

Only one review reported associations between sleep problems and later BPD (Winsper et al., 2017). They found that chronic nightmares and chronic sleep disturbances were significantly associated with later BPD.

#### Disruptive behaviour disorders (DBD)

DBD was associated with subsequent manic, mixed, or hypomanic episodes in one BD at-risk review (Keramatian et al., 2021). No comparable studies were available for BPD at-risk.

## Discussion

To the best of our knowledge, this is the first meta review of reviews aiming to understand the developmental pathways of BD and BPD, disorders that share some phenotypic features that could imply an overlap of aetiological mechanisms. 22 eligible reviews provided significant data about the factors which might contribute to the onset of BD or BPD. The current meta-review demonstrates that there are many ‘distinct’ clinical, environmental, psychosocial, and biological variables that can be found early in the course of BD and BPD, even in at-risk stages, but the disorders share a variety of clinical and vulnerability factors too. However, since these ‘distinct’ variables are evident only either in BD or BPD at-risk reviews, their distinctive value is speculative until further systematic longitudinal studies examine these factors in both disorders.

A notable and critical limitation of the literature is there were no studies comparing BD and BPD at-risk populations at the same time, compounding the difficulty of understanding specific BD or BPD developmental trajectory. Additionally, the neurobiological data from the BD at-risk studies are currently limited and there are no comparing studies done in BPD at-risk populations. This is why despite many previous commentary pieces on this issue (Bassett, 2012; Bayes et al., 2015; Deltito et al., 2001; Massó Rodriguez et al., 2021; Paris, 2004; Sanches, 2019; Smith, Muir, & Blackwood, 2004; Stone, 2006; Zimmerman & Morgan, 2013a, 2013b), in reality at the current time it is not possible to answer whether these disorders should be on the same affective continuum, or they should be regarded as separate nosological conditions.

Gender, differences in the white matter, changes in the amygdala, neural reward circuit dysfunctions, DBD, subsyndromal hypomania, cyclothymia or bipolar NOS, frequency and loading of affective symptoms, and antidepressant use were factors examined only in BD studies. Only changes in the amygdala, neural reward circuit dysfunctions, subdyndromal hypomania, cyclothymia or bipolar NOS, frequency and loading of affective symptoms consistently predicted BD transition. Interestingly there was no data available for emerging BPD for cyclothymia although previous research comparing participants with BD and BPD found that participants with BPD too show similar or even higher levels of abnormal cyclothymic temperament (Eich et al., 2014; Nilsson, Jørgensen, Straarup, & Licht, 2010). Previous evidence also shows that hypomanic days were reported frequently in both the BD and BPD subcohort (Socada, Söderholm, Rosenström, Ekelund, & Isometsä, 2021). However, based on our findings while hypomanic symptoms predicted BD onset (Faedda et al., 2015), there was no data pertaining to BPD onset. Likewise, accumulated evidence shows BD patients demonstrated enlarged amygdala (Soares & Young, 2016) while BPD patients showed decreased amygdala volumes (Perez-Rodriguez et al., 2018). Our results are not in accordance with these results because according to Cahn et al.'s (2021) findings, adolescents with mania failed to exhibit normal increases in amygdala volume. It is interesting that studies of people who are at-risk of developing BD has showed decreased in amygdala volumes while previous studies with BPD populations have also showed the same results. However, since there was no data on amygdala changes or hypomania symptoms in participants with BPD features, it is not possible to conclude at the moment whether both at-risk populations fail to exhibit normal increases in amygdala or hypomania is a shared feature.

Hu et al. (2020) indicated that differences in white matter integrity between high-risk individuals and control could occur in earlier childhood. This is consistent with a SR (Serafini et al., 2014) which found reduced corpus collosum volume and increased rates of deep white matter hyperintensities were more specific to paediatric BD in comparison to unipolar depression. There is however a need to replicate these findings with future longitudinal follow-up studies in both at-risk populations.

Amongst the factors examined in relation to transition to BD, the greatest amount of evidence was for family history of BD. Although inconsistencies in results were present in family history of BD studies (Keramatian et al., 2021; Lau et al., 2017; Narayan, Allen, Cullen, & Klimes-Dougan, 2013; Rasic, Hajek, Alda, & Uher, 2013; Ratheesh et al., 2017), some studies suggest that there might be a relative specificity of family history of BD to predicting later BD in MDD samples (Ratheesh et al., 2017; Vandeleur, Merikangas, Strippoli, Castelao, & Preisig, 2013). Likewise, the evidence in the current review was also inconsistent as some of the individual studies found a significant relationship between a family history of other mental illnesses (i.e. affective disorder or depression) and later BD conversion while the others did not. This is not surprising because although high-risk studies can be informative about transition to BD (DelBello & Geller, 2001; Duffy et al., 2011; McGuffin et al., 2003), these studies still have not supported the validity of the pre-pubertal BD phenotype and not all children of parents with BD develop BD or a mood disorder (Duffy, Carlson, Dubicka, & Hillegers, 2020; Malhi, Moore, & McGuffin, 2000; Malhi, Morris, Hamilton, Outhred, & Mannie, 2017).

Parenting behaviour/style, parent-child relationship quality, maternal characteristics, attachment, impulsivity, experiential avoidance, disturbances in self representation, dissociation, comorbid oppositional defiant disorder, comorbid conduct disorder, somatisation, general psychosocial functioning, and social and physical aggression in childhood were examined only in BPD studies. Attachment, impulsivity, experiential avoidance, disturbances in self representation, dissociation, comorbid oppositional defiant disorder, somatisation, general psychosocial functioning, social and physical aggression in childhood consistently predicted later BPD symptoms. Interestingly again, none of the BD at-risk reviews mentioned impulsivity although it is commonly found both in BD and BPD patients (di Giacomo et al., 2017; Pauselli, Verdolini, Santucci, Moretti, & Quartesan, 2015; Reich, Zanarini, & Fitzmaurice, 2012).

Previous studies comparing BD and BPD patients showed that BPD patients had significantly more difficulties in interpersonal relationships, endorsed negative and distressing beliefs about themselves and their relationships, and had dysfunctional maternal relationships as compared to BD patients (Bayes et al., 2015; Fletcher, Parker, Bayes, Paterson, & McClure, 2014; Nilsson et al., 2010). Our findings also indicate that relational difficulties with the self and others, such as disturbances in self representation, negative experiences in current relationships and insecure attachment, are evident in people with BPD features. Conflictive interpersonal relationships could distinguish BPD from BD (Massó Rodriguez et al., 2021). However, to be able to support this, these factors should also be studied in BD at-risk populations.

Relatively less evidence has accumulated about precursors related to the BPD development. The reason might be ascribed to the fact that the BPD phenotype is less clearly identified compared to the BD prodromal phase, although its underlying dimensions are evident in the reviews included in this study. This might be attributable to the short follow-up periods and not integrating contemporary methods for defining biological, psychological, and social precursor signs for the development of BPD (Chanen & Kaess, 2011). Staging models, like in BD or psychosis, could be utilised to help predict the course of prognosis with external validation through biomarkers (Hutsebaut & Aleva, 2021; Videler, Hutsebaut, Schulkens, Sobczak, & van Alphen, 2019). However, early stages of most of these symptoms are non-specific and overlap with other disorders (Berk et al., 2017).

Most of the precursors and vulnerability factors evident in the reviews were shared in both disorders, but some factors were either more evident in BD at-risk or BPD at-risk or they differed in phenomenological aspects. For example, BD at-risk patients had decreased need for sleep, hypersomnia, low social rhythm regularity and high energy whereas BPD patients had chronic nightmares and it was mediated by emotional and behavioural problems. BD at-risk patients showed ‘bipolar depression’ rather than unipolar depression, but in emerging BPD the course was unipolar depression. In BPD at-risk, there was risk of self-harm, but it was not stable after post-hospitalisation. As DSM criteria state that BPD traits are chronic and pervasive (American Psychiatric Association, 2013), the nature of BPD as a personality disorder thereby is doubtful. Further, previous research has observed similar frequency in self-harm in BD patients (Joyce, Light, Rowe, Cloninger, & Kennedy, 2010). Therefore, although self-harm is evident only in BPD studies, it does not distinguish these disorders diagnostically. Importantly, subjects at-risk for attempting suicide usually approach it through searching information and news regarding self-harm and suicidal behaviours on the Internet (Solano et al., 2016). Better insight and understanding of suicide and suicidal risk in these at-risk populations may ultimately help clinicians to adequately detect and prevent suicidal acts.

Whilst AI is transdiagnostic (Marwaha et al., 2016), it is also regarded as a shared feature in BD and BPD diagnosis. AI was evident in BD onset coexistent with baseline MDD, and it was defined as ‘having ups and downs’ whereas in BPD onset, it was part of negative affectivity, aggression, and impulsivity. Difficulties in relationships are a core BPD feature, manifested by idealisation and devaluation as well as by rejection sensitivity (Bayes et al., 2015; Gunderson, 2007). Considering the findings, they are in line with the previous cross-diagnostic studies. Saunders, Goodwin, and Rogers (2015) reported that patients with BPD had higher negative affect, impulsivity, aggression and reduced cooperative relationships. Likewise, Henry et al. (2001) suggested that BPD is not simply an attenuated subgroup of affective disorders and that it could be distinguished from BD on the basis of temperament and character. Additionally, the valence, frequency and nature of mood/affect regulation or mood swings is key to both BD and BPD, and likely especially as the conditions are developing (Marwaha et al., 2014). It was therefore surprising that this aspect of psychopathology has not been comprehensively assessed in people with at-risk conditions. Indeed, this is one way that the conditions could be distinguished. Advancing this field will require future comparative studies of affect/mood regulation in young people with emerging BD *v.* BPD. The time scale of the mood fluctuations can be a useful marker in clinical practice to differentiate BD and BPD in at-risk asymptomatic periods.

Childhood adversity was evident in both disorders (e.g. Palmier-Claus, Berry, Bucci, Mansell, and Varese, 2016; Ratheesh et al., 2017; Skabeikyte and Barkauskiene, 2021; Stepp et al., 2016; Winsper et al., 2016b). However, the evidence was much scarcer and sparser in BD studies. For example, apart from childhood sexual, physical, and verbal abuse and neglect, peer victimisation and abuse in romantic relationships were also evident in BPD. These findings are consistent with the previous literature stating that there is a higher likelihood of experiencing childhood adversity in BPD patients compared to BD (Afifi et al., 2011; Cotter, Kaess, & Yung, 2014). These adverse experiences might be the reason why BPD patients tend show higher aggressiveness and anger in mood shifts compared to BD patients.

In line with the recent research on the circadian rest-activity patterns in BD and BPD patients (McGowan, Goodwin, Bilderbeck, & Saunders, 2019), sleep disturbances and difficulty falling asleep were common to both disorders. In BPD studies, chronic nightmares were significantly predictive of the onset whereas in BD, participants had decreased need of sleep and it was part of hypomanic symptoms. Vöhringer et al. (2016) too indicated that decreased need for sleep was part of manic symptoms and were specific to BD and not to BPD patients.

Comorbid SUD, anxiety disorders, psychotic symptoms, ADHD were common to both disorder onsets. In BD studies comorbid generalised anxiety disorder and social phobia and ADHD with and without baseline comorbid conduct disorder predicted BD onset whereas in BPD, ADHD and OCD predicted BPD. Further, psychotic symptoms in BD at-risk studies were most often linked to affective states which is in line with previous research (Bassett, 2012). However, the nature of the psychotic symptoms in BPD at-risk studies were not evident. Temperamental dimensions were also evident in both. Higher levels of activity and poor psychosocial functioning were common to both, but in BD onset daydreaming, cyclothymia, and temperamental instability during MDD episodes were predictors of transition. Additionally, sensitivity, hyper alertness, excessive talk or talking too loudly, somatic complaints, and impaired role in school predicted conversion to BD. In BPD studies, on the contrary, higher levels of emotionality, low levels of sociability and shyness predicted BPD symptoms. These traits again might be attributable to the fact that BPD patients having more conflictive interpersonal relationships (MacKinnon & Pies, 2006).

Depression was also predictive of BD onset and later BPD symptoms although most of the studies pertained to BD at-risk reviews. In BD at-risk studies, depressive episodes or MDD, chronicity, severity, age at onset, psychomotor retardation and frequency of depression predicted BD transition. In emerging BPD studies, only early onset of depression was related to later BPD symptoms. In BD at-risk studies, there is also coexistence of atypical depressive symptoms that are considered to be ‘bipolar depression’ and distinct in phenomenology from unipolar depression such as pathological guilt, cyclothymia, mood lability, psychotic symptoms, and subthreshold hypomania (Berk et al., 2007, 2010). Detailed studies about depressive states in emerging BPD populations are urgently needed to be able to understand whether they can be distinguished based on the depressive symptomatology.

Family history of BD, although the results were inconsistent, was the prominent predictor of BD conversion compared to family history of depression or any affective disorders. For BPD, apart from maternal BPD symptoms (Winsper et al., 2016a), paternal SUD, family history of psychiatric hospitalisation, and maternal psychopathology were significantly associated with BPD. Consistent with the previous research the family history of BD might be a prominent distinguishing feature when comparing BD and BPD (Galione & Zimmerman, 2010; Mitchell, Goodwin, Johnson, & Hirschfeld, 2008). However, there is paucity of research pertaining to BPD. Further, despite existing family history of BD data might support the conclusion that BD is highly heritable and unrelated to BPD, no studies have examined the familial relationship of BPD traits and conversion to BD or vice versa.

Our review has strengths. We utilised systematic search procedures to reduce risk of bias and ensure comprehensive coverage of the current literature. Inter-rater reliability was consistent with no requirement for arbitration regarding inclusion of SRs and MAs. However, several limitations of the current findings here should be considered when interpreting the results. First, we only included relevant prospective studies from the SRs and excluded primary studies with any other designs. This inhibited synthesising the articles as a whole and reporting the pooled results from eligible MA's. Second, the evidence was limited by the data that included SRs and MAs provided and some relevant prospective studies were inevitably missed. Third, there were many non-systematic literature reviews including prospective studies that the reviews we included missed out. For example, Hartmann, Nelson, Ratheesh, Treen, & McGorry's (2018) scoping review, they provided additional evidence for family history of BD, subthreshold depression and hypomania, sleep disturbance, mood lability and later BD conversion. They also provided data for impulsivity and fun-seeking which was not investigated by the reviews included here. Fourth, we were not able to conduct an MA due to the substantial methodological and clinical heterogeneity with respect to cohort characteristics such as study design and sample size among primary studies included in the reviews. Further, not being able to pool the data precluded definitively clarifying the timing and duration of the precursors, notwithstanding the heterogeneity in at-risk populations (Radua et al., 2018). Fifth, because of the short follow-up periods and small number of follow-up assessments in most of the included prospective studies, the validity and utility of these factors for predicting an early prodrome of BD and BPD remains unknown. If we do not know the actual starting point of the onset of the disorder, these and any other identified factors may indicate relapse or maintenance of the disorder (Stepp & Lazarus, 2017). Sixth, none of the reviews discussed the sensitivity, specificity, and predictive value of reported precursors, important aspects that may enable better assessment of clinical utility. Therefore, again a cautious interpretation of the findings as to their generalisability is necessary. Seventh, none of the studies mentioned sub-score analyses for the examined antecedents making it challenging to compare how the two disorders presented different patterns of the shared features. Eight, the majority of the BD at-risk reviews examined youth at genetic high risk. However, most genetically high-risk individuals do not develop BD. A combination of genetic and clinical risk factors is required to optimally predict conversion to BD (Keramatian et al., 2021). Nineth, despite our comprehensive search, we identified relatively very few studies pertaining to the BPD onset.

In conclusion, although the findings of this review may lead to support the view of BD and BPD as two distinct disorders, there is scant evidence from existing studies to either indicate that BD and BPD are separate nosological entities or that BPD should be considered as an extension of BD disorders. In clinical practice, these differences can be subtle, especially between BPD and BD-II (Massó Rodriguez et al., 2021).

Whilst the comparative literature is in its infancy there are several implications from this meta-review. On an etio-pathological level, our findings corroborate the notion that there is a prodromal stage in BD and BPD. There are overlapping risk factors in the young people with at-risk BD and BPD, these being family history of psychopathology, AI, ADHD, anxiety disorders, depression, sleep disturbances, substance abuse, psychotic symptoms, suicidality, childhood adversity and temperament. However, there are risk factors specific to the at-risk BD and BPD states. Gender, differences in the white matter, changes in the amygdala, neural reward circuit dysfunctions, DBD, subsyndromal hypomania, cyclothymia or bipolar NOS, frequency and loading of affective symptoms, and antidepressant use were evident only in people with at-risk BD. Parenting behaviour/style, parent-child relationship quality, maternal characteristics, attachment, impulsivity, experiential avoidance, disturbances in self representation, dissociation, comorbid oppositional defiant disorder, comorbid conduct disorder, somatisation, general psychosocial functioning, and social and physical aggression in childhood were evident only in at-risk BPD. These factors could form the basis of initial prediction modelling approaches which could improve clinical staging and clinical interventions. Clinicians should be aware of the high degree of comorbid psychopathology in young people developing BD and BPD and should consider both conditions in young people presenting with one. From a transdiagnostic perspective, the current review may provide a benchmark for comparing the magnitude of association of these factors with other mental health disorders. The results can also substantially advance our ability to prognosticate the onset of BD and BPD in populations at-risk, who ultimately may benefit from preventative interventions.

To be able to reliably identify target populations with greater specificity, future research is required to increase our understanding of the development of BD and BPD onset and their complex interplay by conducting prospective studies which concurrently examine multiple measures including biological, environmental, psychosocial, and clinical factors in BD and BPD at-risk populations. Systematic longitudinal studies investigating genetically and clinically high-risk youths in a structured multifactorial approach can help us understand whether both these disorders belong to the affective spectrum or not as well as their development over time (e.g. Brietzke et al., 2012). Greater predictive validity could be provided by future research identifying potential BD and BPD biomarkers whilst charting these along the illness trajectory. It is also important that future research studies use consistent recruitment criteria to ensure that findings are comparable and generalisable to other studies as far as is practicable (Malhi et al., 2017).

Large, multilevel data sets will enable deep phenotyping and distinguish pathophysiological pathways (Phillips & Kendler, 2021). For example, remote monitoring can complement symptom monitoring and capture signals more representative of the underlying pathophysiology of BD and BPD (Gillett et al., 2021; Gillett & Saunders, 2019). One of the ways to conduct remote monitoring is Experience Sampling Methodology (ESM). The temporal pattern in mood may be captured by ESM (Larson & Csikszentmihalyi, 2014). Researchers have widely used ESM to assess the temporal patterns of regulations of mood/affect in individuals with mood disorders as the method is more suited to capturing momentary temporal fluctuations in affect (e.g. Dubad, Elahi, and Marwaha, 2021; Merikangas et al., 2019; Schwartz, Schultz, Reider, and Saunders, 2016; Tsanas et al., 2016). Further, some types of ESM are not subject to recall biases as the studies do not rely on retrospective memory recall of events between assessments (Myin-Germeys et al., 2009). The data gained through could also identify new behavioural biomarkers which may lead to the identification of novel phenotypes in these disorders (Gillett & Saunders, 2019). Additionally, identifying common criteria such as AI is easy but focusing on differential symptoms is a complex task (Massó Rodriguez et al., 2021). ESM could also be useful to be able to achieve this. These prospective studies may also help identifying a validated BPD prodrome criteria, despite previous resistance to the diagnosis of BPD in adolescents due to the fears of stigmatisation (Chanen, 2015; Laurenssen, Hutsebaut, Feenstra, Van Busschbach, & Luyten, 2013; Stepp & Lazarus, 2017).

Making an accurate diagnosis of BD and BPD is further complicated by comorbidity with various other conditions such as ADHD and unipolar depression (Asherson et al., 2014; Mneimne, Fleeson, Arnold, & Furr, 2018). ADHD has been reported to coexist in around 20% of adult patients with BPD or BD (Asherson et al., 2014; Philipsen et al., 2009; Skirrow, Hosang, Farmer, & Asherson, 2012), while rates of co-occurrence between BPD and current major depressive disorder MDD or BD range from as low as 4% to as high as 48% (Mneimne et al., 2018). Since deficits in affect regulation such as AI are also strongly linked to the hyperactive/impulsivity symptoms of ADHD (Skirrow & Asherson, 2013) and unipolar depression (Balbuena, Bowen, Baetz, & Marwaha, 2016), it is imperative to include these groups too for comparison to better understand the symptom profiles between at-risk BD and BPD.

## Supplementary material

For supplementary material accompanying this paper visit https://doi.org/10.1017/S0033291722003002. click here to view supplementary material
