# Supplementary material for: Understanding the development of bipolar disorder and borderline personality disorder in young people: a meta-review of systematic reviews
Source: Psychol Med. 2022 Sep 30;52(16):3769–82. doi: 10.1017/S0033291722003002 (PMC9816307; doi:10.1017/S0033291722003002)
Supplement: Supplementary file 1 [file S0033291722003002sup001.docx]

**Supplementary 1.** The Preferred Reporting Items for Systematic Reviews and Meta-Analyses Checklist

**Supplementary 2.** Search Strategy

**Supplementary 3.** Included primary studies contained within systematic reviews and meta-analyses.

**Supplementary 4.** Vulnerability Factors

**Table S1.** Characteristics of systematic reviews and meta-analyses examining the developmental pathways and onset of Bipolar Disorder

**Table S2.** Characteristics of systematic reviews and meta-analyses examining the developmental pathways and onset of Borderline Personality Disorder

**Table S3** Evidence across systematic reviews and meta-analyses of factors for Bipolar Disorder outcome

**Table S4** Evidence across systematic reviews and meta-analyses of factors for Borderline Personality outcome

**Table S5.** Excluded studies after full-text review with reasons

**Table S6.** Methodological quality of included systematic reviews and meta-analyses based on AMSTAR tool for Bipolar Disorder

**Table S7.** Methodological quality of included systematic reviews and meta-analyses based on AMSTAR tool for Borderline Personality Disorder

**Table S8.** Citation matrix for Bipolar Disorder

**Table S9.** Citation matrix for Borderline Personality Disorder

**Figure S1.** Pairwise CCA for reviews on BD

**Figure S2.** Pairwise CCA for reviews on BPD

**Supplementary 1.** The Preferred Reporting Items for Systematic Reviews and Meta-Analyses Checklist

| **Section and Topic** | **Item #** | **Checklist item** | **Location where item is reported** |
| --- | --- | --- | --- |
| **TITLE** | | |  |
| Title | 1 | Identify the report as a systematic review. | 1 |
| **ABSTRACT** | | |  |
| Abstract | 2 | See the PRISMA 2020 for Abstracts checklist. | 2 |
| **INTRODUCTION** | | |  |
| Rationale | 3 | Describe the rationale for the review in the context of existing knowledge. | 3 |
| Objectives | 4 | Provide an explicit statement of the objective(s) or question(s) the review addresses. | 4 |
| **METHODS** | | |  |
| Eligibility criteria | 5 | Specify the inclusion and exclusion criteria for the review and how studies were grouped for the syntheses. | 4 |
| Information sources | 6 | Specify all databases, registers, websites, organisations, reference lists and other sources searched or consulted to identify studies. Specify the date when each source was last searched or consulted. | 4 |
| Search strategy | 7 | Present the full search strategies for all databases, registers and websites, including any filters and limits used. | 4 |
| Selection process | 8 | Specify the methods used to decide whether a study met the inclusion criteria of the review, including how many reviewers screened each record and each report retrieved, whether they worked independently, and if applicable, details of automation tools used in the process. | 5 |
| Data collection process | 9 | Specify the methods used to collect data from reports, including how many reviewers collected data from each report, whether they worked independently, any processes for obtaining or confirming data from study investigators, and if applicable, details of automation tools used in the process. | 5 |
| Data items | 10a | List and define all outcomes for which data were sought. Specify whether all results that were compatible with each outcome domain in each study were sought (e.g. for all measures, time points, analyses), and if not, the methods used to decide which results to collect. | - |
|  | 10b | List and define all other variables for which data were sought (e.g. participant and intervention characteristics, funding sources). Describe any assumptions made about any missing or unclear information. | 5 & Supplement |
| Study risk of bias assessment | 11 | Specify the methods used to assess risk of bias in the included studies, including details of the tool(s) used, how many reviewers assessed each study and whether they worked independently, and if applicable, details of automation tools used in the process. | 5 |
| Effect measures | 12 | Specify for each outcome the effect measure(s) (e.g. risk ratio, mean difference) used in the synthesis or presentation of results. | - |
| Synthesis methods | 13a | Describe the processes used to decide which studies were eligible for each synthesis (e.g. tabulating the study intervention characteristics and comparing against the planned groups for each synthesis (item #5)). | 5 |
|  | 13b | Describe any methods required to prepare the data for presentation or synthesis, such as handling of missing summary statistics, or data conversions. | - |
|  | 13c | Describe any methods used to tabulate or visually display results of individual studies and syntheses. | 5 |
|  | 13d | Describe any methods used to synthesize results and provide a rationale for the choice(s). If meta-analysis was performed, describe the model(s), method(s) to identify the presence and extent of statistical heterogeneity, and software package(s) used. | 5 |
|  | 13e | Describe any methods used to explore possible causes of heterogeneity among study results (e.g. subgroup analysis, meta-regression). | - |
|  | 13f | Describe any sensitivity analyses conducted to assess robustness of the synthesized results. | - |
| Reporting bias assessment | 14 | Describe any methods used to assess risk of bias due to missing results in a synthesis (arising from reporting biases). | 5 & Supplement |
| Certainty assessment | 15 | Describe any methods used to assess certainty (or confidence) in the body of evidence for an outcome. | - |
| **RESULTS** | | |  |
| Study selection | 16a | Describe the results of the search and selection process, from the number of records identified in the search to the number of studies included in the review, ideally using a flow diagram. | 5-6 |
|  | 16b | Cite studies that might appear to meet the inclusion criteria, but which were excluded, and explain why they were excluded. | 6 |
| Study characteristics | 17 | Cite each included study and present its characteristics. | 6 |
| Risk of bias in studies | 18 | Present assessments of risk of bias for each included study. | 6 |
| Results of individual studies | 19 | For all outcomes, present, for each study: (a) summary statistics for each group (where appropriate) and (b) an effect estimate and its precision (e.g. confidence/credible interval), ideally using structured tables or plots. | - |
| Results of syntheses | 20a | For each synthesis, briefly summarise the characteristics and risk of bias among contributing studies. | 6 |
|  | 20b | Present results of all statistical syntheses conducted. If meta-analysis was done, present for each the summary estimate and its precision (e.g. confidence/credible interval) and measures of statistical heterogeneity. If comparing groups, describe the direction of the effect. | - |
|  | 20c | Present results of all investigations of possible causes of heterogeneity among study results. | - |
|  | 20d | Present results of all sensitivity analyses conducted to assess the robustness of the synthesized results. | - |
| Reporting biases | 21 | Present assessments of risk of bias due to missing results (arising from reporting biases) for each synthesis assessed. | - |
| Certainty of evidence | 22 | Present assessments of certainty (or confidence) in the body of evidence for each outcome assessed. | - |
| **DISCUSSION** | | |  |
| Discussion | 23a | Provide a general interpretation of the results in the context of other evidence. | 9-12 |
|  | 23b | Discuss any limitations of the evidence included in the review. | 12 |
|  | 23c | Discuss any limitations of the review processes used. | 12 |
|  | 23d | Discuss implications of the results for practice, policy, and future research. | 12 |
| **OTHER INFORMATION** | | |  |
| Registration and protocol | 24a | Provide registration information for the review, including register name and registration number, or state that the review was not registered. | 4 |
|  | 24b | Indicate where the review protocol can be accessed, or state that a protocol was not prepared. | 4 |
|  | 24c | Describe and explain any amendments to information provided at registration or in the protocol. | - |
| Support | 25 | Describe sources of financial or non-financial support for the review, and the role of the funders or sponsors in the review. | 13 |
| Competing interests | 26 | Declare any competing interests of review authors. | 13 |
| Availability of data, code and other materials | 27 | Report which of the following are publicly available and where they can be found: template data collection forms; data extracted from included studies; data used for all analyses; analytic code; any other materials used in the review. | Supplement |

**Supplementary 3.** Included primary studies contained within systematic reviews and meta-analyses.

Acuff, H. E., Versace, A., Bertocci, M. A., Ladouceur, C. D., Hanford, L. C., Manelis, A., ... Phillips, M. L. (2019). Baseline and follow-up activity and functional connectivity in reward neural circuitries in offspring at risk for bipolar disorder. *Neuropsychopharmacology*, *44*(9), 1570-1578.

Akiskal, H. S., Djenderedjian, A. H., Rosenthal, R. H., & Khani, M. K. (1977). Cyclothymic disorder: Validating criteria for inclusion in the bipolar affective group. *The American Journal of Psychiatry*.

Akiskal, H. S., Bitar, A. H., Puzantian, V. R., Rosenthal, T. L., & Walker, P. W. (1978). The nosological status of neurotic depression: a prospective three-to four-year follow-up examination in light of the primary-secondary and unipolar-bipolar dichotomies. *Archives of General Psychiatry*, *35*(6), 756-766.

Akiskal, H. S., Walker, P., Puzantian, V. R., King, D., Rosenthal, T. L., & Dranon, M. (1983). Bipolar outcome in the course of depressive illness: phenomenologic, familial, and pharmacologic predictors. *Journal of affective disorders*, *5*(2), 115-128.

Akiskal, H. S., Downs, J., Jordan, P., Watson, S., Daugherty, D., & Pruitt, D. B. (1985). Affective disorders in referred children and younger siblings of manic-depressives: mode of onset and prospective course. *Archives of General Psychiatry*, *42*(10), 996-1003.

Akiskal, H. S., Maser, J. D., Zeller, P. J., Endicott, J., Coryell, W., Keller, M., ... Goodwin, F. (1995). Switching from'unipolar'to bipolar II: an 11-year prospective study of clinical and temperamental predictors in 559 patients. *Archives of General Psychiatry*, *52*(2), 114-123.

Alloy, L. B., Urošević, S., Abramson, L. Y., Jager-Hyman, S., Nusslock, R., Whitehouse, W. G., & Hogan, M. (2012). Progression along the bipolar spectrum: a longitudinal study of predictors of conversion from bipolar spectrum conditions to bipolar I and II disorders. *Journal of Abnormal Psychology*, *121*(1), 16.

Alloy, L. B., Boland, E. M., Ng, T. H., Whitehouse, W. G., & Abramson, L. Y. (2015). Low social rhythm regularity predicts first onset of bipolar spectrum disorders among at-risk individuals with reward hypersensitivity. *Journal of Abnormal Psychology*, *124*(4), 944.

Anderson, C. A., & Hammen, C. L. (1993). Psychosocial outcomes of children of unipolar depressed, bipolar, medically ill, and normal women: a longitudinal study. *Journal of Consulting and Clinical Psychology*, *61*(3), 448.

Angst, J., Gamma, A., & Endrass, J. (2003). Risk factors for the bipolar and depression spectra. *Acta Psychiatrica Scandinavica*, *108*, 15-19.

Angst, J., Sellaro, R., Stassen, H. H., & Gamma, A. (2005). Diagnostic conversion from depression to bipolar disorders: results of a long-term prospective study of hospital admissions. *Journal of Affective Disorders*, *84*(2-3), 149-157.

Arnold, L. E., Van Meter, A. R., Fristad, M. A., Youngstrom, E. A., Birmaher, B. B., Findling, R. L., ... Black, S. R. (2020). Development of bipolar disorder and other comorbidity among youth with attention‐deficit/hyperactivity disorder. *Journal of Child Psychology and Psychiatry*, *61*(2), 175-181.

Axelson, D. A., Birmaher, B., Strober, M. A., Goldstein, B. I., Ha, W., Gill, M. K., ... Keller, M. B. (2011). Course of subthreshold bipolar disorder in youth: diagnostic progression from bipolar disorder not otherwise specified. *Journal of the American Academy of Child & Adolescent Psychiatry*, *50*(10), 1001-1016.

Axelson, D., Goldstein, B., Goldstein, T., Monk, K., Yu, H., Hickey, M. B., ... Birmaher, B. (2015). Diagnostic precursors to bipolar disorder in offspring of parents with bipolar disorder: a longitudinal study. *American Journal of Psychiatry*, *172*(7), 638-646.

Bauwens, F., Pardoen, D., Staner, L., Dramaix, M., & Mendlewicz, J. (1998). Social adjustment and the course of affective illness: A one‐year controlled longitudinal study involving bipolar and unipolar outpatients. *Depression and Anxiety*, *8*(2), 50-57.

Barnow, S., Aldinger, M., Arens, E. A., Ulrich, I., Spitzer, C., Grabe, H. J., & Stopsack, M. (2013). Maternal transmission of borderline personality disorder symptoms in the community-based Greifswald Family Study. *Journal of Personality Disorders*, *27*(6), 806-819.

Bechdolf, A., Ratheesh, A., Cotton, S. M., Nelson, B., Chanen, A. M., Betts, J., ... McGorry, P. D. (2014). The predictive validity of bipolar at‐risk (prodromal) criteria in help‐seeking adolescents and young adults: a prospective study. *Bipolar Disorders*, *16*(5), 493-504.

Beesdo, K., Höfler, M., Leibenluft, E., Lieb, R., Bauer, M., & Pfennig, A. (2009). Mood episodes and mood disorders: patterns of incidence and conversion in the first three decades of life. *Bipolar Disorders*, *11*(6), 637-649.

Belsky, D. W., Caspi, A., Arseneault, L., Bleidorn, W., Fonagy, P., Goodman, M., ... Moffitt, T. E. (2012). Etiological features of borderline personality related characteristics in a birth cohort of 12-year-old children. *Development and Psychopathology*, *24*(1), 251-265.

Benvenuti, A., Rucci, P., Miniati, M., Papasogli, A., Fagiolini, A., Cassano, G. B., ... Frank, E. (2008). Treatment‐emergent mania/hypomania in unipolar patients. *Bipolar Disorders*, *10*(6), 726-732.

Bertocci, M. A., Hanford, L., Manelis, A., Iyengar, S., Youngstrom, E. A., Gill, M. K., ... Phillips, M. L. (2019). Clinical, cortical thickness and neural activity predictors of future affective lability in youth at risk for bipolar disorder: initial discovery and independent sample replication. *Molecular Psychiatry*, *24*(12), 1856-1867.

Bezirganian, S., Cohen, P., & Brook, J. S. (1993). The impact of mother-child interaction on the development of borderline personality disorder. *The American Journal of Psychiatry*.

Biederman, J., Faraone, S., Milberger, S., Guite, J., Mick, E., Chen, L., ... Perrin, J. (1996). A prospective 4-year follow-up study of attention-deficit hyperactivity and related disorders. *Archives of General Psychiatry*, *53*(5), 437-446.

Biederman, J., Petty, C. R., Dolan, C., Hughes, S., Mick, E., Monuteaux, M. C., & Faraone, S. V. (2008). The long-term longitudinal course of oppositional defiant disorder and conduct disorder in ADHD boys: findings from a controlled 10-year prospective longitudinal follow-up study. *Psychological Medicine*, *38*(7), 1027-1036.

Biederman, J., Petty, C. R., Byrne, D., Wong, P., Wozniak, J., & Faraone, S. V. (2009). Risk for switch from unipolar to bipolar disorder in youth with ADHD: a long term prospective controlled study. *Journal of Affective Disorders*, *119*(1-3), 16-21.

Biederman, J., Wozniak, J., Tarko, L., Serra, G., Hernandez, M., McDermott, K., ... Faraone, S. V. (2014). Re-examining the risk for switch from unipolar to bipolar major depressive disorder in youth with ADHD: a long term prospective longitudinal controlled study. *Journal of Affective Disorders*, *152*, 347-351.

Birmaher, B., Merranko, J., Hafeman, D., Goldstein, B. I., Diler, R., Levenson, J. C., ... Goldstein, T. (2021). A longitudinal study of psychiatric disorders in offspring of parents with bipolar disorder from preschool to adolescence. *Journal of the American Academy of Child & Adolescent Psychiatry*, *60*(11), 1419-1429.

Birmaher, B., Merranko, J. A., Goldstein, T. R., Gill, M. K., Goldstein, B. I., Hower, H., ... Keller, M. B. (2018). A risk calculator to predict the individual risk of conversion from subthreshold bipolar symptoms to bipolar disorder I or II in youth. *Journal of the American Academy of Child & Adolescent Psychiatry*, *57*(10), 755-763.

Bitter, S. M., Mills, N. P., Adler, C. M., Strakowski, S. M., & DelBello, M. P. (2011). Progression of amygdala volumetric abnormalities in adolescents after their first manic episode. *Journal of the American Academy of Child & Adolescent Psychiatry*, *50*(10), 1017-1026.

Boschloo, L., Spijker, A. T., Hoencamp, E., Kupka, R., Nolen, W. A., Schoevers, R. A., & Penninx, B. W. (2014). Predictors of the onset of manic symptoms and a (hypo) manic episode in patients with major depressive disorder. *PLoS One*, *9*(9), e106871.

Bornovalova, M. A., Hicks, B. M., Iacono, W. G., & McGue, M. (2009). Stability, change, and heritability of borderline personality disorder traits from adolescence to adulthood: A longitudinal twin study. *Development and Psychopathology*, *21*(4), 1335-1353.

Bornovalova, M. A., Hicks, B. M., Iacono, W. G., & McGue, M. (2013). Longitudinal twin study of borderline personality disorder traits and substance use in adolescence: developmental change, reciprocal effects, and genetic and environmental influences. *Personality Disorders: Theory, Research, and Treatment*, *4*(1), 23.

Bornovalova, M. A., Huibregtse, B. M., Hicks, B. M., Keyes, M., McGue, M., & Iacono, W. (2013). Tests of a direct effect of childhood abuse on adult borderline personality disorder traits: a longitudinal discordant twin design. *Journal of Abnormal Psychology*, *122*(1), 180.

Bornovalova, M. A., Verhulst, B., Webber, T., McGue, M., Iacono, W. G., & Hicks, B. M. (2018). Genetic and environmental influences on the codevelopment among borderline personality disorder traits, major depression symptoms, and substance use disorder symptoms from adolescence to young adulthood. *Development and Psychopathology*, *30*(1), 49-65.

Bromet, E. J., Kotov, R., Fochtmann, L. J., Carlson, G. A., Tanenberg-Karant, M., Ruggero, C., & Chang, S. W. (2011). Diagnostic shifts during the decade following first admission for psychosis. *American Journal of Psychiatry*, *168*(11), 1186-1194.

Bukh, J. D., Andersen, P. K., & Kessing, L. V. (2016). Personality and the long-term outcome of first-episode depression: a prospective 5-year follow-up study. *The Journal of Clinical Psychiatry*, *77*(6), 14149.

Bukh, J. D., Andersen, P. K., & Kessing, L. V. (2016). Rates and predictors of remission, recurrence and conversion to bipolar disorder after the first lifetime episode of depression–a prospective 5-year follow-up study. *Psychological Medicine*, *46*(6), 1151-1161.

Burke, J. D., & Stepp, S. D. (2012). Adolescent disruptive behavior and borderline personality disorder symptoms in young adult men. *Journal of Abnormal Child Psychology*, *40*(1), 35-44.

Carlson, E. A., Egeland, B., & Sroufe, L. A. (2009). A prospective investigation of the development of borderline personality symptoms. *Development and Psychopathology*, *21*(4), 1311-1334.

Cassano, P., Lattanzi, L., Soldani, F., Navari, S., Battistini, G., Gemignani, A., & Cassano, G. B. (2004). Pramipexole in treatment‐resistant depression: An extended follow‐up. *Depression and Anxiety*, *20*(3), 131-138.

Castro‐Fornieles, J., Baeza, I., de la Serna, E., Gonzalez‐Pinto, A., Parellada, M., Graell, M., ... Arango, C. (2011). Two‐year diagnostic stability in early‐onset first‐episode psychosis. *Journal of Child Psychology and Psychiatry*, *52*(10), 1089-1098.

Chung, K. H., Li, C. Y., Kuo, S. Y., Sithole, T., Liu, W. W., & Chung, M. H. (2015). Risk of psychiatric disorders in patients with chronic insomnia and sedative-hypnotic prescription: a nationwide population-based follow-up study. *Journal of Clinical Sleep Medicine*, *11*(5), 543-551.

Cohen, P., Chen, H., Gordon, K., Johnson, J., Brook, J., & Kasen, S. (2008). Socioeconomic background and the developmental course of schizotypal and borderline personality disorder symptoms. *Development and Psychopathology*, *20*(2), 633-650.

Conway, C. C., Hammen, C., & Brennan, P. A. (2015). Adolescent Precursors of Adult Borderline Personality Pathology in a High Risk Community Sample. *Journal of Personality Disorders*, *29*(3), 316.

Coryell, W., Andreasen, N. C., Endicott, J., & Keller, M. (1987). The significance of past mania or hypomania in the course and outcome of major depression. *The American Journal of Psychiatry*.

Coryell, W., Endicott, J., Maser, J. D., Keller, M. B., Leon, A. C., & Akiskal, H. S. (1995). Long-term stability of polarity distinctions in the affective disorders. *The American Journal of Psychiatry*.

Curry, J., Silva, S., Rohde, P., Ginsburg, G., Kratochvil, C., Simons, A., ... March, J. (2011). Recovery and recurrence following treatment for adolescent major depression. *Archives of General Psychiatry*, *68*(3), 263-269.

Crawford, T. N., Cohen, P. R., Chen, H., Anglin, D. M., & Ehrensaft, M. (2009). Early maternal separation and the trajectory of borderline personality disorder symptoms. *Development and Psychopathology*, *21*(3), 1013-1030.

Crick, N. R., Murray-Close, D., & Woods, K. (2005). Borderline personality features in childhood: A short-term longitudinal study. *Development and Psychopathology*, *17*(4), 1051-1070.

DelBello, M. P., Carlson, G. A., Tohen, M., Bromet, E. J., Schwiers, M., & Strakowski, S. M. (2003). Rates and predictors of developing a manic or hypomanic episode 1 to 2 years following a first hospitalization for major depression with psychotic features. *Journal of Child and Adolescent Psychopharmacology*, *13*(2), 173-185.

DeGeorge, D. P., Walsh, M. A., Barrantes-Vidal, N., & Kwapil, T. R. (2014). A three-year longitudinal study of affective temperaments and risk for psychopathology. *Journal of Affective Disorders*, *164*, 94-100.

Dixon-Gordon, K. L., Whalen, D. J., Scott, L. N., Cummins, N. D., & Stepp, S. D. (2016). The main and interactive effects of maternal interpersonal emotion regulation and negative affect on adolescent girls’ borderline personality disorder symptoms. *Cognitive Therapy and Research*, *40*(3), 381-393.

Doucette, S., Horrocks, J., Grof, P., Keown-Stoneman, C., & Duffy, A. (2013). Attachment and temperament profiles among the offspring of a parent with bipolar disorder. *Journal of Affective Disorders*, *150*(2), 522-526.

Duffy, A., Alda, M., Crawford, L., Milin, R., & Grof, P. (2007). The early manifestations of bipolar disorder: a longitudinal prospective study of the offspring of bipolar parents. *Bipolar Disorders*, *9*(8), 828-838.

Duffy, A., Alda, M., Hajek, T., & Grof, P. (2009). Early course of bipolar disorder in high-risk offspring: prospective study. *The British Journal of Psychiatry*, *195*(5), 457-458.

Duffy, A., Alda, M., Hajek, T., Sherry, S. B., & Grof, P. (2010). Early stages in the development of bipolar disorder. *Journal of Affective Disorders*, *121*(1-2), 127-135.

Duffy, A., Horrocks, J., Milin, R., Doucette, S., Persson, G., & Grof, P. (2012). Adolescent substance use disorder during the early stages of bipolar disorder: a prospective high-risk study. *Journal of Affective Disorders*, *142*(1-3), 57-64.

Duffy, A., Horrocks, J., Doucette, S., Keown-Stoneman, C., McCloskey, S., & Grof, P. (2014). The developmental trajectory of bipolar disorder. *The British Journal of Psychiatry*, *204*(2), 122-128.

Duffy, A., Goodday, S., Keown-Stoneman, C., & Grof, P. (2019). The emergent course of bipolar disorder: observations over two decades from the Canadian high-risk offspring cohort. *American Journal of Psychiatry*, *176*(9), 720-729.

Dunn, V., & Goodyer, I. M. (2006). Longitudinal investigation into childhood-and adolescence-onset depression: psychiatric outcome in early adulthood. *The British Journal of Psychiatry*, *188*(3), 216-222.

Egeland, J. A., Shaw, J. A., Endicott, J., Pauls, D. L., Allen, C. R., Hostetter, A. M., & Sussex, J. N. (2003). Prospective study of prodromal features for bipolarity in well Amish children. *Journal of the American Academy of Child & Adolescent Psychiatry*, *42*(7), 786-796.

Egeland, J. A., Endicott, J., Hostetter, A. M., Allen, C. R., Pauls, D. L., & Shaw, J. A. (2012). A 16-year prospective study of prodromal features prior to BPI onset in well Amish children. *Journal of Affective Disorders*, *142*(1-3), 186-192.

Ehrenreich, S. E., Beron, K. J., & Underwood, M. K. (2016). Social and physical aggression trajectories from childhood through late adolescence: Predictors of psychosocial maladjustment at age 18. *Developmental Psychology*, *52*(3), 457.

Fiedorowicz, J. G., Endicott, J., Leon, A. C., Solomon, D. A., Keller, M. B., & Coryell, W. H. (2011). Subthreshold hypomanic symptoms in progression from unipolar major depression to bipolar disorder. *American Journal of Psychiatry*, *168*(1), 40-48.

Fiedorowicz, J. G., Endicott, J., Solomon, D. A., Keller, M. B., & Coryell, W. H. (2012). Course of illness following prospectively observed mania or hypomania in individuals presenting with unipolar depression. *Bipolar Disorders*, *14*(6), 664-671.

Findling, R. L., Jo, B., Frazier, T. W., Youngstrom, E. A., Demeter, C. A., Fristad, M. A., ... Horwitz, S. M. (2013). The 24‐month course of manic symptoms in children. *Bipolar Disorders*, *15*(6), 669-679.

Frankland, A., Roberts, G., Holmes-Preston, E., Perich, T., Levy, F., Lenroot, R., ... Mitchell, P. B. (2018). Clinical predictors of conversion to bipolar disorder in a prospective longitudinal familial high-risk sample: focus on depressive features. *Psychological Medicine*, *48*(10), 1713-1721.

Fusar-Poli, P., De Micheli, A., Rocchetti, M., Cappucciati, M., Ramella-Cravaro, V., Rutigliano, G., ... Falkenberg, I. (2018). Semistructured interview for bipolar at risk states (SIBARS). *Psychiatry Research*, *264*, 302-309.

Furukawa, T. A., Konno, W., Morinobu, S., Harai, H., Kitamura, T., & Takahashi, K. (2000). Course and outcome of depressive episodes: comparison between bipolar, unipolar and subthreshold depression. *Psychiatry Research*, *96*(3), 211-220.

Gan, Z., Diao, F., Wei, Q., Wu, X., Cheng, M., Guan, N., ... & Zhang, J. (2011). A predictive model for diagnosing bipolar disorder based on the clinical characteristics of major depressive episodes in Chinese population. *Journal of Affective Disorders*, *134*(1-3), 119-125.

Ganzola, R., Nickson, T., Bastin, M. E., Giles, S., Macdonald, A., Sussmann, J., ... Duchesne, S. (2017). Longitudinal differences in white matter integrity in youth at high familial risk for bipolar disorder. *Bipolar Disorders*, *19*(3), 158-167.

Garber, J., Kriss, M. R., Koch, M., & Lindholm, L. (1988). Recurrent depression in adolescents: A follow-up study. *Journal of the American Academy of Child & Adolescent Psychiatry*, *27*(1), 49-54.

Geller, B., Fox, L. W., & Clark, K. A. (1994). Rate and predictors of prepubertal bipolarity during follow-up of 6-to 12-year-old depressed children. *Journal of the American Academy of Child & Adolescent Psychiatry*, *33*(4), 461-468.

Geller, B., Zimerman, B., Williams, M., Bolhofner, K., & Craney, J. L. (2001). Bipolar disorder at prospective follow-up of adults who had prepubertal major depressive disorder. *American Journal of Psychiatry*, *158*(1), 125-127.

Gilman, S. E., Ni, M. Y., Dunn, E. C., Breslau, J., McLaughlin, K. A., Smoller, J. W., & Perlis, R. H. (2015). Contributions of the social environment to first-onset and recurrent mania. *Molecular Psychiatry*, *20*(3), 329-336.

Gilman, S. E., Dupuy, J. M., & Perlis, R. H. (2012). Risks for the transition from major depressive disorder to bipolar disorder in the National Epidemiologic Survey on Alcohol and Related Conditions. *The Journal of Clinical Psychiatry*, *73*(6), 18480.

Gogtay, N., Ordonez, A., Herman, D. H., Hayashi, K. M., Greenstein, D., Vaituzis, C., ... Rapoport, J. L. (2007). Dynamic mapping of cortical development before and after the onset of pediatric bipolar illness. *Journal of Child Psychology and Psychiatry*, *48*(9), 852-862.

Goldberg, J. F., Harrow, M., & Grossman, L. S. (1995). Recurrent affective syndromes in bipolar and unipolar mood disorders at follow-up. *The British Journal of Psychiatry*, *166*(3), 382-385.

Goldberg, J. F., Harrow, M., & Whiteside, J. E. (2001). Risk for bipolar illness in patients initially hospitalized for unipolar depression. *American Journal of Psychiatry*, *158*(8), 1265-1270.

Greenfield, B., Henry, M., Lis, E., Slatkoff, J., Guilé, J. M., Dougherty, G., ... de Castro, F. (2015). Correlates, stability and predictors of borderline personality disorder among previously suicidal youth. *European Child & Adolescent Psychiatry*, *24*(4), 397-406.

Hallquist, M. N., Hipwell, A. E., & Stepp, S. D. (2015). Poor self-control and harsh punishment in childhood prospectively predict borderline personality symptoms in adolescent girls. *Journal of Abnormal Psychology*, *124*(3), 549.

Halperin, J. M., Rucklidge, J. J., Powers, R. L., Miller, C. J., & Newcorn, J. H. (2011). Childhood CBCL bipolar profile and adolescent/young adult personality disorders: A 9-year follow-up. *Journal of Affective Disorders*, *130*(1-2), 155-161.

Haltigan, J. D., & Vaillancourt, T. (2016). Identifying trajectories of borderline personality features in adolescence: antecedent and interactive risk factors. *The Canadian Journal of Psychiatry*, *61*(3), 166-175.

Hafeman, D. M., Merranko, J., Axelson, D., Goldstein, B. I., Goldstein, T., Monk, K., ... & Birmaher, B. (2016). Toward the definition of a bipolar prodrome: dimensional predictors of bipolar spectrum disorders in at-risk youths. *American Journal of Psychiatry*, *173*(7), 695-704.

Hafeman, D. M., Merranko, J., Goldstein, T. R., Axelson, D., Goldstein, B. I., Monk, K., ... & Birmaher, B. (2017). Assessment of a person-level risk calculator to predict new-onset bipolar spectrum disorder in youth at familial risk. *JAMA Psychiatry*, *74*(8), 841-847.

Hammen, C., Burge, D., Burney, E., & Adrian, C. (1990). Longitudinal study of diagnoses in children of women with unipolar and bipolar affective disorder. *Archives of General Psychiatry*, *47*(12), 1112-1117.

Hammen, C., Bower, J. E., & Cole, S. W. (2015). Oxytocin receptor gene variation and differential susceptibility to family environment in predicting youth borderline symptoms. *Journal of Personality Disorders*, *29*(2), 177-192.

Henquet, C., Krabbendam, L., de Graaf, R., ten Have, M., & van Os, J. (2006). Cannabis use and expression of mania in the general population. *Journal of Affective Disorders*, *95*(1-3), 103-110.

Hillegers, M. H., Reichart, C. G., Wals, M., Verhulst, F. C., Ormel, J., & Nolen, W. A. (2005). Five‐year prospective outcome of psychopathology in the adolescent offspring of bipolar parents. *Bipolar Disorders*, *7*(4), 344-350.

Holma, K. M., Melartin, T. K., Holma, I. A., & Isometsä, E. T. (2008). Predictors for switch from unipolar major depressive disorder to bipolar disorder type I or II: a 5-year prospective study. *The Journal of Clinical Psychiatry*, *69*(8), 15174.

Homish, G. G., Marshall, D., Dubovsky, S. L., & Leonard, K. (2013). Predictors of later bipolar disorder in patients with subthreshold symptoms. *Journal of Affective Disorders*, *144*(1-2), 129-133.

Iorfino, F., Scott, E. M., Carpenter, J. S., Cross, S. P., Hermens, D. F., Killedar, M., ... Hickie, I. B. (2019). Clinical stage transitions in persons aged 12 to 25 years presenting to early intervention mental health services with anxiety, mood, and psychotic disorders. *JAMA Psychiatry*, *76*(11), 1167-1175.

Johnson, J., Horwath, E., & Weissman, M. M. (1991). The validity of major depression with psychotic features based on a community study. *Archives of General Psychiatry*, *48*(12), 1075-1081.

Johnson, J. G., Cohen, P., Brown, J., Smailes, E. M., & Bernstein, D. P. (1999). Childhood maltreatment increases risk for personality disorders during early adulthood. *Archives of General Psychiatry*, *56*(7), 600-606.

Johnson, J. G., Cohen, P., Chen, H., Kasen, S., & Brook, J. S. (2006). Parenting behaviors associated with risk for offspring personality disorder during adulthood. *Archives of General Psychiatry*, *63*(5), 579-587.

Johnson, J. G., Smailes, E. M., Cohen, P., Brown, J., & Bernstein, D. P. (2000). Associations between four types of childhood neglect and personality disorder symptoms during adolescence and early adulthood: Findings of a community-based longitudinal study. *Journal of Personality Disorders*, *14*(2), 171.

Jonsson, U., Bohman, H., von Knorring, L., Olsson, G., Paaren, A., & Von Knorring, A. L. (2011). Mental health outcome of long-term and episodic adolescent depression: 15-year follow-up of a community sample. *Journal of Affective Disorders*, *130*(3), 395-404.

Jovev, M., McKenzie, T., Whittle, S., Simmons, J. G., Allen, N. B., & Chanen, A. M. (2013). Temperament and maltreatment in the emergence of borderline and antisocial personality pathology during early adolescence. *Journal of the Canadian Academy of Child and Adolescent Psychiatry*, *22*(3), 220.

Judd, L. L., Schettler, P. J., Coryell, W., Akiskal, H. S., & Fiedorowicz, J. G. (2013). Overt irritability/anger in unipolar major depressive episodes: past and current characteristics and implications for long-term course. *JAMA Psychiatry*, *70*(11), 1171-1180.

Kane, J. M., Quitkin, F. M., Rifkin, A., Ramos-Lorenzi, J. R., Nayak, D. D., & Howard, A. (1982). Lithium carbonate and imipramine in the prophylaxis of unipolar and bipolar II illness: a prospective, placebo-controlled comparison. *Archives of General Psychiatry*, *39*(9), 1065-1069.

Kaymaz, N., van Os, J., de Graaf, R., Ten Have, M., Nolen, W., & Krabbendam, L. (2007). The impact of subclinical psychosis on the transition from subclinicial mania to bipolar disorder. *Journal of Affective Disorders*, *98*(1-2), 55-64.

Klein, R. G., Mannuzza, S., Olazagasti, M. A. R., Roizen, E., Hutchison, J. A., Lashua, E. C., & Castellanos, F. X. (2012). Clinical and functional outcome of childhood attention-deficit/hyperactivity disorder 33 years later. *Archives of General Psychiatry*, *69*(12), 1295-1303.

Klimes-Dougan, B., Long, J. D., Lee, C. Y. S., Ronsaville, D. S., Gold, P. W., & Martinez, P. E. (2010). Continuity and cascade in offspring of bipolar parents: a longitudinal study of externalizing, internalizing, and thought problems. *Development and Psychopathology*, *22*(4), 849-866.

Klimes-Dougan, B., Desjardins, C. D., James, M. G., Narayan, A. J., Long, J. D., Cullen, K. R., ... Martinez, P. E. (2013). The development of thought problems: a longitudinal family risk study of offspring of bipolar, unipolar, and well parents. *Development and Psychopathology*, *25*(4pt1), 1079-1091.

Kochman, F. J., Hantouche, E. G., Ferrari, P., Lancrenon, S., Bayart, D., & Akiskal, H. S. (2005). Cyclothymic temperament as a prospective predictor of bipolarity and suicidality in children and adolescents with major depressive disorder. *Journal of Affective Disorders*, *85*(1-2), 181-189.

Kovacs, M., Akiskal, H. S., Gatsonis, C., & Parrone, P. L. (1994). Childhood-onset dysthymic disorder: Clinical features and prospective naturalistic outcome. *Archives of General Psychiatry*, *51*(5), 365-374.

Kuehner, C., & Huffziger, S. (2012). Response styles to depressed mood affect the long-term course of psychosocial functioning in depressed patients. *Journal of Affective Disorders*, *136*(3), 627-633.

Krabbendam, A. A., Colins, O. F., Doreleijers, T. A., van der Molen, E., Beekman, A. T., & Vermeiren, R. R. (2015). Personality disorders in previously detained adolescent females: A prospective study. *American Journal of Orthopsychiatry*, *85*(1), 63.

Kwapil, T. R., Miller, M. B., Zinser, M. C., Chapman, L. J., Chapman, J., & Eckblad, M. (2000). A longitudinal study of high scorers on the hypomanic personality scale. *Journal of Abnormal Psychology*, *109*(2), 222.

LaRoche, C., Sheiner, R., Lester, E., Benierakis, C., Marrache, M., Engelsmann, F., & Cheifetz, P. (1987). Children of parents with manic-depressive illness: a follow-up study. *The Canadian Journal of Psychiatry*, *32*(7), 563-569.

Lazarus, S. A., Choukas-Bradley, S., Beeney, J. E., Byrd, A. L., Vine, V., & Stepp, S. D. (2019). Too much too soon?: borderline personality disorder symptoms and romantic relationships in adolescent girls. *Journal of Abnormal Child Psychology*, *47*(12), 1995-2005.

Levenson, J. C., Axelson, D. A., Merranko, J., Angulo, M., Goldstein, T. R., Mullin, B. C., ... Birmaher, B. (2015). Differences in sleep disturbances among offspring of parents with and without bipolar disorder: association with conversion to bipolar disorder. *Bipolar Disorders*, *17*(8), 836-848.

Lenzenweger, M. F., & Castro, D. D. (2005). Predicting change in borderline personality: Using neurobehavioral systems indicators within an individual growth curve framework. *Development and Psychopathology*, *17*(4), 1207-1237.

Lereya, S. T., Winsper, C., Tang, N. K., & Wolke, D. (2017). Sleep problems in childhood and borderline personality disorder symptoms in early adolescence. *Journal of Abnormal Child Psychology*, *45*(1), 193-206.

Levenson, J. C., Soehner, A., Rooks, B., Goldstein, T. R., Diler, R., Merranko, J., ... Birmaher, B. (2017). Longitudinal sleep phenotypes among offspring of bipolar parents and community controls. *Journal of Affective Disorders*, *215*, 30-36.

Lyons-Ruth, K., Bureau, J. F., Holmes, B., Easterbrooks, A., & Brooks, N. H. (2013). Borderline symptoms and suicidality/self-injury in late adolescence: Prospectively observed relationship correlates in infancy and childhood. *Psychiatry Research*, *206*(2-3), 273-281.

Li, Z., Zhang, C., Fan, J., Yuan, C., Huang, J., Chen, J., ... Fang, Y. (2014). Brain-derived neurotrophic factor levels and bipolar disorder in patients in their first depressive episode: 3-year prospective longitudinal study. *The British Journal of Psychiatry*, *205*(1), 29-35.

Maj, M., Pirozzi, R., Magliano, L., Fiorillo, A., & Bartoli, L. (2007). Phenomenology and prognostic significance of delusions in major depressive disorder: a 10-year prospective follow-up study. *Journal of Clinical Psychiatry*, *68*(9), 1411-1417.

McCAULEY, E., Myers, K., Mitchell, J., Calderon, R., Schloredt, K., & Treder, R. (1993). Depression in young people: initial presentation and clinical course. *Journal of the American Academy of Child & Adolescent Psychiatry*, *32*(4), 714-722.

Melvin, G. A., Dudley, A. L., Gordon, M. S., Ford, S., Taffe, J., & Tonge, B. J. (2013). What happens to depressed adolescents? A follow-up study into early adulthood. *Journal of Affective Disorders*, *151*(1), 298-305.

Meijer, M., Goedhart, A. W., & Treffers, P. D. (1998). The persistence of borderline personality disorder in adolescence. *Journal of Personality Disorders*, *12*(1), 13.

Mesman, E., Nolen, W. A., Reichart, C. G., Wals, M., & Hillegers, M. H. (2013). The Dutch bipolar offspring study: 12-year follow-up. *American Journal of Psychiatry*, *170*(5), 542-549.

Mesman, E., Nolen, W. A., Keijsers, L., & Hillegers, M. H. J. (2017). Baseline dimensional psychopathology and future mood disorder onset: findings from the Dutch Bipolar Offspring Study. *Acta Psychiatrica Scandinavica*, *136*(2), 201-209.

Miller, C. J., Flory, J. D., Miller, S. R., Harty, S. C., Newcorn, J. H., & Halperin, J. M. (2008). Childhood attention-deficit/hyperactivity disorder and the emergence of personality disorders in adolescence: a prospective follow-up study. *Journal of Clinical Psychiatry*, *69*(9), 1477.

Nadkarni, R. B., & Fristad, M. A. (2010). Clinical course of children with a depressive spectrum disorder and transient manic symptoms. *Bipolar Disorders*, *12*(5), 494-503.

Nery, F. G., Wilson, A. R., Schneider, M. R., Strawn, J. R., Patino, L. R., McNamara, R. K., ... DelBello, M. P. (2020). Medication exposure and predictors of first mood episode in offspring of parents with bipolar disorder: a prospective study. *Brazilian Journal of Psychiatry*, *42*, 481-488.

Nurnberger, J. I., McInnis, M., Reich, W., Kastelic, E., Wilcox, H. C., Glowinski, A., ... Monahan, P. O. (2011). A high-risk study of bipolar disorder: childhood clinical phenotypes as precursors of major mood disorders. *Archives of General Psychiatry*, *68*(10), 1012-1020.

Opjordsmoen, S. (1989). Long‐term course and outcome in unipolar affective and schizoaffective psychoses. *Acta Psychiatrica Scandinavica*, *79*(4), 317-326.

Papachristou, E., Ormel, J., Oldehinkel, A. J., Kyriakopoulos, M., Reinares, M., Reichenberg, A., & Frangou, S. (2013). Child Behavior Checklist—Mania Scale (CBCL-MS): Development and evaluation of a population-based screening scale for bipolar disorder. *PloS One*, *8*(8), e69459.

Papachristou, E., Oldehinkel, A. J., Ormel, J., Raven, D., Hartman, C. A., Frangou, S., & Reichenberg, A. (2017). The predictive value of childhood subthreshold manic symptoms for adolescent and adult psychiatric outcomes. *Journal of Affective Disorders*, *212*, 86-92.

Päären, A., Bohman, H., von Knorring, L., Olsson, G., von Knorring, A. L., & Jonsson, U. (2014). Early risk factors for adult bipolar disorder in adolescents with mood disorders: a 15-year follow-up of a community sample. *BMC psychiatry*, *14*(1), 1-13.

Perich, T., Lau, P., Hadzi-Pavlovic, D., Roberts, G., Frankland, A., Wright, A., ... Mitchell, P. B. (2015). What clinical features precede the onset of bipolar disorder? *Journal of Psychiatric Research*, *62*, 71-77.

Pfennig, A., Ritter, P. S., Höfler, M., Lieb, R., Bauer, M., Wittchen, H. U., & Beesdo‐Baum, K. (2016). Symptom characteristics of depressive episodes prior to the onset of mania or hypomania. *Acta Psychiatrica Scandinavica*, *133*(3), 196-204.

Preisig, M., Strippoli, M. P. F., Castelao, E., Merikangas, K. R., Gholam-Rezaee, M., Marquet, P., ... Vandeleur, C. L. (2016). The specificity of the familial aggregation of early-onset bipolar disorder: a controlled 10-year follow-up study of offspring of parents with mood disorders. *Journal of Affective Disorders*, *190*, 26-33.

Prien, R. F., Kupfer, D. J., Mansky, P. A., Small, J. G., Tuason, V. B., Voss, C. B., & Johnson, W. E. (1984). Drug therapy in the prevention of recurrences in unipolar and bipolar affective disorders: report of the NIMH Collaborative Study Group comparing lithium carbonate, imipramine, and a lithium carbonate-imipramine combination. *Archives of General psychiatry*, *41*(11), 1096-1104.

Radke-Yarrow, M., Nottelmann, E., Martinez, P., Fox, M. B., & Belmont, B. (1992). Young children of affectively ill parents: A longitudinal study of psychosocial development. *Journal of the American Academy of Child & Adolescent Psychiatry*, *31*(1), 68-77.

Ramklint, M., von Knorring, A-L., von Knorring, L., & Ekselius, L. (2003). Child and adolescent psychiatric disorders predicting adult personality disorder: a follow-up study. *Nordic Journal of Psychiatry*, *57*(1), 23-28.

Rao, U. M. A., Ryan, N. D., Birmaher, B., Dahl, R. E., Williamson, D. E., Kaufman, J., ... Nelson, B. (1995). Unipolar depression in adolescents: clinical outcome in adulthood. *Journal of the American Academy of Child & Adolescent Psychiatry*, *34*(5), 566-578.

Rao, U., Dahl, R. E., Ryan, N. D., Birmaher, B., Williamson, D. E., Rao, R., & Kaufman, J. (2002). Heterogeneity in EEG sleep findings in adolescent depression: unipolar versus bipolar clinical course. *Journal of Affective Disorders*, *70*(3), 273-280.

Ratheesh, A., Cotton, S. M., Betts, J. K., Chanen, A., Nelson, B., Davey, C. G., ... Bechdolf, A. (2015). Prospective progression from high-prevalence disorders to bipolar disorder: exploring characteristics of pre-illness stages. *Journal of Affective Disorders*, *183*, 45-48.

Regeer, E. J., Krabbendam, L., De Graaf, R., Ten Have, M., Nolen, W. A., & Van Os, J. (2006). A prospective study of the transition rates of subthreshold (hypo) mania and depression in the general population. *Psychological Medicine*, *36*(5), 619-627.

Reichart, C. G., van der Ende, J., Wals, M., Hillegers, M. H., Nolen, W. A., Ormel, J., & Verhulst, F. C. (2005). The use of the GBI as predictor of bipolar disorder in a population of adolescent offspring of parents with a bipolar disorder. *Journal of Affective Disorders*, *89*(1-3), 147-155.

Reinelt, E., Stopsack, M., Aldinger, M., Ulrich, I., Grabe, H. J., & Barnow, S. (2014). Longitudinal transmission pathways of borderline personality disorder symptoms: from mother to child? *Psychopathology*, *47*(1), 10-16.

Rey, J. M., Morris-Yates, A., Singh, M., Andrews, G., & Stewart, G. W. (1995). Continuities between psychiatric disorders in adolescents and personality disorders in young adults. *American Journal of Psychiatry*, *152*(6), 895-900.

Riihimäki, K. A., Vuorilehto, M. S., Melartin, T. K., & Isometsä, E. T. (2014). Five-year outcome of major depressive disorder in primary health care. *Psychological Medicine*, *44*(7), 1369-1379.

Ritter, P. S., Höfler, M., Wittchen, H. U., Lieb, R., Bauer, M., Pfennig, A., & Beesdo-Baum, K. (2015). Disturbed sleep as risk factor for the subsequent onset of bipolar disorder–data from a 10-year prospective-longitudinal study among adolescents and young adults. *Journal of Psychiatric Research*, *68*, 76-82.

Rössler, W., Hengartner, M. P., Ajdacic-Gross, V., Haker, H., Gamma, A., & Angst, J. (2011). Sub-clinical psychosis symptoms in young adults are risk factors for subsequent common mental disorders. *Schizophrenia Research*, *131*(1-3), 18-23.

Rudaz, D., Vandeleur, C. L., Gholam, M., Castelao, E., Strippoli, M. P. F., Marquet, P., ... Preisig, M. (2021). Psychopathological precursors of the onset of mood disorders in offspring of parents with and without mood disorders: results of a 13‐year prospective cohort high‐risk study. *Journal of Child Psychology and Psychiatry*, *62*(4), 404-413.

Ruggero, C. J., Kotov, R., Carlson, G. A., Tanenberg-Karant, M., González, D. A., & Bromet, E. J. (2011). Diagnostic consistency of major depression with psychosis across 10 years. *The Journal of Clinical Psychiatry*, *72*(9), 4445.

Salvatore, P., Baldessarini, R. J., Tohen, M., Khalsa, H. M. K., Sanchez-Toledo, J. P., Zarate Jr, C. A., ... Maggini, C. (2009). McLean-Harvard International First-Episode Project: two-year stability of DSM-IV diagnoses in 500 first-episode psychotic disorder patients. *Journal of Clinical Psychiatry*, *70*(4), 458.

Salvatore, P., Baldessarini, R. J., Khalsa, H. M. K., Amore, M., Di Vittorio, C., Ferraro, G., ... Tohen, M. (2013). Predicting diagnostic change among patients diagnosed with first-episode DSM-IV-TR major depressive disorder with psychotic features. *The Journal of Clinical Psychiatry*, *74*(7), 14983.

Scott, J., Marwaha, S., Ratheesh, A., Macmillan, I., Yung, A. R., Morriss, R., ... Bechdolf, A. (2017). Bipolar at-risk criteria: an examination of which clinical features have optimal utility for identifying youth at risk of early transition from depression to bipolar disorders. *Schizophrenia Bulletin*, *43*(4), 737-744.

Scott, J., Byrne, E., Medland, S., & Hickie, I. (2020). Self-reported sleep-wake disturbances preceding onset of full-threshold mood and/or psychotic syndromes in community residing adolescents and young adults. *Journal of Affective Disorders*, *277*, 592-595.

Scott, J., Byrne, E., Medland, S., & Hickie, I. (2020). Self-reported sleep-wake disturbances preceding onset of full-threshold mood and/or psychotic syndromes in community residing adolescents and young adults. *Journal of Affective Disorders*, *277*, 592-595.

Selby, E. A. (2013). Chronic sleep disturbances and borderline personality disorder symptoms. *Journal of Consulting and Clinical Psychology*, *81*(5), 941.

Selby, E. A., & Yen, S. (2014). Six‐month trajectory of suicidal ideation in adolescents with borderline personality disorder. *Suicide and Life‐Threatening Behavior*, *44*(1), 89-100.

Sharp, C., Vanwoerden, S., Jouriles, E. N., Godfrey, D. A., Babcock, J., McLaren, V., ... Temple, J. R. (2020). Exposure to interparental intimate partner violence and the development of borderline features in adolescents. *Child Abuse & Neglect*, *103*, 104448.

Sharp, C., Kalpakci, A., Mellick, W., Venta, A., & Temple, J. R. (2015). First evidence of a prospective relation between avoidance of internal states and borderline personality disorder features in adolescents. *European Child & Adolescent psychiatry*, *24*(3), 283-290.

Shaw, J. A., Egeland, J. A., Endicott, J., Allen, C. R., & Hostetter, A. M. (2005). A 10-year prospective study of prodromal patterns for bipolar disorder among Amish youth. *Journal of the American Academy of Child & Adolescent Psychiatry*, *44*(11), 1104-1111.

Schwartz, J. E., Fennig, S., Tanenberg-Karant, M., Carlson, G., Craig, T., Galambos, N., ... Bromet, E. J. (2000). Congruence of diagnoses 2 years after a first-admission diagnosis of psychosis. *Archives of General Psychiatry*, *57*(6), 593-600.

Sharma, V., Xie, B., Campbell, M. K., Penava, D., Hampson, E., Mazmanian, D., & Pope, C. J. (2014). A prospective study of diagnostic conversion of major depressive disorder to bipolar disorder in pregnancy and postpartum. *Bipolar Disorders*, *16*(1), 16-21.

Sharp, C., Kalpakci, A., Mellick, W., Venta, A., & Temple, J. R. (2015). First evidence of a prospective relation between avoidance of internal states and borderline personality disorder features in adolescents. *European Child & Adolescent Psychiatry*, *24*(3), 283-290.

Shen, G. H., Sylvia, L. G., Alloy, L. B., Barrett, F., Kohner, M., Iacoviello, B., & Mills, A. (2008). Lifestyle regularity and cyclothymic symptomatology. *Journal of Clinical Psychology*, *64*(4), 482-500.

Shur-Fen Gau, S., Lin, Y. J., Tai-Ann Cheng, A., Chiu, Y. N., Tsai, W. C., & Soong, W. T. (2010). Psychopathology and symptom remission at adolescence among children with attention-deficit–hyperactivity disorder. *Australian & New Zealand Journal of Psychiatry*, *44*(4), 323-332.

Solomon, D. A., Keller, M. B., Leon, A. C., Mueller, T. I., Shea, M. T., Warshaw, M., ... Endicott, J. (1997). Recovery from major depression: a 10-year prospective follow-up across multiple episodes. *Archives of General Psychiatry*, *54*(11), 1001-1006.

Stepp, S. D., Burke, J. D., Hipwell, A. E., & Loeber, R. (2012). Trajectories of attention deficit hyperactivity disorder and oppositional defiant disorder symptoms as precursors of borderline personality disorder symptoms in adolescent girls. *Journal of Abnormal Child Psychology*, *40*(1), 7-20.

Stepp, S. D., Olino, T. M., Klein, D. N., Seeley, J. R., & Lewinsohn, P. M. (2013). Unique influences of adolescent antecedents on adult borderline personality disorder features. *Personality Disorders: Theory, Research, and Treatment*, *4*(3), 223.

Stepp, S. D., Keenan, K., Hipwell, A. E., & Krueger, R. F. (2014). The impact of childhood temperament on the development of borderline personality disorder symptoms over the course of adolescence. *Borderline Personality Disorder and Emotion Dysregulation*, *1*(1), 1-10.

Stepp, S. D., Whalen, D. J., Scott, L. N., Zalewski, M., Loeber, R., & Hipwell, A. E. (2014). Reciprocal effects of parenting and borderline personality disorder symptoms in adolescent girls. *Development and Psychopathology*, *26*(2), 361-378.

Stepp, S. D., Scott, L. N., Jones, N. P., Whalen, D. J., & Hipwell, A. E. (2016). Negative emotional reactivity as a marker of vulnerability in the development of borderline personality disorder symptoms. *Development and Psychopathology*, *28*(1), 213-224.

Stepp, S. D., & Lazarus, S. A. (2017). Identifying a borderline personality disorder prodrome: implications for community screening. *Personality and Mental Health*, *11*(3), 195-205.

Strandholm, T., Kiviruusu, O., Karlsson, L., Pankakoski, M., Pelkonen, M., & Marttunen, M. (2017). Stability and change in personality disorder symptoms in 1-year follow-up of depressed adolescent outpatients. *The Journal of Nervous and Mental Disease*, *205*(1), 15-22.

Stoléru, S., Nottelmann, E. D., Belmont, B., & Ronsaville, D. (1997). Sleep problems in children of affectively ill mothers. *Journal of Child Psychology and Psychiatry*, *38*(7), 831-841.

Strober, M., & Carlson, G. (1982). Bipolar illness in adolescents with major depression: clinical, genetic, and psychopharmacologic predictors in a three-to four-year prospective follow-up investigation. *Archives of General Psychiatry*, *39*(5), 549-555.

Strober, M., Lampert, C., Schmidt, S., & Morrell, W. (1993). The course of major depressive disorder in adolescents: I. Recovery and risk of manic switching in a follow-up of psychotic and nonpsychotic subtypes. *Journal of the American Academy of Child & Adolescent Psychiatry*, *32*(1), 34-42.

Thatcher, D. L., Cornelius, J. R., & Clark, D. B. (2005). Adolescent alcohol use disorders predict adult borderline personality. *Addictive Behaviors*, *30*(9), 1709-1724.

Thomsen, P. H., & Mikkelsen, H. U. (1993). Development of personality disorders in children and adolescents with obsessive‐compulsive disorder A 6‐to 22‐year follow‐up study. *Acta Psychiatrica Scandinavica*, *87*(6), 456-462.

Tillman, R., & Geller, B. (2006). Controlled study of switching from attention-deficit/hyperactivity disorder to a prepubertal and early adolescent bipolar I disorder phenotype during 6-year prospective follow-up: rate, risk, and predictors. *Development and Psychopathology*, *18*(4), 1037-1053.

Tijssen, M. J., van Os, J., Wittchen, H. U., Lieb, R., Beesdo, K., Mengelers, R., & Wichers, M. (2010). Prediction of transition from common adolescent bipolar experiences to bipolar disorder: 10-year study. *The British Journal of Psychiatry*, *196*(2), 102-108.

Tijssen, M. J., Van Os, J., Wittchen, H. U., Lieb, R., Beesdo, K., & Wichers, M. (2010). Risk factors predicting onset and persistence of subthreshold expression of bipolar psychopathology among youth from the community. *Acta Psychiatrica Scandinavica*, *122*(3), 255-266.

Tohen, M., Khalsa, H. M. K., Salvatore, P., Vieta, E., Ravichandran, C., & Baldessarini, R. J. (2012). Two-year outcomes in first-episode psychotic depression: The McLean–Harvard first-episode project. *Journal of Affective Disorders*, *136*(1-2), 1-8.

Tondo, L., Visioli, C., Preti, A., & Baldessarini, R. J. (2014). Bipolar disorders following initial depression: modeling predictive clinical factors. *Journal of Affective Disorders*, *167*, 44-49.

Tragesser, S. L., Solhan, M., Brown, W. C., Tomko, R. L., Bagge, C., & Trull, T. J. (2010). Longitudinal associations in borderline personality disorder features: Diagnostic Interview for Borderlines—Revised (DIB-R) scores over time. *Journal of Personality Disorders*, *24*(3), 377.

Tragesser, S. L., Solhan, M., Schwartz-Mette, R., & Trull, T. J. (2007). The role of affective instability and impulsivity in predicting future BPD features. *Journal of Personality Disorders*, *21*(6), 603-614.

Vanwoerden, S., Leavitt, J., Gallagher, M. W., Temple, J. R., & Sharp, C. (2019). Dating violence victimization and borderline personality pathology: Temporal associations from late adolescence to early adulthood. *Personality Disorders: Theory, Research, and Treatment*, *10*(2), 132.

Van Meter, A. R., Hafeman, D. M., Merranko, J., Youngstrom, E. A., Birmaher, B. B., Fristad, M. A., ... Findling, R. L. (2021). Generalizing the prediction of bipolar disorder onset across high-risk populations. *Journal of the American Academy of Child & Adolescent Psychiatry*, *60*(8), 1010-1019.

Vaillancourt, T., Brittain, H. L., McDougall, P., Krygsman, A., Boylan, K., Duku, E., & Hymel, S. (2014). Predicting borderline personality disorder symptoms in adolescents from childhood physical and relational aggression, depression, and attention-deficit/hyperactivity disorder. *Development and Psychopathology*, *26*(3), 817-830.

Weintraub, S. (1987). Risk factors in schizophrenia: The Stony Brook high-risk project. *Schizophrenia Bulletin*, *13*(3), 439-450.

Weissman, M. M., Wolk, S., Wickramaratne, P., Goldstein, R. B., Adams, P., Greenwald, S., ... Steinberg, D. (1999). Children with prepubertal-onset major depressive disorder and anxiety grown up. *Archives of General Psychiatry*, *56*(9), 794-801.

Weissman, M. M., Wolk, S., Goldstein, R. B., Moreau, D., Adams, P., Greenwald, S., ... Wickramaratne, P. (1999). Depressed adolescents grown up. *JAMA Psychiatry*, *281*(18), 1707-1713.

Widom, C., Czaja, S. J., & Paris, J. (2009). A prospective investigation of borderline personality disorder in abused and neglected children followed up into adulthood. *Journal of Personality Disorders*, *23*(5), 433.

Winokur, G., & Morrison, J. (1973). The Iowa 500: follow-up of 225 depressives. *The British Journal of Psychiatry*, *123*(576), 543-548.

Winokur, G., & Wesner, R. (1987). From unipolar depression to bipolar illness: 29 who changed. *Acta Psychiatrica Scandinavica*, *76*(1), 59-63.

Winsper, C., Wolke, D., & Lereya, T. (2015). Prospective associations between prenatal adversities and borderline personality disorder at 11–12 years. *Psychological Medicine*, *45*(5), 1025-1037.

Winsper, C., Zanarini, M., & Wolke, D. (2012). Prospective study of family adversity and maladaptive parenting in childhood and borderline personality disorder symptoms in a non-clinical population at 11 years. *Psychological Medicine*, *42*(11), 2405-2420.

Wolke, D., Schreier, A., Zanarini, M. C., & Winsper, C. (2012). Bullied by peers in childhood and borderline personality symptoms at 11 years of age: a prospective study. *Journal of Child Psychology and Psychiatry*, *53*(8), 846-855.

Zahn-Waxler, C., Mayfield, A., Radke-Yarrow, M., McKnew, D. H., Cytryn, L., & Davenport, Y. B. (1988). A follow-up investigation of offspring of parents with bipolar disorder. The American Journal of Psychiatry, 145(4), 506–509. [https://doi.org/10.1176/ajp.145.4.506](https://psycnet.apa.org/doi/10.1176/ajp.145.4.506)

Zimmermann, P., Brückl, T., Nocon, A., Pfister, H., Lieb, R., Wittchen, H. U., ... Angst, J. (2009). Heterogeneity of DSM-IV major depressive disorder as a consequence of subthreshold bipolarity. *Archives of General Psychiatry*, *66*(12), 1341-1352.

**Supplementary 2.** Search Strategy

PUBMED

**#1 ("bipolar disorder"[All Fields] OR bipolar [All Fields] OR "hypomania"[All Fields] OR "cyclothymic"[All Fields] OR "bipolar disorder"[MeSH Terms] OR "hypomani*"[All Fields] OR manic*[All Fields] OR mania*[All Fields] OR "cyclothym*"[All Fields] OR BD-I[All Fields] OR BD-II[All Fields] OR "bipolar*"[All Fields] OR borderline personality disorder[MeSH Terms] OR "borderline personalit*"[All Fields] OR "BPD"[All Fields] OR "borderline personality disorder"[All Fields] OR "borderline personality"[All Fields] OR “emotionally unstable personality disorder”[All Fields]** OR “borderline” **[All Fields]** OR “pediatric bipolar” **[All Fields])** Filters: Meta-Analysis, Systematic Review, Child: birth-18 years, Newborn: birth-1 month, Infant: birth-23 months, Infant: 1-23 months, Preschool Child: 2-5 years, Child: 6-12 years, Adolescent: 13-18 years, Young Adult: 19-24 years

**#2** "precursor*"[All Fields] OR "anteceden*"[All Fields] OR "predict*"[All Fields] OR "prodrom*"[All Fields] OR genomic*, OR biologic* OR "polygenic risk score*" OR "genetic*" OR inciden* OR onset* OR biomarker* OR etiolog* OR aetiolog* OR "life-course trajector*" OR at-risk* OR high-risk* OR risk* OR "clinical characteristic*" OR "clinical feature*" OR "clinical manifestation*" OR "predisposing factor*" OR neurobiologic* OR genotype* OR phenotype* OR "temperamental" OR "causal pathway*" OR environmental OR socioeconomic* OR risk* OR predict* or diagnos* or develop* or risk factor[MeSH Terms] Filters: Meta-Analysis, Systematic Review, Child: birth-18 years, Newborn: birth-1 month, Infant: birth-23 months, Infant: 1-23 months, Preschool Child: 2-5 years, Child: 6-12 years, Adolescent: 13-18 years, Young Adult: 19-24 years

#3 **("longitudinal study"[All Fields] OR "longitudinal"[All Fields] OR "prospective study"[All Fields] OR "prospect*"[All Fields] OR "follow up"[All Fields] OR "birth cohort" OR "population cohort" OR “prospective study”[MeSH Terms] OR “follow up study”[MeSH Terms] OR “longitudinal study”[MeSH Terms])** Filters: Meta-Analysis, Systematic Review, Child: birth-18 years, Newborn: birth-1 month, Infant: birth-23 months, Infant: 1-23 months, Preschool Child: 2-5 years, Child: 6-12 years, Adolescent: 13-18 years, Young Adult: 19-24 years

#4 **#1 AND #2 AND #3** Filters: Meta-Analysis, Systematic Review, Child: birth-18 years, Newborn: birth-1 month, Infant: birth-23 months, Infant: 1-23 months, Preschool Child: 2-5 years, Child: 6-12 years, Adolescent: 13-18 years, Young Adult: 19-24 years

EMBASE

#1 exp bipolar disorder/ or exp bipolar i disorder/ or exp bipolar ii disorder/ or exp cyclothymic disorder/ or exp mania/ or exp bipolar mania/ or exp cyclothymia/ or exp "mixed mania and depression"/ or exp rapid cycling bipolar disorder/ or 'manic depress*'.mp. or 'bipolar disorder*'.mp. or cyclothymi*.mp. or manic.mp. or mania.mp. or exp borderline personality disorder/ or 'borderline personality'.mp. or 'emotionally unstable personality disorder'.mp. or borderline.mp. or 'pediatric bipolar'.mp. or 'bipolar feature*'.mp. or bipolar.mp. [mp=title, abstract, heading word, drug trade name, original title, device manufacturer, drug manufacturer, device trade name, keyword, floating subheading word, candidate term word]

#2 (longitudinal or prospect* or follow-up* or 'follow up*').mp. or exp Longitudinal Studies/ or 'birth cohort'.mp. or 'population cohort'.mp. or exp prospective study/ or exp follow up/ [mp=title, abstract, heading word, drug trade name, original title, device manufacturer, drug manufacturer, device trade name, keyword, floating subheading word, candidate term word]

#3 exp risk factors/ or precursor*.mp. or antecedent*.mp. or prodrom*.mp. or biologic*.mp. or genomic*.mp. or genetic*.mp. or inciden*.mp. or onset*.mp. or biomarker*.mp. or "polygenic risk score*".mp. or etiolog*.mp. or aetiolog*.mp. or "life-course trajector*".mp. or at-risk*.mp. or high-risk*.mp. or "clinical characteristic*".mp. or "clinical feature*".mp. or "clinical manifestation*".mp. or "predisposing factor*".mp. or neurobiologic*.mp. or genotype*.mp. or phenotype*.mp. or "temperamental".mp. or environmental.mp. or socioeconomic*.mp. or "causal pathway*".mp. or exp risk/ or exp diagnosis/ or exp development/ or risk*.mp. or develop*.mp. or diagno*.mp. or predict*.mp. or exp biological marker/ or exp genotype/ or exp phenotype/ or exp etiology/ or exp precursor/ or exp clinical feature/ or exp socioeconomics/ or exp environmental factor/ or exp incidence/

#4 ('meta analysis' or meta-analysis or 'meta review' or 'systematic review').mp. or exp meta analysis/ or exp "systematic review"/ [mp=title, abstract, heading word, drug trade name, original title, device manufacturer, drug manufacturer, device trade name, keyword, floating subheading word, candidate term word]

#5 (child* or "young adult" or "young people" or adolescen* or infant* or boy* or girl* or newborn* or youth* or teen* or student* or baby or young*).mp. or exp child/ or exp adolescent/ or exp young adult/ or exp infant/ or exp boy/ or exp girl/ or exp baby/ or exp newborn/ [mp=title, abstract, heading word, drug trade name, original title, device manufacturer, drug manufacturer, device trade name, keyword, floating subheading word, candidate term word]

#6 1 and 2 and 3 and 4 and 5

MEDLINE

#1 exp bipolar disorder/ or exp bipolar i disorder/ or exp bipolar ii disorder/ or exp cyclothymic disorder/ or exp mania/ or exp bipolar mania/ or exp cyclothymia/ or exp "mixed mania and depression"/ or exp rapid cycling bipolar disorder/ or 'manic depress*'.mp. or 'bipolar disorder*'.mp. or cyclothymi*.mp. or manic.mp. or mania.mp. or exp borderline personality disorder/ or 'borderline personality'.mp. or 'emotionally unstable personality disorder'.mp. or borderline.mp. or 'pediatric bipolar'.mp. or 'bipolar feature*'.mp. or bipolar.mp. [mp=title, abstract, original title, name of substance word, subject heading word, floating sub-heading word, keyword heading word, organism supplementary concept word, protocol supplementary concept word, rare disease supplementary concept word, unique identifier, synonyms]

#2 (longitudinal or prospect* or follow-up* or 'follow up*').mp. or exp Longitudinal Studies/ or 'birth cohort'.mp. or 'population cohort'.mp. or exp prospective study/ or exp follow up/ [mp=title, abstract, original title, name of substance word, subject heading word, floating sub-heading word, keyword heading word, organism supplementary concept word, protocol supplementary concept word, rare disease supplementary concept word, unique identifier, synonyms]

#3 ('meta analysis' or meta-analysis or 'meta review' or 'systematic review').mp. or exp meta analysis/ or exp "systematic review"/ [mp=title, abstract, original title, name of substance word, subject heading word, floating sub-heading word, keyword heading word, organism supplementary concept word, protocol supplementary concept word, rare disease supplementary concept word, unique identifier, synonyms]

#4 (child* or "young adult" or "young people" or adolescen* or infant* or boy* or girl* or newborn* or youth* or teen* or student* or baby or young*).mp. or exp child/ or exp adolescent/ or exp young adult/ or exp infant/ or exp boy/ or exp girl/ or exp baby/ or exp newborn/ [mp=title, abstract, original title, name of substance word, subject heading word, floating sub-heading word, keyword heading word, organism supplementary concept word, protocol supplementary concept word, rare disease supplementary concept word, unique identifier, synonyms]

#5 exp risk factors/ or precursor*.mp. or antecedent*.mp. or prodrom*.mp. or biologic*.mp. or genomic*.mp. or genetic*.mp. or inciden*.mp. or onset*.mp. or biomarker*.mp. or "polygenic risk score*".mp. or etiolog*.mp. or aetiolog*.mp. or "life-course trajector*".mp. or at-risk*.mp. or high-risk*.mp. or "clinical characteristic*".mp. or "clinical feature*".mp. or "clinical manifestation*".mp. or "predisposing factor*".mp. or neurobiologic*.mp. or genotype*.mp. or phenotype*.mp. or "temperamental".mp. or environmental.mp. or socioeconomic*.mp. or "causal pathway*".mp. or exp risk/ or exp diagnosis/ or exp development/ or risk*.mp. or develop*.mp. or diagno*.mp. or predict*.mp. or exp biological marker/ or exp genotype/ or exp phenotype/ or exp etiology/ or exp precursor/ or exp clinical feature/ or exp socioeconomics/ or exp environmental factor/ or exp incidence/ or exp diagnosis/

#6 1 and 2 and 3 and 4 and 5.

APA PsychINFO

#1 exp bipolar disorder/ or exp bipolar i disorder/ or exp bipolar ii disorder/ or exp cyclothymic disorder/ or exp mania/ or exp bipolar mania/ or exp cyclothymia/ or exp "mixed mania and depression"/ or exp rapid cycling bipolar disorder/ or 'manic depress*'.mp. or 'bipolar disorder*'.mp. or cyclothymi*.mp. or manic.mp. or mania.mp. or exp borderline personality disorder/ or 'borderline personality'.mp. or 'emotionally unstable personality disorder'.mp. or borderline.mp. or 'pediatric bipolar'.mp. or 'bipolar feature*'.mp. or bipolar.mp. [mp=title, abstract, heading word, table of contents, key concepts, original title, tests & measures, mesh]

#2 (longitudinal or prospect* or follow-up* or 'follow up*').mp. or exp Longitudinal Studies/ or 'birth cohort'.mp. or 'population cohort'.mp. or exp prospective study/ or exp follow up/ [mp=title, abstract, heading word, table of contents, key concepts, original title, tests & measures, mesh]

#3 ('meta analysis' or meta-analysis or 'meta review' or 'systematic review').mp. or exp meta analysis/ or exp "systematic review"/ [mp=title, abstract, heading word, table of contents, key concepts, original title, tests & measures, mesh]

#4 (child* or "young adult" or "young people" or adolescen* or infant* or boy* or girl* or newborn* or youth* or teen* or student* or baby or young*).mp. or exp child/ or exp adolescent/ or exp young adult/ or exp infant/ or exp boy/ or exp girl/ or exp baby/ or exp newborn/ [mp=title, abstract, heading word, table of contents, key concepts, original title, tests & measures, mesh]

#5 exp risk factors/ or precursor*.mp. or antecedent*.mp. or prodrom*.mp. or biologic*.mp. or genomic*.mp. or genetic*.mp. or inciden*.mp. or onset*.mp. or biomarker*.mp. or "polygenic risk score*".mp. or etiolog*.mp. or aetiolog*.mp. or "life-course trajector*".mp. or at-risk*.mp. or high-risk*.mp. or "clinical characteristic*".mp. or "clinical feature*".mp. or "clinical manifestation*".mp. or "predisposing factor*".mp. or neurobiologic*.mp. or genotype*.mp. or phenotype*.mp. or "temperamental".mp. or environmental.mp. or socioeconomic*.mp. or "causal pathway*".mp. or exp risk/ or exp diagnosis/ or exp development/ or risk*.mp. or develop*.mp. or diagno*.mp. or predict*.mp. or exp biological marker/ or exp genotype/ or exp phenotype/ or exp etiology/ or exp precursor/ or exp clinical feature/ or exp socioeconomics/ or exp environmental factor/ or exp incidence/ or exp diagnosis/

#6 1 and 2 and 3 and 4 and 5

Web of Science

ALL=('borderline  personality'  OR  'borderline  trait*'  OR  'borderline  feature*'  OR  “emotionally  unstable  personality  disorder”  OR  borderline  OR  'bipolar  disorder'  OR  manic  OR  mania  OR  cychlothymi*  OR  BD-*  OR  hypomani*  or  "bipolar  symptomotology"  or  "pediatric  bipolar"  or  "bipolar  feature*"OR  “manic  depress*”  OR  bipolar)  AND  ALL=(longitudinal  OR  follow-up*  OR  prospect*  OR  "population  cohort*"  OR  "birth  cohort"  OR  "follow  up*")  AND  ALL=(precursor*  OR  antecedent*  OR  prodrom*  OR  biologic*  or  genomic*  or  genetic*  or  inciden*  or  onset*  or  biomarker*  or  "polygenic  risk  score*"  or  etiolog*  or  aetiolog*  or  "life-course  trajectories"  or  at-risk*  or  high-risk*  or  "clinical  characteristic*"  or  "clinical  feature*"  or  "clinical  manifestation*"  or  neurobiologic*  or  genotype*  or  phenotype*  or  "temperamental"  or  environmental  or  socioeconomic*  OR  diagno*  OR  predict*  OR  risk*  OR  develop*)  AND  ALL=('meta  analysis'  or  meta-analysis  or  'meta  review'  or  'systematic  review')  AND  ALL=(child*  or  adolescen*  or  infant*  or  boy*  or  girl*  or  newborn*  or  youth*  or  teen*  or  student*  or  young*)

CINAHL

TX ( 'Bipolar Disorder' OR hypomani* OR manic OR mania OR cyclothymi* OR 'borderline personality' OR 'borderline personality disorder' OR "emotionally unstable personality disorder*" OR borderline OR “pediatric bipolar” OR bipolar) AND TX ( prospect* OR longitudinal OR follow-up* OR "birth cohort" OR "population cohort" or “follow up”) AND TX ( antecedent* OR precursor* OR prodrom* OR inciden* OR biomarker* OR genetic* OR genomic* OR ‘polygenic risk score*’ OR onset* OR biologic* OR etiolog* or aetiolog* or "life-course trajector*" or at-risk* or high-risk* or "clinical characteristic*" or "clinical feature*" or "clinical manifestation*" or "predisposing factor*" or neurobiologic* or genotype* or phenotype* or "temperamental" or environmental or socioeconomic* or 'causal pathways' or risk* or develop* or diagno* or predict*) AND TX (child* OR adolescen* OR ‘young adult*’ OR ‘young people’ OR infant* OR newborn* OR boy* OR girl* OR teen* OR student* OR youth* OR young*) AND TX ('meta analysis' or meta-analysis or 'meta review' or 'systematic review')

COCHRANE

#1 'Bipolar Disorder' OR hypomani* OR manic OR mania OR cyclothymi* OR 'borderline personality' OR 'borderline personality disorder' OR "emotionally unstable personality disorder*" OR borderline OR bipolar OR "manic depress*" OR BD* OR BPD

#2 MeSH descriptor: [Borderline Personality Disorder] explode all trees

#3 MeSH descriptor: [Bipolar Disorder] explode all trees

#4 #1 OR #2 OR #3

#5 MeSH descriptor: [Longitudinal Studies] explode all trees

#6 MeSH descriptor: [Prospective Studies] explode all trees

#7 MeSH descriptor: [Follow-Up Studies] explode all trees

#8 prospect* OR longitudinal OR follow-up* OR "birth cohort" OR "population cohort"

#9 #5 OR #6 OR #7 OR #8

#10 MeSH descriptor: [Risk Factors] explode all trees

#11 antecedent* OR precursor* OR prodrom* OR inciden* OR biomarker* OR genetic* OR genomic* OR ‘polygenic risk score*’ OR onset* OR biologic* OR etiolog* or aetiolog* or "life-course trajector*" or at-risk* or high-risk* or "clinical characteristic*" or "clinical feature*" or "clinical manifestation*" or "predisposing factor*" or "onset pattern*" or neurobiologic* or genotype* or phenotype* or "temperamental" or environmental or socioeconomic* or 'causal pathways' or develop* or predict* or diagno* or risk*

#12 MeSH descriptor: [Risk] explode all trees

#13 MeSH descriptor: [Diagnosis] explode all trees

#14 MeSH descriptor: [Biomarkers] explode all trees

#15 MeSH descriptor: [Phenotype] explode all trees

#16 MeSH descriptor: [Genotype] explode all trees

#17 MeSH descriptor: [Socioeconomic Factors] explode all trees

#18 MeSH descriptor: [Sociological Factors] explode all trees

#19 #11 or #10 or #12 or #13 or #14 or #15 or #16 or #17 or #18

#20 MeSH descriptor: [Child] explode all trees

#21 MeSH descriptor: [Adolescent] explode all trees

#22 MeSH descriptor: [Infant, Newborn] explode all trees

#23 child* or adolescen* or infant* or boy* or girl* or newborn* or youth* or teen* or student* or young*

#24 #20 or #21 #22 #23

#26 #24 AND #4 AND #9 AND #19.

**Supplementary 4.** Vulnerability Factors

**Environmental Factors**

Gender

In the SR/MA by Ratheesh et al. (2011), gender was not a significant predictor of transition to BD. No comparable reviews were available for BPD.

Family History of Psychopathology

Five reviews examined the effects of family history of psychopathology and onset of BD (Keramatian, Chakrabarty, Saraf, & Yatham, 2021; Lau et al., 2017; Narayan, Allen, Cullen, & Klimnes-Dougan, 2013; Rasic, Hajek, Alda, & Uher, 2014; Ratheesh et al. 2017). Most of the primary studies they included demonstrated that offspring of parents with BD, MDD, or psychosis had at least one mental illness diagnosis including BD. Family history of depression, on the other hand, did not predict transition to BD (Ratheesh et al., 2017).

For BPD, data were provided by three reviews (Skabeikyte & Barkauskiene, 2021; Stepp, Lazarus, & Byrd, 2016; Winsper et al., 2016a). Results indicated a moderate to high level of heritability of trait BPD in child and adolescent populations (Winsper et al., 2016a) and maternal psychopathology in general had significant associations with later BPD symptoms (Skabeikyte & Barkauskiene, 2021; Stepp, Lazarus, & Byrd, 2016). However, parental depression did not predict changes in BPD symptoms in the offspring (Skabeikyte & Barkauskiene, 2021).

**Psychosocial Factors**

Childhood Adversity

Data regarding the effect of childhood adversity on risk for BD was provided by two reviews (Palmier-Claus, Berry, Bucci, Mansell, & Varese, 2016; Ratheesh et al., 2017). Findings indicated that severity of childhood abuse, neglect, physical abuse, verbal abuse, and sexual abuse were significantly associated with higher risks of both first onset and recurrent mania.

A greater quantity of primary studies was synthesised by three reviews for BPD than BD regarding the effects of childhood adversity (Skabeikyte & Barkauskiene, 2021; Stepp et al., 2016; Winsper et al., 2016b). Results demonstrated significant associations with relational aggression in the context of friendship, physical and verbal aggression in romantic relationships, physical abuse, parental hostility/ verbal abuse, emotional abuse, childhood neglect and later BPD symptoms. Additionally, peer victimisation was predictive of higher levels of BPD symptoms in adolescents (Stepp et al., 2016).

Family Relations and Adversity

Three reviews studied the effects of family relations on the risk for later BPD symptoms (Skabeikyte & Barkauskiene, 2021; Stepp et al., 2016; Winsper et al., 2016b). Results demonstrated a significant association between parental conflict and higher BPD symptoms (Winsper et al., 2016b). However, results were inconsistent for parent-child relationship quality, family adversity, and later BPD symptoms (Stepp et al., 2016). There were no significant associations between family relations, social support from family or friends and subsequent BPD symptoms (Skabeikyte & Barkauskiene, 2021). The relationship quality with the father, on the other hand, predicted declines in BPD features in adolescence. No comparable evidence was available for BD.

Parenting Style

Two reviews found evidence concerning the association between parenting style and later BPD symptoms (Skabeikyte & Barkauskiene, 2021; Stepp et al., 2016). Inconsistent results were found for low warmth, rejection, low maternal satisfaction with the child, hostility, inconsistent parenting, and harsh punishment/discipline predicting later BPD symptoms (Stepp et al., 2016). Results for intimate partner violence, maternal communication, maternal over-involvement, and maternal expressed negative emotion and later BPD symptoms were consistent (Skabeikyte & Barkauskiene, 2021; Stepp et al., 2016).

Non-significant associations were found for low warmth, parental harsh punishment and maternal support/validation and later BPD (Skabeikyte & Barkauskiene, 2021). No comparable data were available for BD.

General Psychosocial Functioning

One BPD review reported that lower levels of a child’s general psychosocial functioning predicted BPD onset at follow-up (Skabeikyte and Barkauskiene 2021). One BD indicated that poor general psychosocial functioning predicted new onset of BD (Keramatian, 2021).

**Table S1**

Characteristics of systematic reviews and meta-analyses examining the developmental pathways and onset of Bipolar Disorder

| Study (Type) | Sample Size and Characteristics | Factors examined | N/n | Databases searched (last assessed) | Types of studies included (publication date range) | AMSTAR index |
| --- | --- | --- | --- | --- | --- | --- |
| Bart et al. 2021 (SR) | 5495 in total, 392 offspring of BD parents or first-degree relative  “At-risk” individuals based on having a first-degree relative either with BD or SUD, but no current diagnosis themselves and euthymic/remitted samples compared to healthy controls or to in-episode individuals | Neural reward circuit dysfunction | 34/2 | PsychINFO, and PubMed (Up to February 14, 2021) | Prospective and Cross-sectional (2008 – 2020) | 3 |
| Brancati et al. 2021 (SR/MA) | 2823 in total  Only children and adolescents aged ≤ 18 years at first clinical assessment for ADHD diagnosis based on DSM-III-R or subsequent criteria and BD assessment was performed at follow-up | Development of BD in patients with ADHD | 10/10 | Medline, Embase, and Web of Science (NR) | Prospective (2003-2009) | 6 |
| Cardoso et al. 2018 (SR) | 3374 participants in total  Individuals who experienced at least one depressive episode with no history of hypomanic or manic episode | Suicidality | 3/ 3 | PubMed, Bireme, Scopus, and PsychINFO (Up to December 2017) | Prospective (2015-2016) | 4 |
| Cahn et al. 2021 (SR) | 752 participants in total  A BD-I cohort with a current or recent first episode mania, with diagnosis established using standardized assessment criteria | Longitudinal grey matter changes | 15/1 | Medline, Embase, and Web of Science (Up to September 17, 2020) | Prospective (2003-2019) | 6 |
| Faedda et al. 2013 (SR) | 22,048 participants in total  Individuals who are diagnosed with MDE, MDD, dysthymia, cyclothymia, or bipolar NOS as well as subjects with subsyndromal affective disorders or symptoms at intake and bipolar I or bipolar II during follow-up | Precursors of BD | 26/ 26 | PubMed, CINAHL, PsychINFO, EMBASE, SCOPUS, and ISI Web of Science (Up to May 31) | Prospective (1977-2013) | 3 |
| Gibbs et al. 2015 (SR/MA) | 14918 participants diagnosed with BD I or II or described as experiences mania during the follow-up | Cannabis Use | 6/ 3 | PsychINFO, Cochrane, SCOPUS, EMBASE, and Medline (Up to June 2014) | Prospective (2000-2012) | 9 |
| Hu et al. 2020 (MA) | 6605 participants in total  Individuals diagnosed with BD or at-risk for BD | Aberrancies in White Matter | 57/ 1 | PubMed, EMBASE, and Web of Science (Up to April 23) | Cross-sectional and prospective (2008-2020) | 7 |
| Keramatian et al., (2022) | 7969 participants in total  Individuals who are at genetic and/or clinical high risk for BD | Predictors of transitioning to BD | 23/23 | Ovid Medline (Up to July 16 2021) | Prospective (1985 – 2021) | 4 |
| Lau et al. 2018 (MA) | 3454 participants in total  BD high-risk offspring and siblings | Family history of BD | 17/ 7 | PsychINFO, EMBASE, Medline, and Scopus (Up to July 2015) | Cross-sectional and prospective (1988-2015) | 8 |
| Narayan et al. 2013 (SR) | 2417 participants in total  BD high-risk offspring | Family history of BD | 13/ 4 | PubMed, PsychINFO, and Medline | Cross-sectional and prospective (1975-2012) | 2 |
| Palmier-Claus et al. 2016 (SR/MA) | 2102377 participants in total  Individuals with a formal diagnosis of BD (including prospective studies reporting first-onset mania as an outcome) | Childhood Adversity | 11/ 1 | Medline, EMBASE, PsychINFO, and Web of Science (NR) | Case-control, cross-sectional and prospective (1980- 2014) | 9 |
| Pancheri et al. 2019 (SR) | 34459 participants in total  Prospective Studies: BD high-risk individuals who are later diagnosed with BD and individuals with sleep alterations and develop full-blown BD at follow up  Retrospective studies: Individuals with a diagnosis of BD | Sleep Alterations | 17/ 6 | Medline, PubMed, Index Medicus, and Cochrane Library (Up to January 1^st^) | Prospective and retrospective (2000- 2017) | 6 |
| Rasic et al. 2014 (MA) | 3863 participants in total  Offspring of parents with severe mental illness (SMI; schizophrenia, BD, MDD) | Family history of BD | 33/ 3 | Medline, PubMed, EMBASE, PsychINFO (Up to December 31, 2012) | Cross-sectional and prospective (1989-2012) | 6 |
| Ratheesh et al. 2011 (SR/MA) | 17688 participants in total  Individuals diagnosed with MDD at intake and later converted to BD I or II at follow-up | Any factor that is related to the transition from MDD to BD | 56/ 56 | Medline, PsychINFO, and EMBASE (Up to September 2016) | Prospective (1973 – 2016) | 9 |
| Ritter at al. 2011 (SR/MA) | 85957 participants in total  Prospective Studies: Offspring of parents with BD, individuals with a diagnosis of insomnia or disturbed sleep Retrospective studies: Patients with a diagnosis of BD | Disturbed Sleep | 21/ 6 | ISI- Web of Science including, amongst others: Science Citation Index Expanded, Social Sciences Citation Index, Arts and Humanities Citation Index, Con- ference Proceedings Citation Index–Science, Con- ference Proceedings Citation Index–Social Science and Humanities, and Medline (NR) | Prospective and retrospective (1989-2008) | 2 |
| Scott et al. 2021 (SR/MA) | 58496 participants in total  Participants had a mean age ≤30 at the time of assessment of any sleep disturbances or the mean age at onset of the sleep disturbances was <30 and had first onset mental disorders which are BD, depressive disorders, and psychotic disorders at follow-up | Sleep Disturbances | 41/11 | PubMed, PsychINFO, Embase, and Web of Science | Prospective (1996-2020) | 11 |
| Scott et al. 2022 (SR/MA) | 25830 in total  Offspring of BD, subthreshold manifestations of BD assessed using BAR, full-threshold diagnostic criteria for BD-I or BD-II with first onset by about 25 years | Sleep and circadian rhythm disturbances | 76/20 | PubMed, PsychInfo, CINAHL, Embase, Web of Science | Case-control, cross-sectional, prospective | 10 |

*Note.* ADHD = Attention Deficit Hyperactivity Disorder, BAR = youth with bipolar at-risk states; BD = Bipolar Disorder; MA = meta-analysis; MDE = Major Depressive Episodes; MDD = Major Depressive Disorder; N = Number of included studies; n = number of relevant prospective studies; NOS = Not Otherwise Specified; NR = Not reported; SMI = Severe Mental Illness; SR = Systematic Review, SUD = Substance Use Disorders.

**Table S2**

Characteristics of systematic reviews and meta-analyses examining the developmental pathways and onset of Borderline Personality Disorder

| Study (Type) | Sample Size and Characteristics | Factors examined | N/n | Databases searched (last assessed) | Types of studies included (publication date range) | AMSTAR index |
| --- | --- | --- | --- | --- | --- | --- |
| Skabeikyte & Barkauskiene 2021 (SR) | 16157 adolescents aged ten to eighteen years old who showed BPD features or symptoms or had diagnosis at the follow-up period | Any predictor (i.e., putative risk factor) associated with BPD features, symptoms, or diagnosis | 14/14 | Medline, PubMed, PsychINFO, PsychARTICLES, SocINDEX, Proquest and Scopus (NR) | Prospective (2013-2020 | 9 |
| Stepp et al. 2016 (SR) | 43681 high-risk, community or clinical samples who had BPD symptoms, features or diagnosis at follow-up assessment | Any predictor (i.e., putative risk factor) associated with BPD features, symptoms, or diagnosis | 39/39 | PubMed, CINAHL, PsychINFO, ISI Web of Science (Up to September 15) | Prospective (1993-2015) | 5 |
| Winsper et al. 2016a (SR) | 6313 participants nineteen years of age or younger who were diagnosed with BPD or showed BPD symptoms as an outcome | Neurobiological correlates (i.e., genetic, neurophysiological, structural brain characteristics, neuropsychological) of BPD | 34/2 | Medline, EMBASE, PsychINFO, and PubMed (Up to 28^th^ January 2014) | Cross-sectional and prospective (1999-2016) | 8 |
| Winsper et al. 2016b (SR/MA) | 29395 participants nineteen years of age or under and have a diagnosis of BPD or have BPD features | Psychopathological (i.e., psychiatric disorders and suicidality) and etiological (i.e., adverse life events) factors | 61/9 | Medline, EMBASE, PsychINFO, PubMed | Cross-sectional, retrospective and prospective (1985-2015) | 10 |
| Winsper et al. 2017 (SR/MA) | 29860 participants with BPD or have BPD symptoms | Sleep profile (i.e., continuity, architecture, and nightmares) | 32/2 | EMBASE, PsychINFO, and PubMed (Up to December 2015) | Cross-sectional and prospective (1983-2016) | 10 |

*Note.* MA = meta-analysis; N = Number of included studies; n = number of relevant prospective studies; NR = Not reported; SR = Systematic Review

**Table S3**

Evidence across systematic reviews and meta-analyses of factors for Bipolar Disorder outcome

| Study (Sample) | Factor Examined | k | Main Findings |
| --- | --- | --- | --- |
| Vulnerability Factors |  |  |  |
| Ratheesh et al. 2017 (Individuals diagnosed with MDD at intake and later converted to BD I or II at follow-up) | Gender | 10 | Out of ten studies only one study found a significant association between male gender and transition to BD |
| Lau et al. 2018 (BD high-risk offspring and siblings) | Family history of BD | 7 | All seven studies reported at least one mental illness diagnosis in high-risk offspring including anxiety disorders, problems in psychosocial functioning, internalising and externalising behaviours, MDD, sleep, SUD, schizoaffective disorder. Only two of them found life-time prevalence of BD in the high-risk offspring |
| Keramatian et al. 2022 (Individuals who are at clinical or genetic risk of developing BD) | Family History of BD | 5 | The study found that most offspring of BD parents did not develop BD, but they were at specific high risk for developing BD, particularly those with preschool ADHD and early-onset parental BD. The second study also found that earlier parental age at onset individually and collectively predicted new onset BD. Third study also found that offspring of parents with early onset BD entailed a higher risk of BD and substance use disorders than those with later onset and controls. Forth study found that more than half of the Dutch BD offspring cohort had developed a mood disorder including 13% with bipolar spectrum disorders (3% with bipolar I disorder; 8% with bipolar II disorder; 1% with schizoaffective disorder, bipolar type; and 1% with cyclothymia) and 41% with a unipolar depressive disorder (major depressive disorder; dysthymia; depressive disorder not otherwise specified; or adjustment disorder, mood). Fifth study found that familial hypomania/mani predicted conversion. |
| Narayan et al. 2013 (BD high-risk offspring) | Family History of BD | 7 | Six studies reported at least one mental illness diagnosis including schizoaffective disorder, thought problems and psychosis-NOS.  Two studies did not find significant group differences between offspring risk groups for cluster A personality disorders |
| Pancheri et al. 2019 (BD Offspring) | Family history of BD and sleep disorders | 4 | First study found that 50% of children later diagnosed with BD presented decreased sleep as an antecedent. Second study also found that high-risk offspring has a higher risk of sleep disorders and the sleep difficulties started about 6 years before the onset of the first major mood episode. Third study found that bipolar offspring youth with poor sleep were 4 times more likely to develop BD and sleep symptoms presented about 3 years before BD onset, but the results were not significant. The fourth study found that in the BD offspring group who converted to BD with a hypomanic onset, decreased need of sleep and middle insomnia were significantly associated with BD conversion. |
| Rasic et al. 2014 (Offspring of individuals with SMI) | Family history of BD | 3 | First study found that offspring of BD developed major mood episodes in adolescence and not before and concluding that adolescence marks the beginning of the high-risk period for major mood episodes related to BD onset. Second study also found that the lifetime prevalence of mood disorders in general and BD in particular increased from 3 to 10% at follow-up among children of BD parents. The third study found that 24% of the offspring of BD parents received a positive diagnosis clustered in the affective illness spectrum. |
| Ratheesh et al. 2017 (Individuals diagnosed with MDD at intake and later converted to BD I or II at follow-up) | Family history of BD | 8 | Five study out of eight identified a positive association between family history of BD and later BD in the offspring |
| Ratheesh et al. 2017 (Individuals diagnosed with MDD at intake and later converted to BD I or II at follow-up) | Family history of Affective Disorder | 4 | Out of 4 studies only one study identified a significant association between family history of AD and later BD |
| Ratheesh et al. 2017 (Individuals diagnosed with MDD at intake and later converted to BD I or II at follow-up) | Family History of depression | 3 | Only one study found significant associations between having first degree relatives with MDD and later BD |
| Ratheesh et al. 2017 (Individuals diagnosed with MDD at intake and later converted to BD I or II at follow-up) | Other familial history predictors | 2 | Two studies found significant associations between risk of developing BD and either family history of AD spanning three generations or multiple affected family members |
| Ritter et al. 2013 (BD Offspring) | Family history of BD and sleep disturbances in the offspring | 6 | Two studies following the same cohort of high-risk offspring found that antecedent conditions to BD included sleep and anxiety disorders. Two other studies again investigating early symptoms in the same high-risk cohort first found that 14% of high-risk children had either hypersomnia or unspecified sleep problems but results were not significant. Later in the updated publication of the cohort, decreased sleep problems reached statistical significance among at-risk adolescents. Last two studies following the same cohort, sleep disturbances were not found to be a significant predictor although the measure were not reported in the article itself (the author of this review got in contact with the authors) |
| Keramatian et al. 2022 (Individuals who are at clinical or genetic risk of developing BD) | Psychosocial functioning | 1 | The study found that poor general psychosocial functioning predicted new onset of BD |
| Palmier-Claus et al. 2016 (Individuals with a formal diagnosis of BD (including prospective studies reporting first-onset mania as an outcome) | Childhood Adversity | 1 | The study found that childhood physical, verbal, sexual abuse and neglect were significantly associated with higher risks of both first-onset and recurrent mania |
| Ratheesh et al. 2017 (Individuals diagnosed with MDD at intake and later converted to BD I or II at follow-up) | Childhood Adversity | 2 | One study found child abuse and the other study found severity of childhood trauma were associated with later BD |
| Biomarkers |  |  |  |
| Bart et al. 2021 (Individuals at familial risk for BD) | Neural reward circuit dysfunction | 2 | One study found that; BD offspring had lower connectivity between the ventral striatum (VS) and left central anterior cingulate cortex during loss trials compared to not BD (nBD) offspring and control offspring, they had greater connectivity between the pars orbitalis and orbitofrontal cortex (OFC) during reward trials compared to not nBD offspring, nBD offspring had lower connectivity between pars orbitalis and right OFC during reward trials compared to control offspring, BD offspring had greater connectivity between the pars triangularis and right OFC during loss trials compared to control offspring. The other study found that lower bilateral parietal cortical thickness, greater left ventrolateral prefrontal cortex thickness, lower right transverse temporal cortex thickness, greater self-reported depression, mania severity, and age at scan predicted greater future mixed/mania factor score and lower bilateral parietal cortical thickness, greater right entorhinal cortical thickness, greater right fusiform gyral activity during emotional face processing, diagnosis of major depressive disorder, and greater self-reported depression severity predicted greater irritability factor score. |
| Cahn et al. 2021 (Individuals with first episode mania) | Longitudinal grey matter changes | 1 | The study found that adolescents with mania failed to exhibit normal increases in amygdala volume that occur during healthy adolescent neurodevelopment |
| Hu et al. 2020 (Individuals at familial risk for BD) | Aberrancies in White Matter | 1 | The study found that fractional anisotropy reduction did not differ significantly between high familial risk individuals for BD and controls |
| Clinical Factors/Features |  |  |  |
| Cardoso et al. 2018 (Individuals who experienced at least one depressive episode with no history of hypomanic or manic episode) | Suicidality | 3 | Out of three studies, one found suicide risk (i.e., recurrent thoughts of death, recurrent suicidal ideation, or a suicide attempt or a specific plan for committing suicide) as a predictor of diagnosis conversion to BD  One study found significant results for alcohol use or SUD at baseline, family history of mood disorders and failure to respond to antidepressant treatments were associated with development of BD  Third study also found that alcohol use disorder and family history of SUD were predictors of BD conversion |
| Ratheesh et al. 2017 (Individuals diagnosed with MDD at intake and later converted to BD I or II at follow-up) | Suicide ideation or attempts | 4 | Three out of four studies found significant associations between suicidality and later BD |
| Faedda et al. 2015 (Participants who are diagnosed with MDE, MDD, dysthymia, cyclothymia, or bipolar NOS as well as subjects with subsyndromal affective disorders or symptoms at intake and bipolar I or bipolar II during follow-up) | Affective Instability | 3 | One study found affective instability predicted BD II but not BD I. They also found that BP II converters were distinguished from those who remained unipolar on the basis of energy activity, temperamental instability and daydreaming. BD II switchers also had a more tempestuous course with shorter intervals. Another study also found that it predicted BD II and BD spectrum disorders (MDD and subsyndromal hypomania) and having ‘ups and downs’ was an indicator of disturbed mood regulation  Another study, on the contrary, found past or current affective instability at baseline predicted conversion to BD I and Bipolar NOS in adults hospitalised for MDD with psychotic features |
| Faedda et al. 2015 (Participants who are diagnosed with MDE, MDD, dysthymia, cyclothymia, or bipolar NOS as well as subjects with subsyndromal affective disorders or symptoms at intake and bipolar I or bipolar II during follow-up) | Subsyndromal Depression | 3 | Higher rates of later conversion to BD, especially BD II in two studies, were reported in all three studies |
| Keramatian et al. 2022 (Individuals who are at clinical or genetic risk of developing BD) | Major Depressive Episodes | 1 | The study found that major depressive episodes were indicators for close monitoring of emergent BD in high-risk offspring. |
| Keramatian et al. 2022 (Individuals who are at clinical or genetic risk of developing BD) | Depression in offspring of BD | 2 | One study found that a unipolar depression in bipolar offspring is associated with the development of BD. The other study found that elevated scores on the Depression scale in General Behavior Inventory (GBI) predicted a switch from unipolar to BD. The other study found that major depressive episodes were predicted the onset of (hypo)manic episodes. |
| Faedda et al. 2015 (Participants who are diagnosed with MDE, MDD, dysthymia, cyclothymia, or bipolar NOS as well as subjects with subsyndromal affective disorders or symptoms at intake and bipolar I or bipolar II during follow-up) | Subsyndromal hypomanic symptoms | 5 | First study found higher scores on Hypomanic Personality Scale significantly predicted conversion to BD, especially BD II. Second study found the same results for BD I and Bipolar NOS. Third study found the combination of subclinical mania with subclinical psychosis at intake predicted onset of BD three times more. Fourth study found symptoms of elation or irritability, especially their combination predicted later hypomania or mania. Fifth study found number and persistence of hypomania or mania symptoms increased monotonically at follow-up before full-blown BD criteria were met |
| Keramatian et al. 2022 (Individuals who are at clinical or genetic risk of developing BD) | Subthreshold manic symptoms | 5 | Three studies found that subthreshold manic symptoms or hypomanic episodes are predictive of BD. Fourth study found that “high mania” predicted BD conversion. Fifth study found that subthreshold manic subgroup of Bipolar-At-Risk (BAR) criteria predicted conversion to BD |
| Ratheesh et al. 2017 (Individuals diagnosed with MDD at intake and later converted to BD I or II at follow-up) | Subthreshold (hypo)manic features |  | Three studies identified a significant association between subthreshold hypomanic symptoms and BD transition. Two of those studies also found a dose-response relationship between the number of manic symptoms and later BD. Two studies found an association between antidepressant associated subthreshold hypomanic episodes and transition to BD. |
| Faedda et al. 2015 (Participants who are diagnosed with MDE, MDD, dysthymia, cyclothymia, or bipolar NOS as well as subjects with subsyndromal affective disorders or symptoms at intake and bipolar I or bipolar II during follow-up) | Subsyndromal hypomanic symptoms in major depression | 5 | One study reported that hyperenergetic involvement in activities predicted conversion to BD presenting more hypomanic than manic episodes. However, one-third of those individuals with BD II later developed mania meaning that mood elevation intensifies progressively  Another study found that hypomanic symptoms, specifically decreased need for sleep, unusually high energy, increased and goal directed activity, significantly predicted both hypomania and mania. Grandiosity, on the other hand, predicted only mania  Another study also found that subsyndromal hypomania with MDD significantly increased the likelihood of conversion to BD. One study reported that subsyndromal hypomanic symptoms significantly predicted conversion to BD I and Bipolar NOS in adults with psychotic MDD. Lastly, conversion rates to BD for children with MDD and/or dysthymia presenting transient manic symptoms were significantly higher compared to the children with only MDD and/or dysthymia |
| Faedda et al. 2015 (Participants who are diagnosed with MDE, MDD, dysthymia, cyclothymia, or bipolar NOS as well as subjects with subsyndromal affective disorders or symptoms at intake and bipolar I or bipolar II during follow-up) | Cyclothymic disorder and Bipolar NOS | 5 | First study found that outpatients diagnosed with cyclothymic disorder later developed BD  Second study found that children and adolescents hospitalised with MDD and have high scores on the Cyclothymic-Hypersensitive Temperament Rating Scale significantly predicted conversion to BD. Third study found 16.5% of the youths with hypomania but without a MDD converted to BD II. Fourth study found higher conversion rates from bipolar NOS to BD I and II. Similarly, fifth study found that individuals with bipolar NOS or cyclothymic disorder, more than half of them developed BD, particularly BD II |
| Keramatian et al. 2022 (Individuals who are at clinical or genetic risk of developing BD) | Cyclothymic/irritable and hyperthymic temperament | 1 | The study found that cyclothymic/irritable and hyperthymic temperaments predicted both total cases and new cases of bipolar spectrum disorders at the follow-up. |
| Keramatian et al. 2022 (Individuals who are at clinical or genetic risk of developing BD) | Bipolar NOS | 1 | Earlier onset Bipolar NOS predicted conversion to BD. |
| Faedda et al. 2015 (Participants who are diagnosed with MDE, MDD, dysthymia, cyclothymia, or bipolar NOS as well as subjects with subsyndromal affective disorders or symptoms at intake and bipolar I or bipolar II during follow-up) | Psychotic Symptoms in Major Depression | 7 | All seven studies identified significant relationships between MDD with psychotic features and later BD both in adolescents and adults |
| Faedda et al. 2015 (Participants who are diagnosed with MDE, MDD, dysthymia, cyclothymia, or bipolar NOS as well as subjects with subsyndromal affective disorders or symptoms at intake and bipolar I or bipolar II during follow-up) | Psychotic Disorders | 3 | Two studies reported that psychosis NOS predicted later BD. Third study found that schizotypal features and schizophrenia nuclear symptoms predicted conversion to BD but lacked specificity |
| Ratheesh et al. 2017 (Individuals diagnosed with MDD at intake and later converted to BD I or II at follow-up) | Presence of psychotic symptoms | 5 | Four studies identified a significant association between psychotic symptoms and transition to BD |
| Faedda et al. 2015 (Participants who are diagnosed with MDE, MDD, dysthymia, cyclothymia, or bipolar NOS as well as subjects with subsyndromal affective disorders or symptoms at intake and bipolar I or bipolar II during follow-up) | Age at onset of Major Depression | 4 | All four studies reported early onset of depression predicted conversion to BD |
| Ratheesh et al. 2017 (Individuals diagnosed with MDD at intake and later converted to BD I or II at follow-up) | Age of onset of depression | 9 | Seven studies out of nine found a significant association between lower age of onset of depression and higher rates of transition to BD  Another finding was that age at onset of MDD in individuals who did develop BD was 4.8 years earlier than age at onset of MDD in those who did not develop BD |
| Keramatian et al. 2022 (Individuals who are at clinical or genetic risk of developing BD) | Psychomotor retardation and Mood Disorder Episodes | 1 | The study found that psychomotor retardation and mood disorder episodes predicted conversion to BD in those at high familial risk. |
| Ratheesh et al. 2017 (Individuals diagnosed with MDD at intake and later converted to BD I or II at follow-up) | Severity of depression with baseline MDD | 2 | Both studies found significant association with severity of depression and onset of BD. One of them also found significant associations for psychomotor retardation and mood congruent psychotic features. The other study found that switch from MDD to BD was also predicted by cluster B personality disorder symptoms and OCD |
| Ratheesh et al. 2017 (Individuals diagnosed with MDD at intake and later converted to BD I or II at follow-up) | Guilt with baseline MDD | 3 | Three studies found guilt coexistent with MDD was associated with transition to BD. One of them also found that converters to BD from MDD were significantly characterised also by diurnal variation and complete loss of pleasure. They did not find differences for hyperphagia, psychomotor alterations and hypersomnia |
| Keramatian et al. 2022 (Individuals who are at clinical or genetic risk of developing BD) | Mood lability | 2 | Both studies found that mood lability predicted BD conversion. |
| Ratheesh et al. 2017 (Individuals diagnosed with MDD at intake and later converted to BD I or II at follow-up) | Affective instability with baseline MDD | 2 | Affective instability with baseline MDD was associated with BD onset in both studies |
| Faedda et al. 2015 (Participants who are diagnosed with MDE, MDD, dysthymia, cyclothymia, or bipolar NOS as well as subjects with subsyndromal affective disorders or symptoms at intake and bipolar I or bipolar II during follow-up) | Frequency and loading of affective symptoms | 2 | First study found that the risk of conversion to BD increased with the number of lifetime depressive episodes and with the number of hypomanic symptoms Second study found that longer episodes of depression, greater loading of depressive symptoms, and higher recurrence rates predicted later BD |
| Ratheesh et al. 2017 (Individuals diagnosed with MDD at intake and later converted to BD I or II at follow-up) | Recurrent MDD | 6 | Four study found a significant association between MDD and later BD while the other two studies did not |
| Ratheesh et al. 2017 (Individuals diagnosed with MDD at intake and later converted to BD I or II at follow-up) | Chronicity of depression | 2 | One study found chronicity of depression episode predicted shifts from nonbipolar to bipolar II whereas the other study did not find a significant association |
| Ratheesh et al. 2017 (Individuals diagnosed with MDD at intake and later converted to BD I or II at follow-up) | ‘Hypersomnic-retarded’ depression | 6 | Only one study of six studies identified an association between ‘hypersomnic-retarded’ depression and transition to BD |
| Keramatian et al. 2022 (Individuals who are at clinical or genetic risk of developing BD) | Sleep difficulties | 3 | One study found that those in the poor sleep group had almost twice the odds of developing BD as those in the good and variable sleep group. The other study found that frequent waking during the night, insufficient sleep, and time to fall asleep significantly predicted the development of BD. Third study found that childhood sleep disorders significantly predicted 1.6 fold and 1.8 fold increases in risk of mood disorders in offspring of BD. |
| Pancheri et al. 2019 (Patients with sleep disorder/symptoms who later develop BD) | Sleep disorders | 2 | First study found that patients with insomnia treated with hypnotic drugs had a higher risk of developing BD compared to those without insomnia and with insomnia not on hypnotics. Second study reported that disturbed sleep at baseline (trouble falling asleep and early morning awakening) significantly increased the risk for the development of BD, even when they adjusted age, gender, parental mood disorder and lifetime cannabis or alcohol use |
| Scott et al. 2021 (Individuals with sleep disturbances who later develop BD, depressive disorders or psychotic disorders) | Sleep Disturbances | 11 | All eleven studies found that, individuals with a history of any type of sleep disturbance (e.g., decreased need for sleep, insomnia, hypersomnia with fatigue, inadequate sleep, frequent night time awakening, circadian disturbance) had an increased odds of BD |
| Scott et al. 2022 (Offspring of BD) | Sleep and circadian rhythm disturbances | 11 | All eleven studies reported that sleep problems were significantly more frequent in offspring of BD compared to controls. Characteristics of the sleep problems were decreased need for sleep, middle insomnia, frequent night-time awakenings, inadequate sleep, and high energy One study found that decreased need for sleep’ was associated with transition to BD as did middle insomnia. However, neither hypersomnia nor daytime fatigue showed any association with onset of any mood disorder. Another study found that parental rating of chronotype was predictive of BD onset, but OSBD self-rating of chronotype was not. Another study indicated that high-risk group showed a shift from more internalizing symptoms (including anxiety/worry & somatic complaints) in childhood to more ‘manic-like behaviours’ (including high energy, reduced need for sleep), excessive and loudness and concentration difficulties in adolescence. During school years (but not in pre-school), periodic symptoms that mainly differentiated offspring of BD-I from controls were: mood (sad), fearfulness, changes in energy levels and sleep and circadian rhythm disturbances (either decreased, difficulty falling asleep or early waking: ~23% v. 8%). |
| Scott et al. 2022 (Stage 2, full-threshold diagnostic criteria for BD-I or BD-II with first onset by about 25 years) | Sleep and circadian rhythm disturbances | 9 | All studies found that sleep and circadian rhythm disturbances were significantly higher in cohorts with first onset of BD. The predictors were trouble sleeping, low social rhythm regularity, insomnia, daytime dysfunction, hypersomnia, anergia, circadian disturbance, decreased REM sleep, hypersomnia with fatigue, and low regularity. |
| Keramatian et al. 2022 (Individuals who are at clinical or genetic risk of developing BD) | Antidepressant Use | 1 | The study found that exposure to antidepressants during follow-up was associated with increased risk of conversion |
| Ratheesh et al. 2017 (Individuals diagnosed with MDD at intake and later converted to BD I or II at follow-up) | Antidepressant Use | 4 | None of the studies found a significant association between antidepressant use and later BD |
| Keramatian et al. 2022 (Individuals who are at clinical or genetic risk of developing BD) | Disruptive Behaviour Disorders | 1 | The study found that disruptive behaviour disorders were associated with subsequent manic, mixed, or hypomanic episodes. |
| Keramatian et al. 2022 (Individuals who are at clinical or genetic risk of developing BD) | Baseline anxiety disorders and subsyndromal mood disorders | 4 | The study found that baseline anxiety disorders and depressive disorders NOS were associated with increased risk of conversion during follow-up. ADHD and disruptive disorders were not associated with conversion. Second study also found that anxiety and depression predicted BD. Third study found that childhood anxiety disorders significantly predicted conversion to BD. Fourth study found that anxiety predicted conversion to BD in youth with bipolar NOS. |
| Keramatian et al. 2022 (Individuals who are at clinical or genetic risk of developing BD) | Temperament | 1 | The study found that key symptoms to identify children with BD from well children in cohort samples were sensitivity, crying, hyper alertness, anxiety/worry, somatic complaints, bold/intrusive, excessive talk, talk too loudly, easily excited, poor attention, decreased sleep, and impaired role in school. |
| Ratheesh et al. 2017 (Individuals diagnosed with MDD at intake and later converted to BD I or II at follow-up) | Comorbid anxiety disorder as a group | 2 | One study found comorbid anxiety disorder predicted the onset of manic symptoms whereas the other study did not find significant relationships with comorbid anxiety disorder and later BD |
| Ratheesh et al. 2017 (Individuals diagnosed with MDD at intake and later converted to BD I or II at follow-up) | Comorbid GAD | 2 | One study found significant associations between GAD and later BD while the other one did not find |
| Gibbs et al. 2015 (Participants described as experiences mania during the follow-up) | Cannabis Use | 3 | All three studies found that baseline cannabis use significantly predicted hypo/sub-threshold mania symptoms at follow-up |
| Keramatian et al. 2022 (Individuals who are at clinical or genetic risk of developing BD) | Drug Use Disorder | 1 | The study found that drug use disorders predicted (hypo)manic episodes. |
| Ratheesh et al. 2017 (Individuals diagnosed with MDD at intake and later converted to BD I or II at follow-up) | Comorbid SUD | 4 | Two studies found significant associations between substance use disorders and later BD while the other 2 did not find a significant association |
| Ratheesh et al. 2017 (Individuals diagnosed with MDD at intake and later converted to BD I or II at follow-up) | Comorbid SP | 2 | Both studies found an association between SP and later BD |
| Brancati et al. 2021 (Individuals with ADHD who later developed BD) | Comorbid ADHD | 10 | All ten studies showed a significantly greater risk of BD occurrence in ADHD patients versus healthy controls |
| Ratheesh et al. 2017 (Individuals diagnosed with MDD at intake and later converted to BD I or II at follow-up) | Comorbid ADHD | 1 | The study found that ADHD was significantly associated with a higher risk for conversion from unipolar to BD. The switches were predicted by presence of parental mood disorder, school behaviour problems, and baseline comorbid conduct disorder |

*Note.* ADHD = Attention Deficit Hyperactivity Disorder; BD = Bipolar Disorder; GAD = Generalised Anxiety Disorder; k = number of prospective studies for each factor; OCD = Obsessive Compulsive Disorder; MDD = Major Depressive Disorder; NOS = Not Otherwise Specified; NR = Not Reported; SMI = Severe Mental Illness; SP = Social Phobia; SUD = Substance Use Disorder

**Table S4**

Evidence across systematic reviews and meta-analyses of factors for Borderline Personality Disorder outcome

| Study (Sample) | Factor Examined | k | Main Findings |
| --- | --- | --- | --- |
| Environmental Factors |  |  |  |
| Winsper et al. 2016a (Participants nineteen years of age or younger who were diagnosed with BPD or showed BPD symptoms as an outcome) | Heritability of BPD | 2 | First study found that monozygotic (MZ) correlations were higher than dizygotic (DZ) correlations and heritability co-efficient BPD symptoms ranged from 0.3 to 0.5. The results of the study also indicated that BPD traits decline as individuals mature from adolescence to adulthood and trait BPD is highly influenced by genetic factors and modestly by non-shared environmental factors. Second study also identified that there was a higher correlation of BPD symptoms between MZ than DZ twins and genetic factors accounted for 66% of the variance in BPD symptoms and BPD symptoms measured at twelve years were highly heritable and were preceded by behavioural and affective dysregulation and poor cognitive function |
| Psychosocial Factors |  |  |  |
| Stepp, Lazarus, & Byrd 2016 (High-risk, community or clinical samples who had BPD symptoms, features or diagnosis at follow-up assessment) | Low SES | 4 | All four studies found a prospective association with later BPD symptoms. |
| Stepp, Lazarus, & Byrd 2016 (High-risk, community or clinical samples who had BPD symptoms, features or diagnosis at follow-up assessment) | Stressful life events | 3 | Two studies identified that stressful life events (i.e., various psychosocial stressors) predicted BPD symptoms in adults. Chronic and school stressors in adolescence were also associated with BPD symptoms in adulthood. Only one study did not find a link between stressful life events and a BPD diagnosis at the age of fifteen. However, Stepp et al. (2016) notes this might be due to differences in sampling strategies |
| Skabeikyte & Barkauskiene 2021 (Adolescents aged ten to eighteen years old who showed BPD features or symptoms or had diagnosis at the follow-up period) | Stressful Life Events | 2 | One study looking at stressful life events (e.g., suspension from school, death of a parent, changes in peer acceptance) at ages twelve to seventeen did not find significant associations. The other study looking at the link between academic functioning at age eight and later BPD features did not find significant predictive associations |
| Stepp, Lazarus, & Byrd 2016 (High-risk, community or clinical samples who had BPD symptoms, features or diagnosis at follow-up assessment) | Family Adversity | 4 | Two studies found that family adversity during pregnancy and across childhood and adolescence predicted BPD symptoms. Two other studies looking at family disruption and marital conflict did not find significant associations with later BPD outcomes |
| Stepp, Lazarus, & Byrd 2016 (High-risk, community or clinical samples who had BPD symptoms, features or diagnosis at follow-up assessment) | Maternal Psychopathology | 6 | All six studies identified a significant association with maternal psychopathology (i.e., internalising and externalising disorders) and offspring BPD |
| Stepp, Lazarus, & Byrd 2016 (High-risk, community or clinical samples who had BPD symptoms, features or diagnosis at follow-up assessment) | Maternal BPD symptoms | 1 | The study reported an indirect effect of maternal BPD symptoms on offspring BPD symptoms via maladaptive parenting (especially an overprotective and rejecting parenting style and high discrepancies in internalising problems) behaviours.  The results were consistent for various offspring symptoms which were associated with BPD such as impulsivity and dissociation |
| Stepp, Lazarus, & Byrd 2016 (High-risk, community or clinical samples who had BPD symptoms, features or diagnosis at follow-up assessment) | Paternal Substance Abuse | 1 | The study found that paternal substance use predicted later BPD symptoms in the offspring |
| Stepp, Lazarus, & Byrd 2016 (High-risk, community or clinical samples who had BPD symptoms, features or diagnosis at follow-up assessment) | Parenting behaviour/style | 12 | Seven studies identified significant associations with affective parenting dimensions (i.e., low warmth, rejection, low maternal satisfaction with the child, hostility, harsh punishment/discipline) and higher BPD symptoms. Additionally, one study found reciprocal association between harsh punishment, low warmth and BPD symptoms across adolescence, meaning that low warmth and harsh punishment predicted higher BPD symptoms and in turn BPD symptoms predicted increased parental harsh punishment and low warmth. Only two studies did not find predictive associations with harsh discipline and low affection. One study found that disrupted maternal communication at eighteen months predicted BPD symptoms at age eighteen. Similarly, another study reported predictive association with maternal hostility at 42 months and BPD symptoms at age twenty-eight. One other study identified that maternal expressed negative emotion in middle childhood significantly predicted BPD features at age 12. Two other studies looked at the influence of behavioural control dimensions of parenting and only one identified higher levels of maternal inconsistency and over-involvement predicted BPD diagnosis at age 16. The other study did not find significant associations between inconsistent parenting and later BPD symptoms. Lastly, one study found significant associations between poor parenting (i.e., behavioural and affective dimensions) and BPD symptoms in adolescence and adulthood |
| Skabeikyte & Barkauskiene 2021 (Adolescents aged ten to eighteen years old who showed BPD features or symptoms or had diagnosis at the follow-up period) | Parenting Style | 4 | First study found that parental low warmth and changes in parental harsh punishment were not predictive of changes in BPD features. Second study also found the non-significant results for parental harsh punishment. Third study looking at maternal support/validation also did not find predictive associations with changes in BPD features. Fourth study found that exposure to intimate partner violence among parents was predictive of slower declines in BPD symptoms throughout adolescence |
| Stepp, Lazarus, & Byrd 2016 (High-risk, community or clinical samples who had BPD symptoms, features or diagnosis at follow-up assessment) | Parent-child relationship quality | 5 | Only two studies identified significant prospective associations between parent-child relationship and later BPD. One study reported that mother-child discord in adolescence predicted BPD symptoms at age thirty. The other study found that family relationship quality predicted BPD symptoms for those with the oxytocin receptor gene variation risk genotype |
| Stepp, Lazarus, & Byrd 2016 (High-risk, community or clinical samples who had BPD symptoms, features or diagnosis at follow-up assessment) | Family History of Psychiatric Hospitalisation | 1 | The study identified that history of psychiatric hospitalisation interacted with maltreatment and maternal negative emotion predicted later BPD symptoms |
| Stepp, Lazarus, & Byrd 2016 (High-risk, community or clinical samples who had BPD symptoms, features or diagnosis at follow-up assessment) | Maternal Characteristics | 3 | Three studies looking at maternal ego integration, impulsivity, interpersonal difficulties, and history of serious medical problems were not associated with later BPD |
| Skabeikyte & Barkauskiene 2021 (Adolescents aged ten to eighteen years old who showed BPD features or symptoms or had diagnosis at the follow-up period) | Maternal Characteristics | 2 | Both studies found that maternal BPD symptoms and maternal depression were predictive of higher mean BPD levels in the offspring |
| Skabeikyte & Barkauskiene 2021 (Adolescents aged ten to eighteen years old who showed BPD features or symptoms or had diagnosis at the follow-up period) | Parental Depression Severity | 2 | Both studies did not find significant association between parental depression severity and changes in BPD symptoms |
| Stepp, Lazarus, & Byrd 2016 (High-risk, community or clinical samples who had BPD symptoms, features or diagnosis at follow-up assessment) | Childhood Abuse | 8 | Four studies found significant associations with physical abuse, one study found with verbal abuse, one another study found with emotional abuse and four studies found with sexual abuse and later BPD symptoms. Three studies looked at maltreatment as a composite of types of abuse and neglect and all three of them found significant associations with BPD outcomes. Two studies did not find a significant association between physical and sexual abuse and BPD outcomes. Additionally, three other studies examining combined indices of maltreatment and other trauma did not report significant findings. One study, on the other hand, found a significant association between cumulative trauma (e.g., family suicide, death of a parent, parent arrest or imprisonment) and later BPD symptoms |
| Stepp, Lazarus, & Byrd 2016 (High-risk, community or clinical samples who had BPD symptoms, features or diagnosis at follow-up assessment) | Childhood Neglect | 3 | All three studies reported significant links between BPD and childhood neglect including neglect in general, early maternal separation, inadequate supervision and poor parental care |
| Stepp, Lazarus, & Byrd 2016 (High-risk, community or clinical samples who had BPD symptoms, features or diagnosis at follow-up assessment) | Peer Victimisation | 1 | The study reported that chronicity and severity of bullying in late childhood was significantly associated with BPD symptoms at age twelve |
| Skabeikyte & Barkauskiene 2021 (Adolescents aged ten to eighteen years old who showed BPD features or symptoms or had diagnosis at the follow-up period) | Relational Aggression | 1 | The study found that relational aggression in the context of friendship predicted higher levels of BPD symptoms |
| Skabeikyte & Barkauskiene 2021 (Adolescents aged ten to eighteen years old who showed BPD features or symptoms or had diagnosis at the follow-up period) | Psychological and Sexual Violence | 2 | First study found that psychological and sexual violence predicted increases in the average levels of later BPD features. Second study found the same findings for perceived support and antagonism in romantic relationships |
| Skabeikyte & Barkauskiene 2021 (Adolescents aged ten to eighteen years old who showed BPD features or symptoms or had diagnosis at the follow-up period) | Physical and Verbal Aggression | 1 | The study found that physical and verbal aggression experienced in romantic relationships did not predict later BPD features |
| Skabeikyte & Barkauskiene 2021 (Adolescents aged ten to eighteen years old who showed BPD features or symptoms or had diagnosis at the follow-up period) | Relationship quality with the father | 1 | The study found that relationship quality with the father predicted slower declines in BPD features during adolescence |
| Skabeikyte & Barkauskiene 2021 (Adolescents aged ten to eighteen years old who showed BPD features or symptoms or had diagnosis at the follow-up period) | Family relations and social support | 2 | Two studies failed to find significant associations between family relations, social support from friends and family and changes in BPD symptoms |
| Winsper et al. 2016b (Participants nineteen years of age or under and have a diagnosis of BPD or have BPD features) | Physical Abuse | 1 | The study found significant associations between physical abuse and later BPD symptoms |
| Winsper et al. 2016b (Participants nineteen years of age or under and have a diagnosis of BPD or have BPD features) | Parental hostility/ verbal abuse | 3 | All three studies found that maternal hostility or verbal abuse increased the risk of developing BPD |
| Winsper et al. 2016b (Participants nineteen years of age or under and have a diagnosis of BPD or have BPD features) | Parental conflict | 1 | The study found that parental conflict was significantly associated with increased odds of BPD symptoms |
| Clinical Factors |  |  |  |
| Stepp, Lazarus, & Byrd 2016 (High-risk, community or clinical samples who had BPD symptoms, features or diagnosis at follow-up assessment) | Cognitive Function | 4 | All four studies identified significant associations with low IQ and BPD symptoms |
| Stepp, Lazarus, & Byrd 2016 (High-risk, community or clinical samples who had BPD symptoms, features or diagnosis at follow-up assessment) | Attachment | 3 | First study found that disorganised/controlling behaviour at age eight predicted BPD symptoms at age nineteen but not attachment disorganisation and security. Second study found the same non-significant results for attachment disorganisation and security in infancy and toddlerhood and later BPD symptoms in adulthood. Third study reported that insecure attachment in peer relationships at age sixteen predicted BPD symptoms across adolescence and adulthood |
| Stepp, Lazarus, & Byrd 2016 (High-risk, community or clinical samples who had BPD symptoms, features or diagnosis at follow-up assessment) | Negative Affectivity and Impulsivity | 11 | All eleven studies found significant association between negative affectivity (e.g., affective instability, emotionality, aggressiveness/tantrums), impulsivity (e.g., low self-control, effortful control, low constraint) and BPD symptoms in adolescence |
| Skabeikyte & Barkauskiene 2021 (Adolescents aged ten to eighteen years old who showed BPD features or symptoms or had diagnosis at the follow-up period) | Temperament Dimensions | 1 | The study reported that high levels of emotionality, activity and low levels of sociability and shyness in middle childhood were predictive of higher and increases in average levels of BPD features through adolescence |
| Skabeikyte & Barkauskiene 2021 (Adolescents aged ten to eighteen years old who showed BPD features or symptoms or had diagnosis at the follow-up period) | Negative Affectivity | 3 | Two study examining the same cohort identified that negative affectivity in early and middle adolescence predicted only higher mean levels of BPD features but change in these features over time. Third study also looking at the same cohort found that the association between higher mean levels of BPD features from middle adolescence and negative affectivity in early adolescence was mediated by decreases in self-control skills |
| Stepp, Lazarus, & Byrd 2016 (High-risk, community or clinical samples who had BPD symptoms, features or diagnosis at follow-up assessment) | Poor self-control | 1 | The study found that poor self-control predicted later BPD symptoms via reciprocal effects between poor self-control and parental harsh discipline |
| Stepp, Lazarus, & Byrd 2016 (High-risk, community or clinical samples who had BPD symptoms, features or diagnosis at follow-up assessment) | Experiential Avoidance | 1 | The study reported that a higher level of experiential avoidance increased BPD features after a year |
| Stepp, Lazarus, & Byrd 2016 (High-risk, community or clinical samples who had BPD symptoms, features or diagnosis at follow-up assessment) | Disturbances in self representation | 1 | The study found predictive association between disturbances in self representation and BPD symptoms at age twenty-eight |
| Stepp, Lazarus, & Byrd 2016 (High-risk, community or clinical samples who had BPD symptoms, features or diagnosis at follow-up assessment) | Internalising psychopathology | 10 | Nine studies out of ten found significant prospective relationships between internalising psychopathology (i.e., suicidality, dissociation, anxiety, depression, psychosis symptoms) and later BPD symptoms |
| Stepp, Lazarus, & Byrd 2016 (High-risk, community or clinical samples who had BPD symptoms, features or diagnosis at follow-up assessment) | Externalising psychopathology | 14 | Twelve studies found significant predictive associations with externalising psychopathology (i.e., substance abuse, ADHD, oppositional defiant disorder, conduct disorder) and later BPD outcomes. Two study did not find prospective associations with externalising disorders and BPD |
| Skabeikyte & Barkauskiene 2021 (Adolescents aged ten to eighteen years old who showed BPD features or symptoms or had diagnosis at the follow-up period) | Childhood and Adolescence Psychopathology | 8 | One study found that childhood inattention, oppositional behaviour, depression severity, hyperactivity/impulsivity predicted the new onset of BPD. Another study also found that impulsivity/hyperactivity predicted higher levels of BPD symptoms throughout adolescence. SUD, MDD, anxiety symptoms, ADHD and somatisation were predictive of changes in BPD features during adolescence. Another study reported that individual social and physical aggression in childhood did not predict BPD symptoms change from fourteen to eighteen years of age. Another study found that comorbidity and decreases in depression severity were significantly related to faster declines in mean levels of later BPD symptoms. Last study reported that lower levels of a child’s general psychosocial functioning was significantly predictive of BPD diagnosis at follow-up |
| Stepp, Lazarus, & Byrd 2016 (High-risk, community or clinical samples who had BPD symptoms, features or diagnosis at follow-up assessment) | Comorbid OCD | 1 | The study did not detect a significant relationship between OCD and BPD diagnosis |
| Stepp, Lazarus, & Byrd 2016 (High-risk, community or clinical samples who had BPD symptoms, features or diagnosis at follow-up assessment) | Comorbid DSM-IV Axis 1 disorders | 2 | First study did not find a significant association between DSM-IV Axis I disorders at eight years and later BPD symptoms at age twelve. Second study using the same community sample identified significant associations between any childhood disorder and later BPD symptoms |
| Stepp, Lazarus, & Byrd 2016 (High-risk, community or clinical samples who had BPD symptoms, features or diagnosis at follow-up assessment) | Infant Characteristics | 1 | The study found that infant anomalies at birth and overall non-optimal functioning were not significantly associated with BPD symptoms in adulthood |
| Winsper et al. 2016b (Participants nineteen years of age or under and have a diagnosis of BPD or have BPD features) | Comorbid Depression | 6 | All six studies found significant associations between comorbid depression and BPD symptoms |
| Winsper et al. 2016b (Participants nineteen years of age or under and have a diagnosis of BPD or have BPD features) | Comorbid Substance Abuse | 1 | The study found that BPD traits and substance abuse are correlates rather than causal antecedents of each other and their association might rather be related to behavioural disinhibition |
| Winsper et al. 2016b (Participants nineteen years of age or under and have a diagnosis of BPD or have BPD features) | Suicidality | 2 | One study found that suicidal ideation in adolescents who were later diagnosed with BPD was not stable after posthospitalisation whereas the other study identified increased risk of self-harm in participants with borderline personality characteristics |
| Winsper et al. 2017 (Participants with BPD or have BPD symptoms) | Sleep Disturbances | 2 | First study reported that chronic nightmare significantly predicted BPD. They also found that the association was significantly mediated by emotional and behavioural problems at 9.5 years of age. Persistent sleep-onset and maintenance problems were not significantly associated with later BPD symptoms. Second study found that chronic sleep disturbances (difficulty initiating sleep, difficulty maintaining sleep, and waking earlier than desired) were significantly associated with later BPD symptoms |

*Note.* ADHD = Attention deficit hyperactivity disorder; BPD = Borderline Personality Disorder; k = number of prospective studies for each factor; MDD = Major Depressive Disorder; OCD = Obsessive compulsive Disorder; SES = Socioeconomic status; SUD = Substance Use Disorder

**Table S5.** Excluded studies after full-text review with reasons.

| ***Study Name*** | ***Include*** | ***Exclude*** | ***Excluding Reason*** |
| --- | --- | --- | --- |
| Akingbuwa WA, Hammerschlag AR, Jami ES, Allegrini AG, Karhunen V, Sallis H, Ask H, Askeland RB, Baselmans B, Diemer E, Hagenbeek FA. Genetic associations between childhood psychopathology and adult depression and associated traits in 42 998 individuals: a meta-analysis. JAMA psychiatry. 2020 Jul 1;77(7):715-28. |  | Exclude | Genetic study |
| Almeida J, Wegbreit E, Cushman G, Weissman A, Kim K, Laird A, Dickstein D. Fronto-Amygdalar Alterations During Emotional Face Processing May Differentiate Children with Bipolar Disorder from those with Major Depressive Disorder: A Functional Neuroimaging Meta-Analysis. In NEUROPSYCHOPHARMACOLOGY 2014 Dec 1 (Vol. 39, pp. S219-S219). MACMILLAN BUILDING, 4 CRINAN ST, LONDON N1 9XW, ENGLAND: NATURE PUBLISHING GROUP. |  | Exclude | Abstract for a conference poster and was never published in full text form. |
| Álvarez-Tomás I, Ruiz J, Guilera G, Bados A. Long-term clinical and functional course of borderline personality disorder: A meta-analysis of prospective studies. European Psychiatry. 2019 Feb;56(1):75-83. |  | Exclude | Intervention Study + clinical populations (full course of disorder) |
| Benarous X, Consoli A, Milhiet V, Cohen D. Early interventions for youths at high risk for bipolar disorder: a developmental approach. European Child & Adolescent Psychiatry. 2016 Mar;25(3):217-33. |  | Exclude | Literature Review |
| Carvalho AF, McIntyre RS, Dimelis D, Gonda X, Berk M, Nunes-Neto PR, Cha DS, Hyphantis TN, Angst J, Fountoulakis KN. Predominant polarity as a course specifier for bipolar disorder: a systematic review. Journal of affective disorders. 2014 Jul 1;163:56-64. |  | Exclude | Patients are already diagnosed with BD - Diagnosis does not meet inclusion standards |
| Cerimele JM, Katon WJ. Associations between health risk behaviors and symptoms of schizophrenia and bipolar disorder: a systematic review. General hospital psychiatry. 2013 Jan 1;35(1):16-22. |  | EXCLUDE | association studies - irrelevant outcome |
| Coleman JR, Gaspar HA, Bryois J, Byrne EM, Forstner AJ, Holmans PA, de Leeuw CA, Mattheisen M, McQuillin A, Pavlides JM, Pers TH. The genetics of the mood disorder spectrum: genome-wide association analyses of more than 185,000 cases and 439,000 controls. Biological psychiatry. 2020 Jul 15;88(2):169-84. |  | Exclude | GWAS study + not a systematic review |
| Craney JL, Geller B. A prepubertal and early adolescent bipolar disorder‐I phenotype: review of phenomenology and longitudinal course. Bipolar disorders. 2003 Aug;5(4):243-56. |  | Exclude | Not a systematic review- a literature review |
| Daglas R, Yücel M, Cotton S, Allott K, Hetrick S, Berk M. Cognitive impairment in first-episode mania: a systematic review of the evidence in the acute and remission phases of the illness. International journal of bipolar disorders. 2015 Dec;3(1):1-8. |  | Exclude | No longitudinal studies |
| Duarte D, Belzeaux R, Etain B, Greenway KT, Rancourt E, Correa H, Turecki G, Richard-Devantoy S. Childhood-maltreatment subtypes in bipolar patients with suicidal behavior: systematic review and meta-analysis. Brazilian Journal of Psychiatry. 2020 Jun 8;42:558-67. |  | Exclude | Irrelevant study design (not longitudinal) + patients have already been diagnosed |
| Duffy A. The nature of the association between childhood ADHD and the development of bipolar disorder: a review of prospective high-risk studies. American Journal of Psychiatry. 2012 Dec;169(12):1247-55. |  | Exclude | Not an SR |
| Duffy A, Vandeleur C, Heffer N, Preisig M. The clinical trajectory of emerging bipolar disorder among the high-risk offspring of bipolar parents: current understanding and future considerations. International journal of bipolar disorders. 2017 Dec;5(1):1-1. |  | Exclude | Not an SR |
| Duko B, Ayano G, Pereira G, Betts K, Alati R. Prenatal tobacco use and the risk of mood disorders in offspring: a systematic review and meta-analysis. Social Psychiatry and Psychiatric Epidemiology. 2020 Dec;55(12):1549-62. |  | Exclude | Irrelevant outcome - only risk factors, not related to the onset |
| Elias LR, Miskowiak KW, Vale AM, Köhler CA, Kjærstad HL, Stubbs B, Kessing LV, Vieta E, Maes M, Goldstein BI, Carvalho AF. Cognitive impairment in euthymic pediatric bipolar disorder: a systematic review and meta-analysis. Journal of the American Academy of Child & Adolescent Psychiatry. 2017 Apr 1;56(4):286-96. |  | Exclude | Patients are already diagnosed, treated with medications |
| Estrada‐Prat X, Van Meter AR, Camprodon‐Rosanas E, Batlle‐Vila S, Goldstein BI, Birmaher B. Childhood factors associated with increased risk for mood episode recurrences in bipolar disorder—A systematic review. Bipolar disorders. 2019 Sep;21(6):483-502. |  | Exclude | Patients are already diagnosed - full blown bipolar and mood recurrences - irrelevant outcome |
| de Aquino Ferreira LF, Pereira FH, Benevides AM, Melo MC. Borderline personality disorder and sexual abuse: a systematic review. Psychiatry research. 2018 Apr 1;262:70-7. |  | Exclude | Irrelevant outcome - only risk factors, not related to the onset |
| Fornaro M, Daray FM, Hunter F, Anastasia A, Stubbs B, De Berardis D, Shin JI, Husain MI, Dragioti E, Fusar-Poli P, Solmi M. The prevalence, odds and predictors of lifespan comorbid eating disorder among people with a primary diagnosis of bipolar disorders, and vice-versa: systematic review and meta-analysis. Journal of affective disorders. 2021 Feb 1;280:409-31. |  | Exclude | No precursors/factors or signs mentioned |
| Foxhall M, Hamilton‐Giachritsis C, Button K. The link between rejection sensitivity and borderline personality disorder: A systematic review and meta‐analysis. British Journal of Clinical Psychology. 2019 Sep;58(3):289-326. |  | Exclude | Not a longitudinal design -Cross-sectional or case-control design |
| Fraguas D, Díaz-Caneja CM, Pina-Camacho L, Janssen J, Arango C. Progressive brain changes in children and adolescents with early-onset psychosis: A meta-analysis of longitudinal MRI studies. Schizophrenia research. 2016 Jun 1;173(3):132-9. |  | Exclude | only one relevant study Arango but Patients are already diagnosed - full blown bipolar |
| Frías Á, Palma C, Farriols N. Comorbidity in pediatric bipolar disorder: prevalence, clinical impact, etiology and treatment. Journal of affective disorders. 2015 Mar 15;174:378-89. |  | Exclude | Not a systematic review |
| Frías Á, Palma C, Farriols N. Neurocognitive impairments among youth with pediatric bipolar disorder: a systematic review of neuropsychological research. Journal of affective disorders. 2014 Sep 1;166:297-306. |  | Exclude | One longitudinal study (pavuluri) but patients are on medication and have full-blown BD |
| Frías Á, Palma C, Farriols N, González L. Sexuality‐related issues in borderline personality disorder: A comprehensive review. Personality and mental health. 2016 Aug;10(3):216-31. |  | Exclude | No longitudinal design -Cross-sectional or case-control design + patients are already diagnosed with BPD |
| Friborg O, Martinsen EW, Martinussen M, Kaiser S, Øvergård KT, Rosenvinge JH. Comorbidity of personality disorders in mood disorders: a meta-analytic review of 122 studies from 1988 to 2010. Journal of affective disorders. 2014 Jan 1;152:1-1. |  | Exclude | Prevalance study |
| Geller B, Luby J. Child and adolescent bipolar disorder: a review of the past 10 years. Journal of the American Academy of Child & Adolescent Psychiatry. 1997 Sep 1;36(9):1168-76. |  | Exclude | Not a systematic review- a literature review and no missed studies |
| Hartmann JA, Nelson B, Ratheesh A, Treen D, McGorry PD. At-risk studies and clinical antecedents of psychosis, bipolar disorder and depression: a scoping review in the context of clinical staging. Psychological medicine. 2019 Jan;49(2):177-89. |  | Exclude | Not an SR |
| Ibrahim J, Cosgrave N, Woolgar M. Childhood maltreatment and its link to borderline personality disorder features in children: A systematic review approach. Clinical child psychology and psychiatry. 2018 Jan;23(1):57-76. |  | Exclude | Duplicated data in another study |
| Laurens KR, Luo L, Matheson SL, Carr VJ, Raudino A, Harris F, Green MJ. Common or distinct pathways to psychosis? A systematic review of evidence from prospective studies for developmental risk factors and antecedents of the schizophrenia spectrum disorders and affective psychoses. BMC psychiatry. 2015 Dec;15(1):1-20. |  | Exclude | Sample analysed as pooled affective disorders |
| Marangoni C, Hernandez M, Faedda GL. The role of environmental exposures as risk factors for bipolar disorder: a systematic review of longitudinal studies. Journal of affective disorders. 2016 Mar 15;193:165-74. |  | Exclude | Irrelevant outcome - only risk factors, not related to the onset |
| McKay MT, Cannon M, Chambers D, Conroy RM, Coughlan H, Dodd P, Healy C, O’Donnell L, Clarke MC. Childhood trauma and adult mental disorder: A systematic review and meta‐analysis of longitudinal cohort studies. Acta Psychiatrica Scandinavica. 2021 Mar;143(3):189-205. |  | Exclude | No longitudinal studies with BD outcome |
| Parellada M, Gomez-Vallejo S, Burdeus M, Arango C. Developmental differences between schizophrenia and bipolar disorder. Schizophrenia Bulletin. 2017 Oct 21;43(6):1176-89. |  | Exclude | Not an SR |
| Pompili M, Girardi P, Ruberto A, Tatarelli R. Suicide in borderline personality disorder: a meta-analysis. Nordic journal of psychiatry. 2005 Jan 1;59(5):319-24. |  | Exclude | Different outcome + prevalence study |
| Porter C, Palmier‐Claus J, Branitsky A, Mansell W, Warwick H, Varese F. Childhood adversity and borderline personality disorder: a meta‐analysis. Acta Psychiatrica Scandinavica. 2020 Jan;141(1):6-20. |  | Exclude | Patients are already diagnosed with BPD |
| Rodriguez V, Alameda L, Trotta G, Spinazzola E, Marino P, Matheson SL, Laurens KR, Murray RM, Vassos E. Environmental risk factors in bipolar disorder and psychotic depression: a systematic review and meta-analysis of prospective studies. Schizophrenia bulletin. 2021 Jul;47(4):959-74. |  | Exclude | Irrelevant outcome - only risk factors, not related to the onset |
| Salagre E, Vizuete AF, Leite M, Brownstein DJ, McGuinness A, Jacka F, Dodd S, Stubbs B, Köhler CA, Vieta E, Carvalho AF. Homocysteine as a peripheral biomarker in bipolar disorder: a meta-analysis. European Psychiatry. 2017 Jun;43:81-91. |  | Exclude | Medications + doesnt say anything about the designs of the study, already diagnosed |
| Scott J, Murray G, Henry C, Morken G, Scott E, Angst J, Merikangas KR, Hickie IB. Activation in bipolar disorders: a systematic review. JAMA psychiatry. 2017 Feb 1;74(2):189-96. |  | Exclude | No longitudinal studies with emerging BD symptoms |
| Serafini G, Pompili M, Borgwardt S, Houenou J, Geoffroy PA, Jardri R, Girardi P, Amore M. Brain changes in early-onset bipolar and unipolar depressive disorders: a systematic review in children and adolescents. European child & adolescent psychiatry. 2014 Nov;23(11):1023-41. |  | Exclude | No longitudinal studies |
| Steele KR, Townsend ML, Grenyer BF. Parenting and personality disorder: An overview and meta-synthesis of systematic reviews. PloS one. 2019 Oct 1;14(10):e0223038. |  | Exclude | Not an SR- A review of reviews |
| Van Meter AR, Burke C, Kowatch RA, Findling RL, Youngstrom EA. Ten‐year updated meta‐analysis of the clinical characteristics of pediatric mania and hypomania. Bipolar disorders. 2016 Feb;18(1):19-32. |  | Exclude | No precursors/factors or signs mentioned |
| Van Meter AR, Burke C, Youngstrom EA, Faedda GL, Correll CU. The bipolar prodrome: meta-analysis of symptom prevalence prior to initial or recurrent mood episodes. Journal of the American Academy of Child & Adolescent Psychiatry. 2016 Jul 1;55(7):543-55. |  | Exclude | Pancheri has egeland 2012 so no need to analyse this paper |
| Wegbreit E, Cushman GK, Puzia ME, Weissman AB, Kim KL, Laird AR, Dickstein DP. Developmental meta-analyses of the functional neural correlates of bipolar disorder. JAMA psychiatry. 2014 Aug 1;71(8):926-35. |  | Exclude | no longitudinal studies |
| Winsper C, Marwaha S, Lereya ST, Thompson A, Eyden J, Singh SP. Clinical and psychosocial outcomes of borderline personality disorder in childhood and adolescence: a systematic review. Psychological medicine. 2015 Aug;45(11):2237-51. |  | Exclude | Irrelevant outcome - outcomes after receiving a diagnosis like changing schools, partner conflict etc or like early adolescent bor- derline symptoms predicted lower life satisfaction across two decades |
| Yapıcı Eser H, Taşkıran AS, Ertınmaz B, Mutluer T, Kılıç Ö, Özcan Morey A, Necef I, Yalçınay İnan M, Öngür D. Anxiety disorders comorbidity in pediatric bipolar disorder: a meta‐analysis and meta‐regression study. Acta Psychiatrica Scandinavica. 2020 Apr;141(4):327-39. |  | Exclude | Prevalence study |
| **HAND SEARCHED STUDIES** |  |  |  |
| Boucher MÈ, Pugliese J, Allard‐Chapais C, Lecours S, Ahoundova L, Chouinard R, Gaham S. Parent–child relationship associated with the development of borderline personality disorder: a systematic review. Personality and mental health. 2017 Nov;11(4):229-55. |  | Exclude | Duplicated data in another publication -One relevant longitudinal study but Stepp already has that one |
| Keinänen MT, Johnson JG, Richards ES, Courtney EA. A systematic review of the evidence-based psychosocial risk factors for understanding of borderline personality disorder. Psychoanalytic Psychotherapy. 2012 Mar 1;26(1):65-91. |  | Exclude | Irrelevant outcome - only risk factors, not related to the onset |
| Kessing LV, Willer I, Andersen PK, Bukh JD. Rate and predictors of conversion from unipolar to bipolar disorder: A systematic review and meta‐analysis. Bipolar Disorders. 2017 Aug;19(5):324-35. |  | Exclude | Duplicated data |
| Stead VE, Boylan K, Schmidt LA. Longitudinal associations between non-suicidal self-injury and borderline personality disorder in adolescents: a literature review. Borderline personality disorder and emotion dysregulation. 2019 Dec;6(1):1-2. |  | Exclude | Not an SR - literature review |
| Fusar-Poli P, Howes O, Bechdolf A, Borgwardt S. Mapping vulnerability to bipolar disorder: a systematic review and meta-analysis of neuroimaging studies. Journal of Psychiatry and Neuroscience. 2012 May 1;37(3):170-84. |  | Exclude | No longitudinal studies |
| Wu MK, Wang HY, Chen YW, Lin PY, Wu CK, Tseng PT. Significantly higher prevalence rate of asthma and bipolar disorder co-morbidity: a meta-analysis and review under PRISMA guidelines. Medicine. 2016 Mar;95(13). |  | Exclude | Prevalence rates |
| Scott J, McNeill Y, Cavanagh J, Cannon M, Murray R. Exposure to obstetric complications and subsequent development of bipolar disorder: systematic review. The British Journal of Psychiatry. 2006 Jul;189(1):3-11. |  | Exclude | Irrelevant outcome - only risk factors, not related to the onset |
| Tsuchiya KJ, Byrne M, Mortensen PB. Risk factors in relation to an emergence of bipolar disorder: a systematic review. Bipolar disorders. 2003 Aug;5(4):231-42. |  | Exclude | Irrelevant outcome - only risk factors, not related to the onset |
| Amad A, Ramoz N, Thomas P, Jardri R, Gorwood P. Genetics of borderline personality disorder: systematic review and proposal of an integrative model. Neuroscience & Biobehavioral Reviews. 2014 Mar 1;40:6-19. |  | Exclude | no longitudinal studies |
| Menculini G, Balducci PM, Attademo L, Bernardini F, Moretti P, Tortorella A. Environmental Risk Factors for Bipolar Disorders and High-Risk States in Adolescence: A Systematic Review. Medicina. 2020 Dec;56(12):689. |  | Exclude | Irrelevant outcome - only risk factors, not related to the onset |
| Skjelstad DV, Malt UF, Holte A. Symptoms and signs of the initial prodrome of bipolar disorder: a systematic review. Journal of affective disorders. 2010 Oct 1;126(1-2):1-3. |  | Exclude because van meter has already have egeland | Duplicated data |
| Yu H, Meng YJ, Li XJ, Zhang C, Liang S, Li ML, Li Z, Guo W, Wang Q, Deng W, Ma X. Common and distinct patterns of grey matter alterations in borderline personality disorder and bipolar disorder: voxel-based meta-analysis. The British Journal of Psychiatry. 2019 Jul;215(1):395-403. |  | Exclude | No longitudinal studies |
| Misiak B, Stramecki F, Gawęda Ł, Prochwicz K, Sąsiadek MM, Moustafa AA, Frydecka D. Interactions between variation in candidate genes and environmental factors in the etiology of schizophrenia and bipolar disorder: a systematic review. Molecular neurobiology. 2018 Jun;55(6):5075-100. |  | Exclude | No longitudinal studies |
| Dezhina Z, Ranlund S, Kyriakopoulos M, Williams SC, Dima D. A systematic review of associations between functional MRI activity and polygenic risk for schizophrenia and bipolar disorder. Brain imaging and behavior. 2019 Jun;13(3):862-77. |  | Exclude | No longitudinal studies |
| Bora E, Özerdem A. A meta-analysis of neurocognition in youth with familial high risk for bipolar disorder. European Psychiatry. 2017 Jul;44:17-23. |  | Exclude | No longitudinal studies |
| Mitchell AE, Dickens GL, Picchioni MM. Facial emotion processing in borderline personality disorder: a systematic review and meta-analysis. Neuropsychology review. 2014 Jun;24(2):166-84. |  | Exclude | No longitudinal studies |
| Howes OD, Lim S, Theologos G, Yung AR, Goodwin GM, McGuire P. A comprehensive review and model of putative prodromal features of bipolar affective disorder. Psychological medicine. 2011 Aug;41(8):1567-77. |  | Exclude | Not an SR |
| Luby JL, Navsaria N. Pediatric bipolar disorder: evidence for prodromal states and early markers. Journal of Child Psychology and Psychiatry. 2010 Apr;51(4):459-71. |  | Exclude | Not an SR |
| Szmulewicz A, Valerio MP, Martino DJ. Longitudinal analysis of cognitive performances in recent‐onset and late‐life Bipolar Disorder: A systematic review and meta‐analysis. Bipolar disorders. 2020 Feb;22(1):28-37. |  | Exclude | No predictors - Stability of cognitive performances |
| Hanford LC, Nazarov A, Hall GB, Sassi RB. Cortical thickness in bipolar disorder: a systematic review. Bipolar disorders. 2016 Feb;18(1):4-18. |  | Exclude | Participants receive medication and Janssen: participants with bipolar disorder with psychotic symptoms - early onset of psychosis not early onset of bipolar |
| Díaz-Caneja CM, Pina-Camacho L, Rodríguez-Quiroga A, Fraguas D, Parellada M, Arango C. Predictors of outcome in early-onset psychosis: a systematic review. npj Schizophrenia. 2015 Mar 4;1(1):1-0. |  | Exclude | Irrelevant outcome |
| Trotta A, Murray RM, MacCabe JH. Do premorbid and post-onset cognitive functioning differ between schizophrenia and bipolar disorder? A systematic review and meta-analysis. Psychological medicine. 2015 Jan;45(2):381-94. |  | Exclude | Parelleda has included all the prospective studies Trotta included |
| Wilde A, Chan HN, Rahman B, Meiser B, Mitchell PB, Schofield PR, Green MJ. A meta-analysis of the risk of major affective disorder in relatives of individuals affected by major depressive disorder or bipolar disorder. Journal of affective disorders. 2014 Apr 1;158:37-47. |  | Exclude | No relevant longitudinal study- Lau has Hammen and birmaher is not longitudinal and pilowsky is only about MDD |
| Rodriguez V, Alameda L, Trotta G, Spinazzola E, Marino P, Matheson SL, Laurens KR, Murray RM, Vassos E. Environmental risk factors in bipolar disorder and psychotic depression: a systematic review and meta-analysis of prospective studies. Schizophrenia bulletin. 2021 Jul;47(4):959-74. |  | Exclude | Irrelevant outcome - only risk factors, not related to the onset |

**Table S6.** Methodological quality of included systematic reviews and meta-analyses based on AMSTAR tool for Bipolar Disorder

| **Reference** | **AMSTAR question** | | | | | | | | | | | **TOTAL** |
| --- | --- | --- | --- | --- | --- | --- | --- | --- | --- | --- | --- | --- |
|  | **1** | **2** | **3** | **4** | **5** | **6** | **7** | **8** | **9** | **10** | **11** |  |
| Bart et al. (2021) | 0 | 0 | 1 | 0 | 0 | 1 | 0 | 0 | 0 | 0 | 1 | **3** |
| Brancati et al. (2021) | 1 | 1 | 1 | 0 | 0 | 1 | 0 | 0 | 1 | 0 | 1 | **6** |
| Cardoso et al. (2018) | 0 | 1 | 1 | 1 | 0 | 1 | 0 | 0 | 0 | 0 | 0 | **4** |
| Cahn et al. (2021) | 1 | 1 | 1 | 0 | 0 | 1 | 1 | 0 | 0 | 0 | 1 | **6** |
| Faedda et al. (2013) | 0 | 0 | 1 | 1 | 0 | 1 | 0 | 0 | 0 | 0 | 0 | **3** |
| Gibbs et al. (2015) | 0 | 1 | 1 | 1 | 0 | 1 | 1 | 1 | 1 | 1 | 1 | **9** |
| Hu et al. (2020) | 0 | 1 | 1 | 0 | 0 | 1 | 1 | 0 | 1 | 1 | 1 | **7** |
| Keramatian et al. (2022) | 0 | 1 | 0 | 1 | 0 | 1 | 0 | 0 | 0 | 0 | 1 | **4** |
| Lau et al. (2018) | 0 | 1 | 1 | 1 | 0 | 1 | 1 | 1 | 1 | 0 | 1 | **8** |
| Narayan et al. (2013) | 0 | 0 | 1 | 0 | 0 | 1 | 0 | 0 | 0 | 0 | 0 | **2** |
| Palmier-Claus et al. (2016) | 1 | 1 | 1 | 1 | 0 | 1 | 1 | 1 | 0 | 1 | 1 | **9** |
| Pancheri et al. (2019) | 0 | 1 | 1 | 1 | 0 | 1 | 1 | 1 | 0 | 0 | 0 | **6** |
| Rasic et al. (2014) | 0 | 1 | 1 | 0 | 0 | 1 | 0 | 0 | 1 | 1 | 1 | **6** |
| Ratheesh et al. (2011) | 0 | 1 | 1 | 1 | 0 | 1 | 1 | 1 | 1 | 1 | 1 | **9** |
| Ritter et al. (2011) | 0 | 0 | 1 | 0 | 0 | 1 | 0 | 0 | 0 | 0 | 0 | **2** |
| Scott et al. (2021) | 1 | 1 | 1 | 1 | 1 | 1 | 1 | 1 | 1 | 1 | 1 | **11** |
| Scott et al. (2022) | 1 | 1 | 1 | 1 | 1 | 1 | 1 | 1 | 1 | 0 | 1 | **10** |

**Table S7.** Methodological quality of included systematic reviews and meta-analyses based on AMSTAR tool for Borderline Personality Disorder

| **Reference** | **AMSTAR question** | | | | | | | | | | | **TOTAL** |
| --- | --- | --- | --- | --- | --- | --- | --- | --- | --- | --- | --- | --- |
|  | **1** | **2** | **3** | **4** | **5** | **6** | **7** | **8** | **9** | **10** | **11** |  |
| Skabeikyte & Barkauskiene (2021) | 1 | 1 | 1 | 1 | 0 | 1 | 1 | 1 | 0 | 0 | 1 | **9** |
| Stepp et al. (2016) | 1 | 1 | 1 | 1 | 0 | 1 | 0 | 0 | 0 | 0 | 0 | **5** |
| Winsper et al. (2016a) | 1 | 1 | 1 | 1 | 0 | 1 | 1 | 1 | 1 | 1 | 1 | **10** |
| Winsper et al. (2016b) | 1 | 1 | 1 | 1 | 0 | 1 | 1 | 1 | 0 | 0 | 1 | **8** |
| Winsper et al. (2017) | 1 | 1 | 1 | 1 | 0 | 1 | 1 | 1 | 1 | 1 | 1 | **10** |

**Table S8.** Citation Matrix for Systematic Reviews and Meta-Analyses on Bipolar Disorder onset. Systematic reviews are represented in columns and primary studies in rows. (+) indicates longitudinal primary studies contained in a systematic review and (-) studies not

included in a systematic review.

|  |  |  | **Systematic** | **Reviews** |  |  |  |  |  |  |  |  |  |  |  |  |  | **Total number of reviews (/11)** |
| --- | --- | --- | --- | --- | --- | --- | --- | --- | --- | --- | --- | --- | --- | --- | --- | --- | --- | --- |
| **Primary Study** | Bart et al. (2021) | Brancati et al. (2021) | Cahn et al. (2021) | Cardoso et al. (2018) | Faedda et al. (2013) | Gibbs et al. (2015) | Hu et al. (2020) | Keramatian et al. (2022) | Lau et al. (2018) | Narayan et al. (2013) | Palmier-Claus et al. (2016) | Pancheri et al. (2019) | Rasic et al. (2014) | Ratheesh et al. (2007) | Ritter et al. (2011) | Scott et al. (2021) | Scott et al. (2022) |  |
| Acuff et al. (2019) | **+** |  |  |  |  |  |  |  |  |  |  |  |  |  |  |  |  | 1 |
| Alloy et al. (2012) |  |  |  |  | **+** |  |  |  |  |  |  |  |  |  |  |  |  | 1 |
| Alloy et al. (2015) |  |  |  |  |  |  |  |  |  |  |  |  |  |  |  |  | + | 1 |
| Akiskal et al. (1977) |  |  |  |  | **+** |  |  |  |  |  |  |  |  |  |  |  |  | 1 |
| Akiskal et al. (1978) |  |  |  |  | **+** |  |  |  |  |  |  |  |  |  |  |  |  | 1 |
| Akiskal et al. (1983) |  |  |  |  | **+** |  |  |  |  |  |  |  |  | **+** |  |  |  | 2 |
| Akiskal et al. (1985) |  |  |  |  |  |  |  | **+** |  |  |  |  |  |  |  |  |  | 1 |
| Akiskal et al. (1995) |  |  |  |  | **+** |  |  |  |  |  |  |  |  | **+** |  |  |  | 2 |
| Anderson & Hammen (1993) |  |  |  |  |  |  |  |  | **+** |  |  |  |  |  |  |  |  | 1 |
| Angst et al. (2003) |  |  |  |  | **+** |  |  |  |  |  |  |  |  |  |  |  |  | 1 |
| Angst et al. (2005) |  |  |  |  |  |  |  |  |  |  |  |  |  | **+** |  |  |  | 1 |
| Arnold et al. 2020 |  | **+** |  |  |  |  |  |  |  |  |  |  |  |  |  |  |  | 1 |
| Axelson et al. (2011) |  |  |  |  | **+** |  |  |  |  |  |  |  |  |  |  |  |  | 1 |
| Axelson et al. (2015) |  | **+** |  |  |  |  |  | **+** |  |  |  |  |  |  |  |  |  | 2 |
| Bauwens et al. (1998) |  |  |  |  |  |  |  |  |  |  |  |  |  | **+** |  |  |  | 1 |
| Bechdolf et al. (2014) |  |  |  |  |  |  |  | **+** |  |  |  |  |  |  |  |  |  | 1 |
| Beesdo et al. (2009) |  |  |  |  | **+** |  |  |  |  |  |  |  |  | **+** |  |  |  | 2 |
| Benvenuti et al. (2008) |  |  |  |  |  |  |  |  |  |  |  |  |  | **+** |  |  |  | 1 |
| Bertocci et al. (2019) | **+** |  |  |  |  |  |  |  |  |  |  |  |  |  |  |  |  | 1 |
| Biederman et al. (1996) |  | **+** |  |  |  |  |  |  |  |  |  |  |  |  |  |  |  | 1 |
| Biedermann et al. (2008) |  | **+** |  |  |  |  |  |  |  |  |  |  |  |  |  |  |  | 1 |
| Biederman et al. (2009) |  |  |  |  |  |  |  |  |  |  |  |  |  | **+** |  |  |  | 1 |
| Biederman et al. (2014) |  |  |  |  |  |  |  |  |  |  |  |  |  | **+** |  |  |  | 1 |
| Birmaher et al. (2018) |  |  |  |  |  |  |  | **+** |  |  |  |  |  |  |  |  |  | 1 |
| Birmaher et al. (2021) |  |  |  |  |  |  |  | **+** |  |  |  |  |  |  |  |  |  | 1 |
| Bitter et al. (2011) |  |  | **+** |  |  |  |  |  |  |  |  |  |  |  |  |  |  |  |
| Boschloo et al. (2014) |  |  |  |  |  |  |  |  |  |  |  |  |  | **+** |  |  |  | 1 |
| Bukh et al. (2016a) |  |  |  | **+** |  |  |  |  |  |  |  |  |  |  |  |  |  | 1 |
| Bukh et al. (2016b) |  |  |  |  |  |  |  |  |  |  |  |  |  | **+** |  |  |  | 1 |
| Bromet et al. (2011) |  |  |  |  | **+** |  |  |  |  |  |  |  |  |  |  |  |  | 1 |
| Cassano et al. (2004) |  |  |  |  |  |  |  |  |  |  |  |  |  | **+** |  |  |  | 1 |
| Castro-Fornieles et al. (2011) |  |  |  |  | **+** |  |  |  |  |  |  |  |  |  |  |  |  | 1 |
| Chung et al. (2015) |  |  |  |  |  |  |  |  |  |  |  | **+** |  |  |  |  |  | 1 |
| Coryell et al. (1987) |  |  |  |  |  |  |  |  |  |  |  |  |  | **+** |  |  |  | 1 |
| Coryell et al. (1995) |  |  |  |  |  |  |  |  |  |  |  |  |  | **+** |  |  |  | 1 |
| Curry et al. (2011) |  |  |  |  |  |  |  |  |  |  |  |  |  | **+** |  |  |  | 1 |
| DeGeorge et al. (2014) |  |  |  |  |  |  |  | **+** |  |  |  |  |  |  |  |  |  | 1 |
| DelBello et al. (2003) |  |  |  |  | **+** |  |  |  |  |  |  |  |  | **+** |  |  |  | 2 |
| Doucette et al. (2013) |  |  |  |  |  |  |  |  |  |  |  |  |  |  |  |  | + | 1 |
| Duffy et al. (2007) |  | **+** |  |  |  |  |  |  |  | **+** |  |  |  |  | **+** |  |  | 3 |
| Duffy et al. (2009) |  |  |  |  |  |  |  |  |  |  |  |  | **+** |  |  |  |  | 1 |
| Duffy et al. (2010) |  |  |  |  |  |  |  |  |  |  |  |  |  |  | **+** |  |  | 1 |
| Duffy et al. (2012) |  |  |  |  |  | **+** |  |  |  |  |  |  |  |  |  |  |  | 1 |
| Duffy et al. (2014) |  |  |  |  |  |  |  |  | **+** |  |  | **+** |  |  |  |  | + | 3 |
| Duffy et al. (2019) |  |  |  |  |  |  |  | **+** |  |  |  |  |  |  |  | + | + | 3 |
| Dunn et al. (2006) |  |  |  |  |  |  |  |  |  |  |  |  |  | **+** |  |  |  | 1 |
| Egeland et al. (2003) |  |  |  |  |  |  |  |  |  |  |  |  |  |  | **+** |  | + | 2 |
| Egeland et al. (2012) |  |  |  |  |  |  |  | **+** |  |  |  | **+** |  |  |  | + | + | 4 |
| Fiedorowicz et al. (2011) |  |  |  |  | **+** |  |  |  |  |  |  |  |  | **+** |  |  |  | 2 |
| Fiedorowicz et al. (2012) |  |  |  |  |  |  |  |  |  |  |  |  |  | **+** |  | + |  | 2 |
| Findling et al. (2013) |  |  |  |  |  |  |  | **+** |  |  |  |  |  |  |  |  |  | 1 |
| Frankland et al. (2018) |  |  |  |  |  |  |  | **+** |  |  |  |  |  |  |  |  |  | 1 |
| Furukawa et al. (2000) |  |  |  |  |  |  |  |  |  |  |  |  |  | **+** |  |  |  | 1 |
| Furukawa et al. (2009) |  |  |  |  |  |  |  |  |  |  |  |  |  | **+** |  |  |  | 1 |
| Fusar Poli et al. (2018) |  |  |  |  |  |  |  | **+** |  |  |  |  |  |  |  |  |  | 1 |
| Ganzola et al. (2017) |  |  |  |  |  |  | **+** |  |  |  |  |  |  |  |  |  |  | 1 |
| Gan et al. (2011) |  |  |  |  |  |  |  |  |  |  |  |  |  | **+** |  |  |  | 1 |
| Garber et al. (1988) |  |  |  |  |  |  |  |  |  |  |  |  |  | **+** |  |  |  | 1 |
| Geller et al. (1994) |  |  |  |  |  |  |  |  |  |  |  |  |  | **+** |  |  |  | 1 |
| Geller et al. (2001) |  |  |  |  |  |  |  |  |  |  |  |  |  | **+** |  |  |  | 1 |
| Gilman et al. (2012) |  |  |  |  |  |  |  |  |  |  |  |  |  | **+** |  |  |  | 1 |
| Gilman et al. (2014) |  |  |  |  |  |  |  |  |  |  | **+** |  |  |  |  |  |  | 1 |
| Gogtay et al. (2007) |  |  |  |  | **+** |  |  |  |  |  |  |  |  |  |  |  |  | 1 |
| Goldberg et al. (1995) |  |  |  |  |  |  |  |  |  |  |  |  |  | **+** |  |  |  | 1 |
| Goldberg et al. (2001) |  |  |  |  |  |  |  |  |  |  |  |  |  | **+** |  |  |  | 1 |
| Hafeman et al. (2016) |  |  |  |  |  |  |  |  |  |  |  |  |  |  |  |  | + | 1 |
| Hafeman et al. (2017) |  |  |  |  |  |  |  | **+** |  |  |  |  |  |  |  |  |  | 1 |
| Hammen et al. (1990) |  |  |  |  |  |  |  |  | **+** |  |  |  |  |  |  |  |  | 1 |
| Halperin et al. 2011 |  | **+** |  |  |  |  |  |  |  |  |  |  |  |  |  |  |  | 1 |
| Henquet et al. (2006) |  |  |  |  |  | **+** |  |  |  |  |  |  |  |  |  |  |  | 1 |
| Hillegers et al. (2005) |  |  |  |  |  |  |  | **+** |  |  |  |  | **+** |  | **+** |  |  | 3 |
| Holma et al. (2008) |  |  |  |  |  |  |  |  |  |  |  |  |  | **+** |  |  |  | 1 |
| Homish et al. (2013) |  |  |  |  | **+** |  |  |  |  |  |  |  |  |  |  |  |  | 1 |
| Iorfino et al (2019) |  |  |  |  |  |  |  |  |  |  |  |  |  |  |  | + | + | 2 |
| Johnson et al. (1991) |  |  |  |  |  |  |  |  |  |  |  |  |  | **+** |  |  |  | 1 |
| Johnson et al. (2011) |  |  |  |  |  |  |  |  |  |  |  |  |  | **+** |  |  |  | 1 |
| Judd et al. (2013) |  |  |  |  |  |  |  |  |  |  |  |  |  | **+** |  |  |  | 1 |
| Kane et al. (1982) |  |  |  |  |  |  |  |  |  |  |  |  |  | **+** |  |  |  | 1 |
| Kaymaz et al. (2007) |  |  |  |  | **+** |  |  |  |  |  |  |  |  |  |  |  |  | 1 |
| Klein et al. (2021) |  | **+** |  |  |  |  |  |  |  |  |  |  |  |  |  |  |  | 1 |
| Klimnes-Dougan et al. (2010) |  |  |  |  |  |  |  |  |  | **+** |  |  |  |  |  |  |  | 1 |
| Klimnes-Dougan (2013) |  |  |  |  |  |  |  |  |  | **+** |  |  |  |  |  |  |  | 1 |
| Kovacs et al. (1994) |  |  |  |  |  |  |  |  |  |  |  |  |  | **+** |  |  |  | 1 |
| Kochman et al. (2005) |  |  |  |  | **+** |  |  |  |  |  |  |  |  |  |  |  |  | 1 |
| Kuehner et al. (2012) |  |  |  |  |  |  |  |  |  |  |  |  |  | **+** |  |  |  | 1 |
| Kwapil et al. (2000) |  |  |  |  | **+** |  |  |  |  |  |  |  |  |  |  |  |  | 1 |
| Laroche et al. (1987) |  |  |  |  |  |  |  |  |  |  |  |  | **+** |  |  |  |  | 1 |
| Levenson (2015) |  |  |  |  |  |  |  | **+** |  |  |  |  |  |  |  | + | + | 3 |
| Levenson et al. (2017) |  |  |  |  |  |  |  | **+** |  |  |  | **+** |  |  |  |  | + | 3 |
| Li et al. (2014) |  |  |  |  |  |  |  |  |  |  |  |  |  | **+** |  |  |  | 1 |
| Maj et al. (2007) |  |  |  |  |  |  |  |  |  |  |  |  |  | **+** |  |  |  | 1 |
| McCauley et al. (1993) |  |  |  |  |  |  |  |  |  |  |  |  |  | **+** |  |  |  | 1 |
| Melvin et al. (2013) |  |  |  |  |  |  |  |  |  |  |  |  |  | **+** |  |  |  | 1 |
| Mesman et al. (2013) |  |  |  |  |  |  |  | **+** |  |  |  |  |  |  |  |  |  | 1 |
| Mesman et al. (2017) |  |  |  |  |  |  |  |  |  |  |  | **+** |  |  |  | + | + | 3 |
| Nadkarni et al. (2010) |  |  |  |  | **+** |  |  |  |  |  |  |  |  |  |  |  |  | 1 |
| Nery et al. (2020) |  |  |  |  |  |  |  | **+** |  |  |  |  |  |  |  |  |  | 1 |
| Nurnberger et al. (2011) |  |  |  |  |  |  |  |  | **+** |  |  |  |  |  |  |  |  | 1 |
| Opjordsmoen et al. (1989) |  |  |  |  |  |  |  |  |  |  |  |  |  | **+** |  |  |  | 1 |
| Papachristou et al. (2012) |  |  |  |  |  |  |  |  |  |  |  |  |  |  |  |  | + | 1 |
| Papachristou et al. (2017) |  |  |  |  |  |  |  | **+** |  |  |  |  |  |  |  |  |  | 1 |
| Paaren et al. (2014) |  |  |  |  |  |  |  |  |  |  |  |  |  | **+** |  |  |  | 1 |
| Pfenning et al. (2016) |  |  |  | **+** |  |  |  |  |  |  |  |  |  | **+** |  | + | + | 4 |
| Perich et al. (2015) |  |  |  |  |  |  |  |  | **+** |  |  |  |  |  |  |  |  | 1 |
| Prien et al. (1984) |  |  |  |  |  |  |  |  |  |  |  |  |  | **+** |  |  |  | 1 |
| Preisig et al. (2016) |  |  |  |  |  |  |  | **+** |  |  |  |  |  |  |  |  |  | 1 |
| Radke-Yarrow et al. (1992) |  |  |  |  |  |  |  |  | **+** |  |  |  |  |  |  |  |  | 1 |
| Ratheesh et al. (2015) |  |  |  | **+** |  |  |  | **+** |  |  |  |  |  |  |  |  |  | 2 |
| Rao et al. (1995) |  |  |  |  |  |  |  |  |  |  |  |  |  | **+** |  |  |  | 1 |
| Rao (2002) |  |  |  |  |  |  |  |  |  |  |  |  |  |  |  | + | + | 2 |
| Reichart et al. (2005) |  |  |  |  |  |  |  | **+** |  |  |  |  |  |  | **+** |  |  | 2 |
| Regeer et al. (2006) |  |  |  |  | **+** |  |  |  |  |  |  |  |  |  |  |  |  | 1 |
| Riihimaki et al. (2011) |  |  |  |  |  |  |  |  |  |  |  |  |  | **+** |  |  |  | 1 |
| Ritter et al. (2015) |  |  |  |  |  |  |  |  |  |  |  | **+** |  |  |  | + | + | 3 |
| Rössler et al. (2011) |  |  |  |  | **+** |  |  |  |  |  |  |  |  |  |  |  |  | 1 |
| Rudaz et al. (2020) |  | **+** |  |  |  |  |  |  |  |  |  |  |  |  |  |  |  | 1 |
| Rudaz et al. (2021) |  |  |  |  |  |  |  | **+** |  |  |  |  |  |  |  |  |  | 1 |
| Ruggero et al. (2011) |  |  |  |  |  |  |  |  |  |  |  |  |  | **+** |  |  |  | 1 |
| Salvatore et al. (2009) |  |  |  |  | **+** |  |  |  |  |  |  |  |  |  |  |  |  | 1 |
| Salvatore et al. (2013) |  |  |  |  | **+** |  |  |  |  |  |  |  |  | **+** |  |  |  | 2 |
| Scott et al. (2017) |  |  |  |  |  |  |  |  |  |  |  |  |  |  |  | + | + | 2 |
| Scott et al. (2020) |  |  |  |  |  |  |  |  |  |  |  |  |  |  |  | + | + | 2 |
| Schwartz et al. (2000) |  |  |  |  |  |  |  |  |  |  |  |  |  | **+** |  |  |  | 1 |
| Sharma et al. (2014) |  |  |  |  |  |  |  |  |  |  |  |  |  | **+** |  |  |  | 1 |
| Shaw et al. 2005 |  |  |  |  |  |  |  |  |  |  |  |  |  |  | **+** |  | + | 2 |
| Shen et al. (2008) |  |  |  |  |  |  |  |  |  |  |  |  |  |  |  |  | + | 1 |
| Solomon et al. (1997) |  |  |  |  |  |  |  |  |  |  |  |  |  | **+** |  |  |  | 1 |
| Shur-Fen Gau et al. (2010) |  | **+** |  |  |  |  |  |  |  |  |  |  |  |  |  |  |  | 1 |
| Stoleru et al. (1997) |  |  |  |  |  |  |  |  |  |  |  |  |  |  |  |  | + | 1 |
| Strober et al. (1982) |  |  |  |  |  |  |  |  |  |  |  |  |  | **+** |  |  |  | 1 |
| Strober et al. (1993) |  |  |  |  | **+** |  |  |  |  |  |  |  |  | **+** |  |  |  | 2 |
| Tillman & Geller 2006 |  | **+** |  |  |  |  |  |  |  |  |  |  |  |  |  |  |  | 1 |
| Tjissen et al. (2010a) |  |  |  |  | **+** |  |  |  |  |  |  |  |  |  |  |  |  | 1 |
| Tijssen et al. (2010b) |  |  |  |  |  | **+** |  |  |  |  |  |  |  |  |  |  |  | 1 |
| Tohen et al. (2012) |  |  |  |  | **+** |  |  |  |  |  |  |  |  | **+** |  |  |  | 2 |
| Tondo et al. (2014) |  |  |  |  |  |  |  |  |  |  |  |  |  | **+** |  |  |  | 1 |
| Van Meter et al. (2021) |  |  |  |  |  |  |  | **+** |  |  |  |  |  |  |  |  |  | 1 |
| Weintraub (1987) |  |  |  |  |  |  |  |  |  | **+** |  |  |  |  |  |  |  | 1 |
| Weismann et al. (1999a) |  |  |  |  |  |  |  |  |  |  |  |  |  | **+** |  |  |  | 1 |
| Weismann et al. (1999b) |  |  |  |  |  |  |  |  |  |  |  |  |  | **+** |  |  |  | 1 |
| Winokur et al. (1973) |  |  |  |  |  |  |  |  |  |  |  |  |  | **+** |  |  |  | 1 |
| Winokur et al. (1987) |  |  |  |  |  |  |  |  |  |  |  |  |  | **+** |  |  |  | 1 |
| Zahn-Waxler et al. (1988) |  |  |  |  |  |  |  |  | **+** |  |  |  |  |  |  |  |  | 1 |
| Zimmerman et al. (2009) |  |  |  |  | **+** |  |  |  |  |  |  |  |  |  |  |  |  | 1 |

**Table S9***.*

Citation Matrix for Systematic Reviews and Meta-Analyses on Borderline Personality Disorder onset. Systematic reviews are represented in columns and primary studies in rows. (+) indicates longitudinal primary studies contained in a systematic review and (-) studies not included in a systematic review.

|  | **Systematic** | **Reviews** |  |  |  |  |  |
| --- | --- | --- | --- | --- | --- | --- | --- |
| **Primary Study** | Boucher et al. (2007) | Skabeikyte & Barkauskiene (2021) | Stepp et al. (2016) | Winsper et al. (2016a) | Winsper et al. (2016b) | Winsper et al. (2017) | **Total number of reviews (/6)** |
| Arens, Grabe, Spitzer, & Barnow (2011) | + |  |  |  |  |  | 1 |
| Barnow et al. (2013) |  | + | + |  |  |  | 2 |
| Belsky et al. (2012) |  |  | + | + | + |  | 3 |
| Bezirganian et al. (1993) | + |  | + |  |  |  | 2 |
| Bornolova (2009) |  |  |  | + |  |  | 1 |
| Bornolova et al. (2013)a |  |  | + |  | + |  | 2 |
| Bornolova et al. (2013b) |  |  | + |  |  |  | 1 |
| Bornolova et al. (2018) |  | + |  |  |  |  | 1 |
| Burke & Stepp (2012) |  |  | + |  |  |  | 1 |
| Carlson, Egeland, & Sroufe (2009) |  |  | + |  |  |  | 1 |
| Cohen et al. (2008) |  |  | + |  |  |  | 1 |
| Conway, Hammen, & Brennan (2015) |  |  | + |  |  |  | 1 |
| Crawford et al. (2009) |  |  | + |  |  |  | 1 |
| Crick et al. (2005) |  |  |  |  | + |  | 1 |
| Dixon-Gordon et al. (2016) |  | + |  |  |  |  | 1 |
| Ehrenreich, Beron, & Underwood (2016) |  | + |  |  |  |  | 1 |
| Greenfield et al. (2015) |  | + | + |  | + |  | 3 |
| Hallquist, Hipwell, & Stepp (2015) |  | + | + |  |  |  | 2 |
| Haltigan & Vaillancourt (2016) |  | + |  |  |  |  | 1 |
| Hammen, Bower, & Cole (2015) |  |  | + |  |  |  | 1 |
| Johnson et al. (2006) |  |  | + |  |  |  | 1 |
| Johnson et al. (1999) |  |  | + |  |  |  | 1 |
| Johnson et al. (2000) |  |  | + |  |  |  | 1 |
| Jovey et al. (2013) |  |  | + |  |  |  | 1 |
| Krabbendam et al. (2015) |  |  | + |  |  |  | 1 |
| Lazarus et al. (2019) |  | + |  |  |  |  | 1 |
| Lenzenweger & Desantis Castro (2005) |  |  | + |  |  |  | 1 |
| Lereya et al. (2016) |  |  |  |  |  | + | 1 |
| Lyons-Ruth et al. (2013) |  |  | + |  |  |  | 1 |
| Miller et al. (2008) |  |  | + |  |  |  | 1 |
| Meijer et al. (1988) |  |  |  |  | + |  | 1 |
| Ramklint et al. (2003) |  |  | + |  |  |  | 1 |
| Reinelt et al. (2014) |  |  | + |  |  |  | 1 |
| Rey et al. (1995) |  |  | + |  |  |  | 1 |
| Selby et al. (2013) |  |  |  |  |  | + | 1 |
| Selby & Yen/Yen et al. (2014) |  |  |  |  | + |  | 1 |
| Sharp et al. (2014) |  |  |  |  | + |  | 1 |
| Sharp et al. (2015) |  |  | + |  |  |  | 1 |
| Sharp et al. (2020) |  | + |  |  |  |  | 1 |
| Stepp, Burke, Hipwell, & Loeber (2012) |  |  | + |  |  |  | 1 |
| Stepp et al. (2013) |  |  | + |  |  |  | 1 |
| Stepp, Keenan, Hipwell, & Krueger (2014a) |  | + | + |  |  |  | 2 |
| Stepp et al. (2014b) |  | + | + |  |  |  | 2 |
| Stepp et al. (2015) |  |  | + |  |  |  | 1 |
| Stepp & Lazarus (2017) |  | + |  |  |  |  | 1 |
| Strandholm et al. (2017) |  | + |  |  |  |  | 1 |
| Thatcher, Cornelius, & Clark (2005) |  |  | + |  |  |  | 1 |
| Thomsen & Mikkelsen (1993) |  |  | + |  |  |  | 1 |
| Tragesser et al. (2010) |  |  | + |  |  |  | 1 |
| Tragesser, Solhan, Schwartz-Mette, Trull (2007) |  |  | + |  |  |  | 1 |
| Vaillancourt et al. (2014) |  |  |  |  | + |  | 1 |
| Vanwoerden, Leavitt, Gallagher & Temple (2019) |  | + |  |  |  |  | 1 |
| Widom, Czaja, & Paris (2009) |  |  | + |  |  |  | 1 |
| Winsper et al. (2012) |  |  | + |  | + |  | 2 |
| Winsper, Wolke, & Lereya (2015) |  |  | + |  |  |  | 1 |
| Wolke, Schreier, Zanarini & Winsper (2012) |  |  | + |  |  |  | 1 |

|  | Faedda et al. (2013) |  |  |  |  |  |  |  |  |  |  |  |  |  |  |  |  |  |
| --- | --- | --- | --- | --- | --- | --- | --- | --- | --- | --- | --- | --- | --- | --- | --- | --- | --- | --- |
| Ratheesh et al. (2007) | ~ 0.11% | Ratheesh et al. (2007) |  |  |  |  |  |  |  |  |  |  |  |  |  |  |  |  |
| Ritter et al. (2011) | 0% | 0% | Ritter et al. (2011) |  |  |  |  |  |  |  |  |  |  |  |  |  |  |  |
| Rasic et al. (2014) | 0% | 0% | 0.13% | Rasic et al. (2014) |  |  |  |  |  |  |  |  |  |  |  |  |  |  |
| Narayan et al. (2013) | 0% | 0% | 0.11% | 0% | Narayan et al. (2013) |  |  |  |  |  |  |  |  |  |  |  |  |  |
| Pancheri et al. (2019) | 0% | 0% | 0% | 0% | 0% | Pancheri et al. (2019) |  |  |  |  |  |  |  |  |  |  |  |  |
| Lau et al. (2018) | 0% | 0% | 0% | 0% | 0% | 0.08% | Lau et al. (2018) |  |  |  |  |  |  |  |  |  |  |  |
| Faedda et al. (2013) | 0% | ~0.11% | 0% | 0% | 0% | 0% | 0% | Faedda et al. (2013) |  |  |  |  |  |  |  |  |  |  |
| Palmier-Claus et al. (2016) | 0% | 0% | 0% | 0% | 0% | 0% | 0% | 0% | Palmier-Claus et al. (2016) |  |  |  |  |  |  |  |  |  |
| Gibbs et al. (2015) | 0% | 0% | 0% | 0% | 0% | 0% | 0% | 0% | 0% | Gibbs et al. (2015) |  |  |  |  |  |  |  |  |
| Hu et al. (2020) | 0% | 0% | 0% | 0% | 0% | 0% | 0% | 0% | 0% | 0% | Hu et al. (2020) |  |  |  |  |  |  |  |
| Cardoso et al. (2018) | 0% | ~0.02% | 0% | 0% | 0% | 0% | 0% | 0% | 0% | 0% | 0% | Cardoso et al. (2018) |  |  |  |  |  |  |
| Cahn et al. (2021) | 0% | 0% | 0% | 0% | 0% | 0% | 0% | 0% | 0% | 0% | 0% | 0% | Cahn et al. (2012) |  |  |  |  |  |
| Brancati et al. (2021) | 0% | 0% | 0.07% | 0% | 0% | 0% | 0% | 0% | 0% | 0% | 0% | 0% | 0% | Brancati et al. (2021) |  |  |  |  |
| Scott et al. (2021) | 0% | ~0.03% | 0% | 0% | 0.2% | ~0.21% | 0% | 0% | 0% | 0% | 0% | 0% | 0% | 0% | Scott et al. (2021) |  |  |  |
| Scott et al. (2022) | 0% | ~0.03% | ~0.08% | 0% | 0% | ~0.24% | ~0.04% | 0% | 0% | 0% | 0% | 0% | 0% | 0% | ~0.48% | Scott et al. (2022) |  |  |
| Keramatian et al. (2022) | 0% | 0% | ~0.04% | ~0.04% | 0% | ~0.04% | 0% | 0% | 0% | 0% | 0% | 0.04% | 0% | 0.3% | ~0.10% | 0.10% | Keramatian et al. (2022 |  |
| Bart et al. (2022) | 0% | 0% | 0% | 0% | 0% | 0% | 0% | 0% | 0% | 0% | 0% | 0% | 0% | 0% | 0% | 0% | 0% | Bart et al. (2022) |

Fig 1. Pairwise CCA for reviwes on Bipolar Disorder onset/symtpoms/prodrome. Colors indicate degree of overlap, as calculated with CCA. White = ≤5%, green 5.1–9.9% , yellow 10–14.9%

|  | Skabeikyte & Barkauskiene (2021) |  |  |  |  |  |
| --- | --- | --- | --- | --- | --- | --- |
| Stepp et al. (2016) | 0.10% | Stepp et al. (2016) |  |  |  |  |
| Winsper et al. (2016a) | 0% | 0.02% | Winsper et al. (2016a) |  |  |  |
| Winsper et al. (2016b) | 0.04% | ~%0.09 | 0.09% | Winsper et al. (2016b) |  |  |
| Skabeikyte & Barkauskiene (2021) | 0% | 0.10% | 0% | 0.04% | Skabeikyte & Barkauskiene (2021) |  |
| Winsper et al. (2017) | 0% | 0% | 0% | 0% | 0% | Winsper et al. (2017) |

Fig 1. Pairwise CCA for reviwes on Borderline Personality Disorder onset/symtpoms/prodrome. Colors indicate degree of overlap, as calculated with CCA. White = ≤5%, green 5.1–9.9% , yellow 10–14.9%
